# An integrative model links multiple inputs and signaling pathways to the onset of DNA synthesis in hepatocytes

**DOI:** 10.1111/j.1742-4658.2012.08572.x

**Published:** 2012-09

**Authors:** Jérémy Huard, Stephanie Mueller, Ernst D Gilles, Ursula Klingmüller, Steffen Klamt

**Affiliations:** 1 Max Planck Institute for Dynamics of Complex Technical SystemsMagdeburg, Germany; 2 Division Systems Biology of Signal Transduction, DKFZ-ZMBH Alliance, German Cancer Research Center (DKFZ)Heidelberg, Germany

**Keywords:** DNA synthesis, hepatocytes, integration, logical modeling, signaling pathways

## Abstract

During liver regeneration, quiescent hepatocytes re-enter the cell cycle to proliferate and compensate for lost tissue. Multiple signals including hepatocyte growth factor, epidermal growth factor, tumor necrosis factor α, interleukin-6, insulin and transforming growth factor β orchestrate these responses and are integrated during the G_1_ phase of the cell cycle. To investigate how these inputs influence DNA synthesis as a measure for proliferation, we established a large-scale integrated logical model connecting multiple signaling pathways and the cell cycle. We constructed our model based upon established literature knowledge, and successively improved and validated its structure using hepatocyte-specific literature as well as experimental DNA synthesis data. Model analyses showed that activation of the mitogen-activated protein kinase and phosphatidylinositol 3-kinase pathways was sufficient and necessary for triggering DNA synthesis. In addition, we identified key species in these pathways that mediate DNA replication. Our model predicted oncogenic mutations that were compared with the COSMIC database, and proposed intervention targets to block hepatocyte growth factor-induced DNA synthesis, which we validated experimentally. Our integrative approach demonstrates that, despite the complexity and size of the underlying interlaced network, logical modeling enables an integrative understanding of signaling-controlled proliferation at the cellular level, and thus can provide intervention strategies for distinct perturbation scenarios at various regulatory levels.

## Introduction

The liver is an essential organ with important roles in metabolism and detoxification. In the case of partial tissue loss due to intoxication or resection, it can undergo a remarkable regenerative process to maintain its function. Quiescent hepatocytes of the residual organ rapidly enter the G_1_ phase of the cell cycle to proliferate and compensate for the lost cell mass [1,2]. This highly synchronized response makes liver regeneration, and in particular hepatocytes, an attractive biological system to investigate proliferation control at the cellular level.

**Fig. 1:**
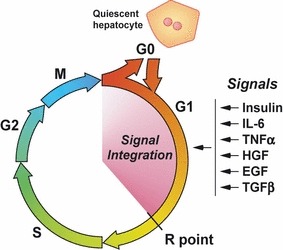
Multi-factorial control of the hepatocyte cell cycle. When the liver is challenged by intoxication or tissue loss a regenerative process is initiated. Usually quiescent hepatocytes exit quiescence (G_0_) and proliferate. A variety of signals including HGF, EGF, TNFα, IL-6, insulin and TGFβ orchestrate progression through the cell cycle. They are integrated during the mitogen-sensitive G_1_ phase of the cell cycle until the restriction point (R), where cells commit to proliferation and transition into S phase where DNA synthesis takes place.

Multiple signals from different sources within the body orchestrate progression through the regenerative process and are integrated during the mitogen-sensitive G_1_ phase of the hepatocyte cell cycle (Fig. 1). Among them are hepatocyte growth factor (HGF), epidermal growth factor (EGF), interleukin (IL)-6, tumor necrosis factor (TNF)α, insulin and transforming growth factor (TGF)β. HGF, the main liver mitogen, and EGF induce hepatocyte proliferation *in vivo* and *in vitro* [1]. IL-6 contributes to a variety of early responses, for example, the induction of a large number of immediate early genes (IEG) [3]. There has been considerable debate concerning whether IL-6 itself can induce proliferation because studies have yielded opposing results in the past [1,2,4–6]. TNFα and insulin do not trigger hepatocyte proliferation, but enhance the effect of mitogens [1]. TGFβ inhibits the proliferation of hepatocytes in culture and retains them in a quiescent state in the normal liver [7]. Furthermore, it plays an important role towards the end of liver regeneration *in vivo*, for example, in the reorganization of the regenerated tissue and establishment of the extracellular matrix [1].

Each of the described factors triggers individual signaling pathways and downstream responses that affect the hepatocyte’s fate. The different signals, however, do not act in a ‘one factor–one effect’ manner. The different inputs reach the cells simultaneously and consequently feed into an intertwined regulatory network [1]. This raises several questions: What is the individual contribution of each factor? How do the diverse stimuli affect each other and ultimately the cell cycle? Is there a safeguard in form of redundancy to guarantee completion of the regenerative process?

These issues are difficult to assess using traditional experimental approaches, which are limited in the number of mechanisms that can be investigated at once. To understand liver regeneration from a systems point of view it is, however, crucial to integrate the various regulatory circuits into a comprehensive picture. Here, systems biology that combines experimental data with mathematical modeling can elucidate the complex interplay of regulatory factors and mechanisms.

In the past, mathematical models of either signaling pathways [8–15] or the cell cycle [16–27] have mainly been developed separately from each other. As a consequence, the former usually used relevant kinases or transcription factors as end points without including detailed descriptions of downstream responses triggered by these factors. The latter primarily accounted for mitogenic stimulation only by the use of a single, simplified parameter, for example, growth or the presence of mitogen, and did not incorporate representations of upstream signaling events. To enable a comprehensive understanding of proliferation in response to multiple factors, integrative models that capture the complex interplay between signaling cascades and the cell cycle in more detail have to be developed [28]. First approaches have been made to connect signaling and cell-cycle progression in single models [29–31]. Yet, these studies achieved integration through drastic simplifications, for example, by lumping species and using single protein species to describe a biological phenotype such as DNA synthesis. Thus, comprehensive mathematical models integrating multiple signaling pathways that regulate cell proliferation with the cell cycle remain to be established.

The construction of interaction maps constitutes a starting point for most mathematical modeling approaches. Several groups have established such maps focusing on either a specific signaling pathway affecting the cell cycle [32–34] or the cell cycle itself with some connections to known players of signaling cascades [35–37]. These maps describe relationships between a great number of species, but are not directly useable to simulate the systems behavior [38]. Here, logic-based representations provide a solution that enables predictions, as well as analyses, of network responses and key properties. While the investigation of the qualitative dynamical behavior of logical models using, for example, the ginsim software [39] is restricted to small systems with up to 20 components [21–23,25,31], the analysis of large logical networks focuses on static features such as global interdependencies or qualitative input–output relationships.

An appropriate framework for static analyses of signaling and regulatory networks is based on logical interaction hypergraphs (LIH) [40,41], and has been successfully applied in various studies [11,13–15,42]. For example, by analyzing logical steady states (LSS) the qualitative response of a large network for a given set of stimuli can be calculated, and optimal perturbation targets to enforce a distinct network response can be identified [41]. For the latter, the concept of minimal intervention sets (MIS) has been introduced. A MIS describes a minimal combination of activations or inhibitions of certain species that leads to a predefined effect [41,43], for example, blocking of DNA synthesis. Furthermore, structural sensitivity analysis [13] can be performed to elucidate whether distinct reactions are particularly important to obtain a desired input–output relationship or whether the network inherits distinct properties like robustness to structural changes.

In this study, we used this framework to build a comprehensive large-scale logical model that integrates the influence of various signals (inputs) with the regulation of the cell cycle in hepatocytes. The model’s predictive power was assessed by comparison with literature-derived and our own experimental data, allowing subsequent model refinement by suitable and biologically justified modifications. Using MIS analysis we were able to: (a) elucidate the importance of the phosphatidylinositol 3-kinase (PI3K) and mitogen-activated protein kinase (MAPK) pathways for proliferation; (b) identify key species in this pathway; (c) predict genetic alterations with malignant consequences, which we verified using a mutation database; and (d) propose and experimentally validate inhibition targets to prevent DNA synthesis in HGF-stimulated hepatocytes.

## Results

### A logical multilevel model to capture the impact of multiple signaling inputs on the G_1_/S transition of the mammalian cell cycle

**Fig. 2:**
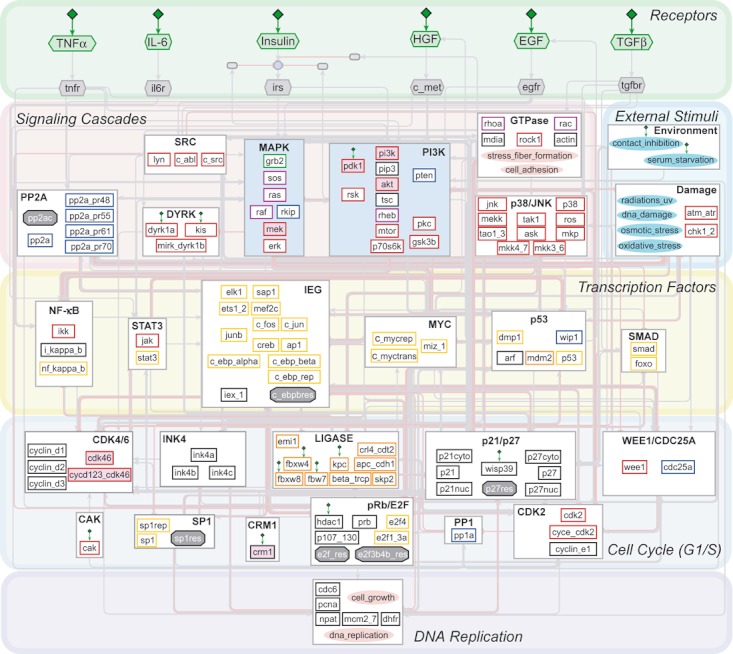
Schematic representation of the integrated large-scale logical model. The model comprises six layers of regulation indicated by different colors: Receptors (green), Signaling Cascades (rose), External Stimuli (turquoise), Transcription Factors (yellow), Cell Cycle (G_1_/S) (light blue) and DNA Replication (dark blue). Each layer contains modules (gray boxes) describing the activation or inhibition of certain species or families of species, e.g. belonging to a distinct signaling pathway. The MAPK and PI3K modules for which a specific model analysis has been conducted are highlighted with light blue fill. Activation processes are denoted as black arrows, while red edges represent inhibitory reactions. Green hexagons symbolize inputs (ligands), light gray ones with black font indicate transmembrane receptors, and dark gray ones with white font represent reservoirs. Rectangles with colored rims represent kinases (red), phosphatases (blue), transcription factors (yellow), ubiquitin ligases (orange), GTPases and guanine nucleotide exchange factors (GEFs) (purple), adaptor proteins (green), and others (black). Model species subjected to inhibitor treatments for experimental validation purposes within this study are highlighted with purple fill. Turquoise ovals represent environmental stimuli, and rose-colored ones phenotypes. The model contains 245 species including 104 auxiliary (dummy) (Materials and methods) and comprises 417 reactions. For details regarding interconnectivity please refer to Doc. S2. The model is provided in promot format in the Supporting information. Furthermore, the cellnetanalyzer model files are available from the model repository of this software at the following website: http://www.mpi-magdeburg.mpg.de/projects/cna/repository.html.

Here, we present a logical model that integrates the influence of multiple signals (inputs) with the regulation of the cell cycle in hepatocytes. For its construction we used the LIH formalism, which is directly supported by the software tools promot [44] and cellnetanalyzer [40] (see Materials and methods). Its structure was based on knowledge derived from the established literature and available dynamical models of the mammalian cell cycle, and documentation for all reactions can be found in the Supporting information (Doc. S2). The model is organized in a modularized fashion to delineate functional units and contains a total of 27 modules distributed over five layers of regulation (Fig. 2).

Six inputs for essential signals of the liver-regeneration process (TNFα, IL-6, insulin, HGF, EGF/TGFα and TGFβ) were incorporated. Activation of their corresponding transmembrane receptors is captured in the layer ‘Receptors’. It should be noted that the integrated model presented here does not distinguish between EGF and TGFα stimulation, because: (a) both ligands signal via the same receptor (epidermal growth factor receptor), and (b) the model does not reproduce specific dynamic signaling behavior arising from either ligand. Activation of transmembrane receptors leads to signal propagation described in the layer ‘Signaling Cascades’. This section contains simplified pathway representations of protein phosphatase (PP) 2A, SRC kinase family, dual-specificity tyrosine phosphorylation-regulated kinase (DYRK), MAPK, PI3K, GTPase and p38/c-Jun N-terminal kinase (JNK) signaling. The respective modules are not as comprehensive as specific models of individual pathways such as the logical models of EGF [14] and TNFα [15] signaling. Because our approach aims at integrating not only a single pathway, but the majority of signaling cascades important in the liver regeneration process with the mitogen-sensitive G_1_/S cell-cycle phase, we used qualitative pathway descriptions that provide all relevant connections to allow coupling to each other, and also to adjacent model layers. Signal propagation in ‘Signaling Cascades’ leads to activation of downstream targets described in the layer ‘Transcription Factors’ that contains modules for the important species nuclear factor kappa B (NFκB), signal transducer and activator of transcription (STAT) 3, MYC, p53, mothers against decapentaplegic homologs (SMAD) and immediate early genes (IEG). It is directly connected to the ‘Cell Cycle (G_1_/S)’ section thus linking multiple input signaling and cell-cycle regulation. ‘Cell Cycle (G_1_/S)’ describes progression through the G_1_ phase of the mammalian cell cycle until the G_1_/S transition. It feeds directly into the layer ‘DNA Replication’ that describes the onset of DNA synthesis, which is the single model output. Lastly, the model contains ‘External Stimuli’ that incorporate additional inputs in case of unfavorable environmental conditions, such as contact inhibition, serum starvation or cellular damage.

Although most nodes are represented as Boolean variables [38], some are described using multivalued logic to account for step-wise activation or inactivation processes of species important in positive feedback loops. This concept enables a single species to perform different functions depending on its current activation state. For example, in the layer ‘Cell Cycle (G_1_/S)’ the species retinoblastoma protein (Rb), E2F, nuclear p21 and CDC25A can display three activation levels (‘0’, ‘1’ and ‘2’), whereas nuclear p27 and cyclin E : cyclin-dependent kinase (CDK)2 can feature four possible states (‘0’, ‘1’, ‘2’ and ‘3’) (Doc. S2). To allow computation of initial network responses in the presence of feedback loops two time scales were introduced that allow distinguishing early from late events (Materials and methods).

The complete integrated logical model included 141 species, as well as 104 dummy and reservoir species simplifying model representation (Materials and methods). It contained 417 interactions or hyperedges, i.e. ‘AND’ combinations of species that lead to the activation of another species [41]. Thirty seven interactions were set to time scale ‘2’.

### Comparison of model behavior and hepatocyte-specific literature data indicates necessary model improvement

Our integrated logical model has been constructed upon established literature knowledge derived from a variety of mammalian cell types. To evaluate its performance in a hepatocyte-specific context we compared model predictions with selected literature scenarios of this cell type.

As a first reference, we compared our network with a detailed logical model of EGF receptor signaling [14] that comprised 104 species and 204 interactions, and had been validated using high-throughput primary human hepatocyte data. Because this model related to only a small part of our network, we focused on the comparison of model simulations for shared species rather than the model topologies. Of the 29 species in common, predictions for 28 showed agreement between the two models when simulating EGF stimulation. The simulation for the species *pp2a* differed, however. To allow signal propagation in the model, this specific node must be disabled, which can be realized in different ways. In the model of Samaga *et al.* [14], the state of *pp2a* was set to ‘0’ (inactive), whereas in our model its activity was moved to a separate time scale ‘2’. The good agreement between predictions for our integrated network and the EGF receptor model indicated a sufficient quality of the simplified EGF-induced signaling pathway employed in our model.

**Fig. 3:**
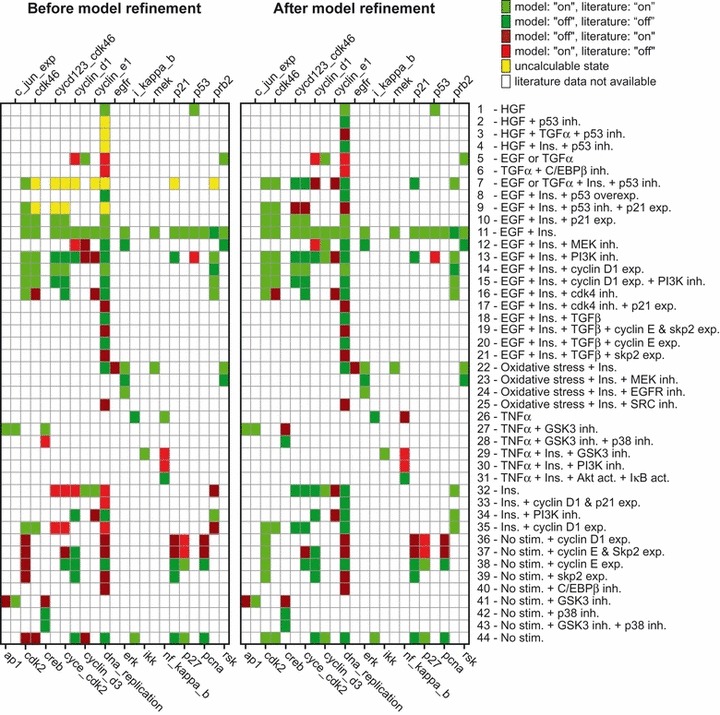
Comparison between model predictions and selected literature dataset. A murine hepatocyte-specific literature dataset comprising 44 scenarios (vertical axis) and 23 species (horizontal axes) was selected and used for comparison with model predictions to qualitatively judge its predictive power. A detailed description of this dataset with all references can be found in Doc. S3. Concordance between model predictions and literature dataset is color-coded (upper right-hand corner). (Left) Comparison of selected literature and initial large-scale model before model refinement; 60% of all observations were predicted correctly, 30.6% differed and a logical steady state was incalculable in 9.4% of the cases. (Right) Comparison of selected literature dataset and refined large-scale model; 76.3% of all observations were predicted correctly, 23.7% differed and no incalculable states remained. act, activation; inh., inhibition; Ins., insulin; overex., overexpression; exp, expression; No stim., no stimulation.

We furthermore selected literature scenarios of normal (wild-type) or perturbation studies conducted in primary murine hepatocytes under various stimulation conditions for a comparison. A detailed list of the selected 44 scenarios comprising 160 measurements in total can be found in the Supporting information (Doc. S3). LSS computation for the respective scenarios at time scale ‘1’ (Materials and methods) revealed an agreement of only 60.0% between model predictions and literature data; 30.6% of the scenarios differed, and for 9.4% a unique response could not be calculated from the logical relationships because of indeterminacy (Fig. 3, left). This relatively large percentage of incorrect predictions and incalculable responses suggested an optimization of the model structure to improve the predictive power.

### Model improvements allow full determination of all species

To eliminate incalculable states, we searched for suitable model improvements. There are three possibilities that prevent a fully determinable LSS: (a) an input has not been defined, (b) incomplete truth table (ITT) gates [41] exist, or (c) feedback loops prevent steady-state computation (Materials and methods). In our case, all inputs were defined, thus eliminating the first possibility. The two species *stabil_p21* and *foxo_sp1* were regulated by ITT gates. Closer inspection of the scenarios in which a unique response could not be calculated revealed that in each case the tumor suppressor p53 was inhibited. In the context of p53 inhibition upon HGF stimulation only the species *foxo_sp1* could not be determined. However, imposing a value (‘0’ or ‘1’) did not improve steady-state computation, showing that ITT gates were not the source of incalculable states. Consequently, the presence of feedback loops circumvented a full determination of all model species. This furthermore showed that not all loops were disrupted by the assignment of time scale ‘2’ for 37 reactions to allow computation of the initial response.

We investigated more closely the interactions between the 59 undetermined species that emerged when simulating HGF-mediated responses in the presence of p53 inhibition. This small subnetwork contained 5635 feedback loops of which 49.5% were negative, indicating a high interconnectivity between its components. Because of this property, there might exist central components that are members of various loops for which imposing a value is sufficient to determine a unique state of all remaining species. To identify them, we used the concept of MIS [41,43] (Materials and methods) on a scenario in which inhibition of p53 prevented the induction of DNA synthesis although HGF was present [45]. We computed the MIS of size ‘1’ and selected solutions leading to full determination of the model’s steady state. Because we searched for a solution that did not modify all other scenarios, we reduced the number of candidates by retaining only species directly regulated by p53. Based on this selection, only imposing a value for *sp1rep* representing the repressive form of the specificity protein 1 (Sp1) transcription factor possessed the potential to allow a full determination of all species in the case of p53 inhibition. Because this solution was impeded by a negative feedback loop between *prb* and *sp1rep*, we consequently moved this loop to another time scale thus allowing a complete solution for all species.

To further improve accordance of model predictions and selected literature scenarios we identified deviations between them and searched for suitable and biologically justifiable modifications of the network structure. Although our model predicted *cdk2*, *cdk46* and *cyclin_d3* to be absent during quiescence, literature data showed that their corresponding proteins were detectable in unstimulated hepatocytes (scenario 44, Doc. S3). Hence, their regulation was removed from the model and their default value set to ‘1’. We also searched for input and observation pairs showing inconsistencies of their regulation in model and literature scenarios. However, no candidate with a proximity of ‘1’ (direct regulation) was identified that would have allowed straightforward and unique changes.

The described modifications improved the accordance between model predictions and selected literature scenarios to 71.3% and reduced their deviation to 28.7%, while no incalculable states remained (Fig. S1).

### Comparison of model predictions and experimental DNA synthesis data shows discrepancies in the input-output behavior

**Fig. 4:**
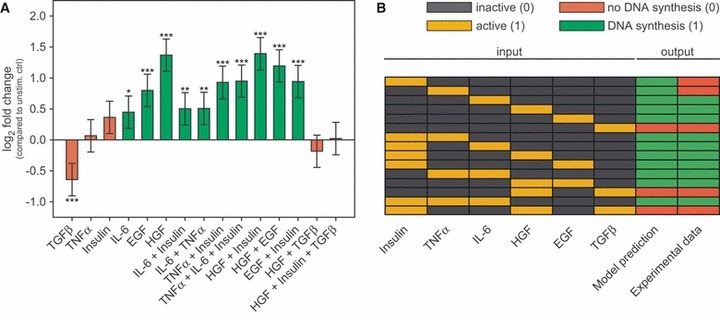
Comparison between model predictions for DNA synthesis and experimental data. (A) Log_2_ fold change in DNA content compared with unstimulated control. Primary mouse hepatocytes were cultivated at subconfluency in serum-free cultivation medium and stimulated with 1 ng·mL^−1^ TGFβ, 20 ng·mL^−1^ TNFα, 100 nm insulin, 40 ng·mL^−1^ IL-6, 50 ng·mL^−1^ EGF, 40 ng·mL^−1^ HGF, combinations thereof or were left untreated for 48 h. DNA content was subsequently determined using a Sybr® Green I assay. Data obtained in four independent experiments were used for analysis. A significant increase in DNA content was identified using a statistical mixed linear model (Materials and methods). The significance threshold for *P*-values was set to 0.05. Error bars represent 95% confidence intervals. *0.05 > *P* > 0.01; **0.01 > *P* > 0.001; ***P < 0.001. Values of *P* can be found in Doc. S5. To allow comparison with model predictions data were discretized based upon the statistical analysis. Treatments with a positive and statistically significant log_2_ fold change were assigned a binary state of ‘1’ (green bars). Treatments with a statistically not significant or negative log_2_ fold change were assigned a state of ‘0’ (red bars). (B) Comparison of model predictions for DNA synthesis with experimental data. Each row represents the comparison of the model prediction for the output (DNA synthesis) of a distinct input (ligand) combination with the experimental DNA synthesis data. Inputs in orange are present (active or ‘1’), whereas gray ones are absent (inactive or ‘0’). A green output indicates induction of DNA synthesis, whereas red outputs equal absence of DNA replication. Only two scenarios were incorrectly predicted by the model: insulin and TNFα alone did not trigger DNA synthesis *in vitro*.

We next sought to validate the input (presence of ligands)–output (DNA synthesis) behavior of the model for primary mouse hepatocytes. A set of standardized experiments was performed to measure the induction of DNA synthesis upon stimulation with a variety of ligand combinations. Primary mouse hepatocytes were isolated and cultivated at subconfluency under serum-free conditions. Cells were stimulated with HGF, EGF, IL-6, TNFα, insulin, TGFβ or combinations thereof for 48 h. Ligand concentrations were chosen according to the established literature to ensure robust activation of the canonical pathways [46]. Following stimulation for 48 h, cells were assayed for DNA content using a Sybr® Green I-based assay. For each of the tested stimuli the log_2_ fold change in DNA content after 48 h of stimulation is plotted in Fig. 4A. A value of ‘1’ corresponds to a doubling of the genetic information. We used a statistical mixed linear model to identify whether DNA synthesis was significantly induced for a given input compared with the unstimulated control [47] (Materials and methods). This procedure accounts for technical variability within a single experiment and biological variability between different experiments. As statistical threshold we employed a common *P*-value of 0.05. The DNA content of untreated controls did not change over time (Fig. S2). HGF and EGF proved to be the most potent mitogens in our assay. Also IL-6, though to a much smaller extent, was capable of inducing DNA synthesis. TNFα and insulin seemed to trigger a slight increase in DNA content. However, these changes were not statistically significant.

Model predictions were compared with these experimental results by computing the LSS for the respective input scenarios, and discretizing the experimental dataset based on our statistical analysis. A significant, positive induction of DNA synthesis in our experiments was interpreted as a binary state of ‘1’, whereas no significant or negative induction corresponded to ‘0’ (Fig. 4). Prevention of DNA synthesis by adding TGFβ to any ligand combination was predicted correctly by the model, as a complete block of DNA replication could be seen when combining TGFβ with HGF or with HGF and insulin in our assay. Stimulation with TGFβ alone resulted in a negative fold change indicating a certain degree of cell death in addition to the block of proliferation. However, our network does not represent this effect, because the only possible model outputs are presence or absence of DNA synthesis. The model identified HGF and EGF, as well as IL-6 alone or in combination with insulin or TNFα as sufficient for triggering DNA synthesis, which was in line with our experimental observations. However, in contrast to our model predictions, TNFα and insulin alone did not trigger DNA synthesis in primary mouse hepatocytes, but were capable of doing so when applied in combination. Because of this deviation the input–output behavior could be only partially validated and further model improvements were required.

### Activation of PI3K and MAPK signaling are necessary and sufficient for mitogen-dependent proliferation

To enable specific improvements that allow reproduction of the experimental data by our model, we searched for species and modules whose regulation was essential to the induction of DNA synthesis.

We used the approach of MIS to find interventions in the network that induced DNA synthesis when all inputs were set to ‘0’. The results are depicted in Fig. S3. Apart from transmembrane receptors for all ligands except TGFβ, MIS of size ‘1’ comprised the janus kinase (*jak*), the adapter protein growth factor receptor-bound protein (*grb*)*2*, the guanidine nucleotide exchange factor *sos*, the GTPase *ras*, *pi3k* and the signaling molecule phosphatidylinositol-(3,4,5)-triphosphate (*pip3*). These species hence conferred the ability to trigger DNA synthesis to their respective modules, i.e. the Janus kinase (JAK), PI3K and MAPK pathways.

**Fig. 5:**
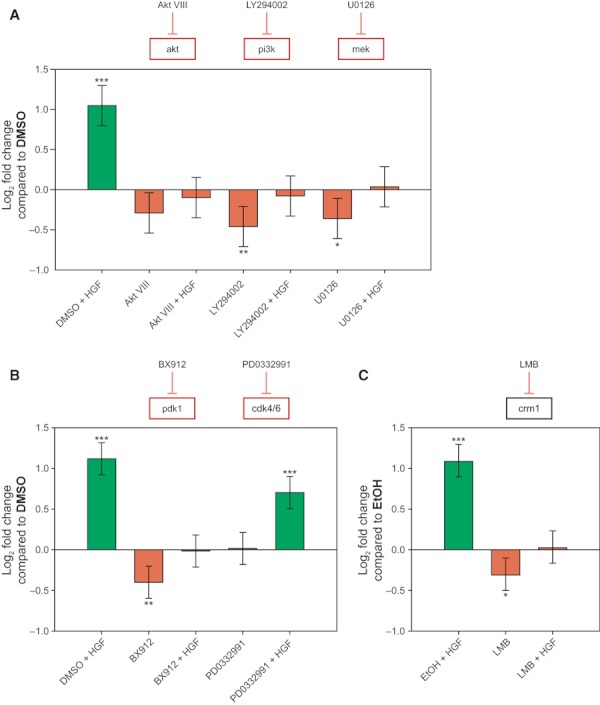
Experimental validation of model predictions. Log_2_ fold change in DNA content compared with solvent control. Primary mouse hepatocytes were stimulated with 40 ng·mL^−1^ HGF and 10 μm Akt inhibitor VIII or 10 μm LY294002 or 10 μm U0126 (all A), or 15 μm BX912 or 2 μm PD0332991 (both B), or 1 nm LMB (C) for 48 h. DNA content was subsequently determined using a Sybr® Green I assay. Data obtained in three (B,C) or four (A) independent experiments were used for statistical analysis. A significant increase in DNA content compared to solvent controls [ethanol (EtOH) for LMB, dithmethylsulfoxide (DMSO) for all other inhibitors) was identified using a statistical mixed linear model (Materials and methods). The significance threshold for *P*-values was set to 0.05. Error bars represent 95% confidence intervals. *0.05 > *P* > 0.01; **0.01 > *P* > 0.001; ***P < 0.001. Values of *P* can be found in Doc. S5. To allow comparison with model predictions, data were discretized based upon the statistical analysis. Treatments with a positive and statistically significant log_2_ fold change were assigned a binary state of ‘1’ (green bars). Treatments with a statistically not significant or negative log_2_ fold change were assigned a state of ‘0’ (red bars).

We further investigated the relationship between these three signaling cascades by analyzing their truth table for the induction of DNA synthesis (Table 1). When activating only one of the three modules and simultaneously inhibiting either the PI3K or MAPK pathway, DNA synthesis was blocked. From this observation, three conclusions could be drawn: (a) both the PI3K and the MAPK pathway must be activated for mitogen-mediated induction of DNA synthesis, (b) the PI3K and MAPK signaling pathways activate each other by cross-talk, and (c) JAK triggers DNA synthesis via activation of PI3K and MAPK. To experimentally validate that coactivation of the PI3K and MAPK modules is required for induction of DNA synthesis, we employed small molecule inhibitors against central components of these pathways (Fig. 2). We treated primary mouse hepatocytes with either protein kinase B (Akt) inhibitor VIII, LY294002 or U0126 targeting Akt1/2, PI3K or MAPK kinase (MEK)1/2, respectively. In line with our model hypothesis, inhibition of either the PI3K or MAPK pathway was sufficient to prevent HGF-induced DNA synthesis (Fig. 5A).

**Table 1:** Truth table describing the effects of the JAK, PI3K and MAPK pathways on DNA synthesis. Setting the PI3K pathway module to ‘1’ means that only *pi3k* adopts the state ‘1’, whereas setting it to ‘0’ means that all PI3K pathway members are switched to ‘0’ to avoid cross-induction by active MAPK. The same concept applies to the MAPK module, where only the species *grb2* is set to ‘1’ in case of activation, while the whole MAPK cascade adopts a state of ‘0’ in case of inhibition. An asterisk (*) indicates that no input value was imposed, which can however be set after logical steady-state computation by cross-talk

Inputs			Output
JAK	PI3K	MAPK	DNA synthesis
1	*	*	1
*	1	*	1
*	*	1	1
*	1	0	0
*	0	1	0
1	*	0	0
1	0	*	0

In order to determine the species of the MAPK and PI3K pathways required for an induction of DNA synthesis, we again used MIS analysis. However, in this case, we added constraints. In a simple network *A* → *B* → *C*, if activation of *C* was the goal of MIS calculation, both activation of *A* or *B* would be MIS candidates. However, activation of *A* is a candidate only because of the subsequent activation of *B*. When the states of *A* and *B* are fixed to their respective steady-state values in the absence of stimulation (i.e. *A* and *B* would be ‘0’), activation of *B* remains the only possible MIS of size ‘1’. Using these constraints for species of the PI3K and MAPK modules and by allowing interventions only on these two pathways and not on the rest of the network, we found a unique MIS of size ‘5’ comprising the activation of *akt*, *pkc*, *erk* and *s6k*, as well as inhibition of *gsk3b*. We thus identified these five species to be essential in mediating induction of DNA synthesis through the MAPK and PI3K pathways.

### Refinement of model structure regarding activation of PI3K and MAPK modules allows reproduction of experimental data

**Fig. 6:**
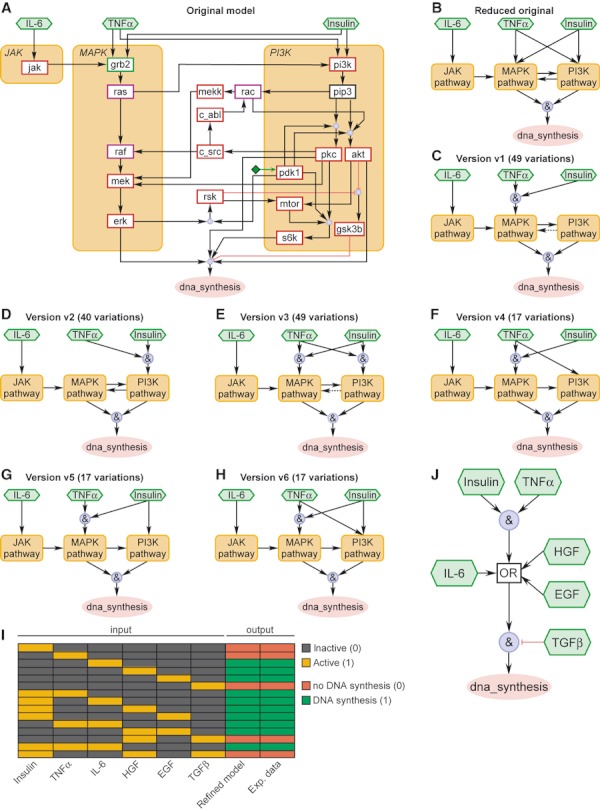
Input-specific model refinement. (A) Original model of cross-talks between the JAK, MAPK and PI3K pathways and their influence on DNA synthesis extracted from the integrated logical model. Activation processes are denoted as black arrows, whereas red edges represent inhibitory reactions. Blue circles symbolize ‘AND’ gates. Green hexagons symbolize inputs (ligands). Rectangles with colored rims represent kinases (red), GTPases and guanine nucleotide exchange factors (purple), adaptor proteins (green) and others (black). Orange areas indicate affiliation with a specific pathway. (B) Reduced representation of the model depicted in (A). (C–H) Model versions based on modification of the reduced model in (B) that were capable of explaining the experimental data in Fig. 4A. Only modification or removal of existing edges was allowed. No new edges were included. Each model version represents a set of model variations describing all possible cross-talk combinations. (I) Comparison between model predictions for DNA synthesis and experimental data after model refinement. Each row represents the comparison of the model prediction for the output (DNA synthesis) for a distinct input (ligand) combination with the experimental DNA synthesis data. Inputs in orange are present (active or ‘1’), whereas gray ones are absent (inactive or ‘0’). A green output indicates induction of DNA synthesis, whereas red outputs equal the absence of DNA replication. (J) Logical representation of the input hierarchy derived from the refined model valid for the presented experimental system and conditions (see also Fig. S4). TGFβ has priority over any pro-proliferative signal.

We next identified minimal model changes regarding activation of the PI3K and MAPK pathways by TNFα and insulin that led to a full reproduction of our experimental DNA synthesis data. For this purpose, we extracted the cross-talk between the JAK, PI3K and MAPK modules from our integrated logical model (Fig. 6A) to optimize their interconnection. A simplification of the original subnetwork (Fig. 6B) served as starting point to develop different model versions in which only removal or rearrangement of nodes involved in the Boolean expression of an interaction were allowed. Each model version represents a set of model variations, as different combinations of edges can result in the same simplified structure. In total 159 model variations grouped into six versions (Fig. 6C–H) could explain our DNA synthesis data.

Our experimental data showed that IL-6 triggered DNA synthesis in primary mouse hepatocytes. From our analysis, we knew that the PI3K and MAPK pathways had to be activated to allow this induction. Hence, MAPK activation by JAK that is a target downstream of IL-6, and at least one link between the MAPK and PI3K cascades were necessary to enable induction of DNA synthesis by IL-6.

To capture the cooperative effect of TNFα and insulin on DNA synthesis, at least one ‘AND’ gate was necessary to allow activation of the MAPK and PI3K pathways only in the presence of both ligands. Several solutions are possible to achieve this modification: (a) an improved model with minimal changes and these key features is represented by one variation of version v1 (Fig. 6C), where insulin and TNFα together induce MAPK activity, and PI3K activation is provided by cross-talk with the MAPK module; (b) insulin and TNFα could directly trigger the PI3K pathway, thus requiring activation of MAPK by cross-talk (Fig. 6D); (c) the MAPK and the PI3K pathways could be individually induced by insulin and TNFα making the cross-talk between PI3K and MAPK superfluous (Fig. 6E); (d) one of the two pathways could be activated via an ‘AND’ gate requiring both ligands, whereas the other is directly activated by only one of them. Here, the cross-talk had to be absent to prevent activation of PI3K and MAPK if only one ligand was present (Fig. 6F–H). The case in which the MAPK pathway is triggered by either TNFα or insulin is not a valid option, because it would activate PI3K through the cross-talk that enables IL-6 to induce DNA synthesis.

For the following analyses we implemented version v3 (Fig. 6E) that conserved all original cross-talks and required only few changes in the network structure, specifically the addition of two ‘AND’ gates for coactivation of the MAPK and PI3K modules to allow full reproduction of the experimental data (Fig. 6I). All other introduced model versions (Fig. 6F–H) would have been valid to achieve accordance with the DNA synthesis data as well, but would have required more modifications.

Using this refined integrated logical model we again compared the selected hepatocyte-specific literature scenarios (Doc. S3) with the corresponding model predictions. Agreement was further increased to 76.3%, while only 23.7% of the scenarios differed (Fig. 3, right). These values indicated a substantial improvement of our integrated logical model.

### Logical input hierarchy on the induction of DNA synthesis

For analysis of the input (presence of ligands)–output (DNA synthesis) behavior, we used the refined integrated logical model and extracted an input hierarchy regarding the ability to induce DNA synthesis. We extrapolated our experimental data by computing the outcome of all possible input combinations and extracted canalizing inputs from the resulting matrix (Fig. S4). A canalizing input determines the output of a system independently of other inputs [48]. In our model, only TGFβ met this criterion and was hence a canalizing input of the first order (or strong canalizing input). IL-6, HGF, EGF (or TGFα) and the combination insulin ‘AND’ TNFα were canalizing inputs of the second order (or weak canalizing inputs) because their activity induced DNA synthesis only when the strong canalizing input TGFβ was inactive. This result was translated into the following logical relationship that is schematically depicted in Fig. 6J (see Materials and methods for logical operators):


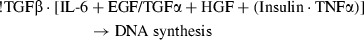


This input hierarchy allows TGFβ to have priority over any growth signal and to block hepatocyte proliferation even if mitogens are present.

### Structural sensitivity analysis identifies structural redundancy of the network

To assess the importance of structural errors, a sensitivity analysis was performed. The information best known about a protein–protein interaction is usually the immediate influence of one species on the other, whereas the complex interplay of several effectors and thus the model structure often remains uncertain. This is especially true if reactions are not ubiquitous and occur only in specific cell types. For our analysis, we removed or substituted one to three model reactions with time scale ‘1’ at a time and subsequently simulated the outcome for the scenarios of the literature as well as experimental dataset. The correct data simulation was judged relative to the original model, which was set to 100%. The result of the structural sensitivity analysis is shown in Table S6.1. In a pool of networks in which one reaction was removed, or an ‘AND’ gate was converted into an ITT or ‘OR’ gate, more than 83.3% of the models were able to yield a relative data reproduction of 90%. In the case of two affected reactions, this proportion decreased to 69.0%, and for 3 ‘AND’ gates converted into an ITT or ‘OR’ gate to 67.4%. However, the average of relative agreement between the new models and the reference structure for a model pool was higher than 91.0% for all investigated structural changes.

These results illustrated structural redundancy providing a rather stable predictive behavior to the network. For some structural changes even a slight increase in the relative agreement between the original and new model could be observed because the original model did not correctly reproduce the entire literature dataset. This finding indicated that an automated structure optimization could, in principle, improve the model’s predictive power further. There exists at least one model that can only reproduce as low as 27% of the predictions made by the original model. Indeed, the removal of certain reactions prevents any prediction for DNA synthesis because of a sinkhole structure present in the model that is constituted by several inputs affecting a single output.

### Analysis of positive feedback loops identifies species essential for restriction point bistability

Feedback loops are an essential regulatory structure. During the progression from G_1_ into S phase, hepatocytes reach independence from mitogenic stimuli (Fig. 1), and Yao *et al.* [27] showed that this restriction point is based upon a bistable Rb–E2F switch. Because bistability requires the presence of an extrinsic or intrinsic positive feedback loop [49–52], we computed the species participation in the positive loops of the interaction graph underlying our logical model to identify important players in the decision-making process (Materials and methods).

In our case, a feedback loop is an elementary cycle as defined in graph theory and represents a subnetwork along which a species can influence itself without visiting the same node, except the starting node, twice. It is distinct from a feedback, which is defined as an edge starting from a species and pointing back to one of its predecessors upstream in regulation. Because of these definitions a feedback can give rise to several feedback loops.

Computing the feedback loops in a network as large as the integrated model presented here proves challenging because of their great abundance. Hence, we focused on the ‘Cell Cycle (G_1_/S)’ layer, which already gives rise to a total of 6206 positive feedback loops. The nuclear form of the CDK inhibitors p21 and p27, the activated CDKs 2, 4 and 6, Rb as well as E2F1-3a proved to be part of most of the positive feedback loops in this layer (Fig. S5). These species are thus likely to be of major importance in restriction point control.

### MIS analysis can be used to predict mutations with malignant consequences

To predict mutations that could lead to liver cancer, we computed MIS of size ‘1’ to ‘4’ that led to induction of DNA synthesis in the absence of stimulation in our model, mimicking liver malignancies (Fig. S4). The validity of these predictions was tested by comparison with mutations of malignant liver tissue samples recorded in the COSMIC database (http://www.sanger.ac.uk/genetics/CGP/cosmic/, 3 April 2011).

The fact that a given gene is mutated does not *per se* provide information on functional consequences, but can identify candidates for deregulation in cancer cells. The comparison of MIS results and database allowed confirmation of 12 predictions in total (six true positives, six true negatives), whereas eight others were invalidated (two false positives, six false negatives) (Table 2). There was no database information available for the remaining species. Although our model suggested Akt1 and MYC as potential oncogenes, these targets were not recorded in the COSMIC database. By contrast, Raf, inhibitor of CDK4 (Ink4a), phosphatase and tensin homolog (PTEN), ARF, ATR and SMAD appear in the mutation database, but not in our analysis. These species might occur in MIS with larger cardinality (> 4), but their computation becomes difficult because of the increasing combinatorial complexity.

**Table 2:** Comparison of MIS leading to DNA synthesis in unstimulated cells and content of the COSMIC database. Comparison between targets found in MIS of size ‘1’ to ‘4’ leading to DNA synthesis in unstimulated cells, and data from mutations found in liver cancer cells extracted from the COSMIC database (http://www.sanger.ac.uk/genetics/CGP/cosmic/, status at 3 April 2011). Species names are written in italics. Corresponding genes from the COSMIC database are indicated in parentheses. According to a one-tailed Fisher exact test, the overlap between model predictions and database is substantial (*P* = 0.0513) if cell cycle inhibitors with basal activity, which are not considered in our model, are removed from the list

	Part of MIS	Not part of MIS
Mutation found in COSMIC	*pi3k* (pik3ca), *c_met* (met), *prb* (rb1), *egfr* (egfr), *jak* (jak1, jak2), *p53* (tp53)	*pten* (PTEN), *raf* (raf1), *arf* (cdkn2a (p14)), *smad* (smad4), *atm_atr* (atr), *ink4a* (cdkn2a)
No mutation found in COSMIC	*akt* (akt1), *c_myctrans* (myc)	*apc_cdh1* (cdh1), *ink4c* (cdkn2c), *c_ebp_alpha* (cebpa), *mkk4_7* (map2k4), *tsc* (tsc1/tsc2), *fbw7* (fbxw7)

Using a one-tailed Fisher’s exact test, we investigated whether a significant proportion of species identified by our MIS analysis overlapped with the COSMIC database. When considering all predicted species, the test yielded a *P*-value of 0.2596. However, because our model does not account for the basal activity of cell-cycle inhibitors required for blocking DNA synthesis in the absence of external stimuli, the species Ink4a, Ink4c, PTEN, ARF, ATR and SMAD were removed from the comparison, thus decreasing the *P*-value to 0.0513. This result showed that MIS analysis is a useful tool to predict mutations with malignant consequences.

### MIS analysis reveals targets to block HGF-stimulated DNA synthesis in hepatocytes

To obtain a list of possible single constitutive activations or inhibitions that led to cell-cycle arrest in proliferating cells, we computed the MIS of size ‘1’ that block DNA synthesis in the context of HGF stimulation. The result of this analysis is depicted inx Fig. S6 showing that 28 single species’ inhibitions and 32 single species’ activations would induce this event. Interestingly, four species with multivalued logic (*p53*, *e2f1_3a*, *p21nuc* and *p27nuc*) could be either completely activated (level ‘2’ or ‘3’) or inhibited (level ‘0’), reflecting their dual effect on proliferation.

Our previous model analysis had indicated a requirement for coactivation of the PI3K and MAPK pathways to allow induction of DNA synthesis upon mitogenic stimulation. This hypothesis was experimentally validated by targeting Akt1/2, PI3K and MEK1/2 with small molecule inhibitors (Figs 2 and 5A). In line with this result, these species were also identified as targets by our MIS analysis. To further verify our predictions, we selected three other intervention targets from different locations within the model to confirm their necessity for the induction of DNA synthesis in mitogen-stimulated cells (Fig. 2). For experimental validation we targeted the phosphoinositide-dependent protein kinase (PDK)1 in the PI3K module of the layer ‘Signaling Cascades’, the exporter chromosome maintenance region 1 or exportin 1 (CRM1) in the ‘Cell Cycle (G_1_/S)’ section, and the cyclin-dependent kinases (CDK)4/6 in the same model layer with the small molecule inhibitors BX912, leptomycin B (LMB) and PD0332991, respectively. As shown in Figs 5B,C and S7, the inhibitors dramatically reduced DNA synthesis in HGF-stimulated cells. BX912 and LMB mediated a complete block of DNA synthesis (Figs 5B,C and S7), whereas PD0332991 yielded a reduction of only ∼ 50% (Fig. 5B). This reduced effect was most likely attributed to metabolization by or export from the hepatocyte, as very high PD0332991 concentrations blocked DNA synthesis in a dose–response experiment (Fig. S7C). Our results for inhibition of Akt1/2, PI3K, MEK1/2, PDK1 and CRM1 show that our integrated logical model could identify intervention targets that mediate cell-cycle arrest in proliferating hepatocytes.

## Discussion

**Fig. 7:**
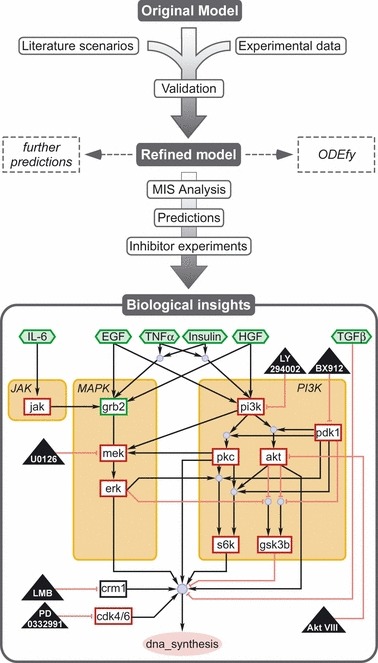
Biological insights obtained by combining logical modeling and experimental data. The original model was refined after validation using literature scenarios (Doc. S3) as well as our own experimental DNA synthesis data (Fig. 4A). Analysis of this model with help of LSS analysis and MIS computation demonstrated the requirement of (a) PI3K and MAPK pathway activation as well as (b) five key species within those pathways for inducing DNA synthesis. Predictions for species whose inhibition blocks HGF-mediated DNA synthesis were verified experimentally employing inhibitors against Akt, PI3K, MEK, CRM1, CDK4/6 or PDK1. The obtained validated model can be used in future applications, e.g. for predicting specific scenarios or as a starting point for the automatic setup of qualitative ODE models using the odefy software.

This study connects the influence of multiple pro- and antiproliferative signals to the mitogen-dependent G_1_ phase of the mammalian cell cycle in a single, large-scale integrated logical model and thereby provides a broad picture of proliferation control in primary hepatocytes. The employed workflow of model validation and analysis, the key results as well as the prospective use of the validated model are summarized in Fig. 7.

Mathematical modeling approaches allow investigation of the system’s properties. Dynamical models based on ordinary differential equations (ODE) or partial differential equations can describe systems dynamics in the most accurate way, but to enable analysis of the network’s time-dependent behavior their parameters, e.g. initial protein concentrations and kinetic rate constants, have to be fitted using time-resolved quantitative experimental data. With increasing network size this task becomes more and more challenging, in experimental as well as computational aspects. Thus, current ODE-based models are limited to a few dozen components insufficient to describe large systems [38]. Logical modeling based on the study of qualitative input–output relationships and structural network analysis, by contrast, solely depends on the model structure, and not on quantitative kinetic parameters, thus enabling functional investigation of large molecular networks.

To qualitatively judge the predictive power of our integrated logical model we compared distinct model scenarios with carefully selected hepatocyte-specific literature information. This approach was feasible, because a logical model does not depend on kinetic parameters and hence allows the direct use of literature information. By manually introducing specific modifications to the network, we successively increased the correlation between literature and model scenarios, and hence the model’s predictive power from initially 60.0% to 76.3%. Further model improvement by addition or removal of edges is conceivable in theory, and would require systematically fitting the model structure to experimental data, e.g. using cellnetoptimizer [12]. To allow such a process for large-scale models two procedures can be applied: (a) The model has to be drastically reduced to comprise only observed species. This minimizes the combinatorial complexity crucial for the fitting process, but such a reduction counteracts the construction of an utmost comprehensive model structure; and (b) high-throughput data of adequate quality for a large set of species have to be generated [12,14]. However, the number of hepatocytes isolated from mouse liver is limited and thus does not allow such extensive measurements at present. Because of these limitations further model refinement was not pursued.

The remaining percentage of false predictions for the comparison of literature and model scenarios could partially be attributed to the scarcity and heterogeneity of the literature-derived dataset, which encompassed only 16% of all model species excluding dummies and reservoirs. This underrepresentation gave great weight to any wrong prediction, and prevented a precise validation of the model structure including time scales. Furthermore, the hepatocyte-specific literature scenarios used for comparison were derived from various studies conducted under different experimental conditions. This can cause several problems: (a) Data can be contradictory. In a pair of supposedly identical scenarios in which DNA synthesis is observed in only one, the corresponding model prediction will be necessarily wrong in one case. For example, although insulin did not significantly trigger DNA synthesis in our experiments, Kimura *et al.* [53] found the opposite outcome, and whereas in our case DNA synthesis was significantly induced by EGF, Rickheim *et al.* [54] observed only a weak induction. (b) For comparison with binary model predictions, qualitative data derived from publications has to be translated into a ‘yes/no’ response [12]. It may be difficult to judge whether a species can be regarded as active when evaluating a literature-derived measurement. This is especially true if information on the possible range of the reported response in the experimental setting used is scarce or missing completely. These problems complicated the comparison of literature data with our model and emphasize the need for standardized experimental protocols [55].

The input–output behavior of the improved model was experimentally validated using primary mouse hepatocyte data of DNA synthesis in response to different stimuli. To enable comparison with binary model predictions, continuous experimental data has to be discretized, and different strategies of data processing are conceivable. Saez-Rodriguez *et al.* [12,56], for example, employed a sophisticated transformation strategy to interpret time course data for logical modeling purposes. In our approach, we used DNA synthesis data acquired at a single, defined time point and employed a statistical mixed linear model for data discretization. A significant induction of DNA synthesis compared with a control treatment was interpreted as a binary state of ‘1’. Likewise, the absence of significant induction was valued as ‘0’. It is important to note that the employed statistical test solely identifies a significant induction in DNA synthesis, but not its significant absence. Thus, it cannot be excluded that a treatment like insulin that failed to trigger statistically significant DNA replication in our experiments, may be capable of doing so in a different system [53].

The model predicted that all inputs except TGFβ could induce DNA replication. TNFα and insulin alone did not fulfill this prediction, because they had to be applied in combination to trigger a significant increase in DNA replication in our experiments, which was small compared with strong mitogens like HGF. In line with this observation, the mitogenic abilities of TNFα and insulin are known to be moderate, although they can enhance the effects of growth factors [57]. Our improved model furthermore predicted that TGFβ would prevent replication of the genetic information alone or as part of any input combination. Accordingly, TGFβ blocked DNA synthesis in the presence of HGF or HGF and insulin in our experiments. The model’s input hierarchy showed that this strong canalizing input had priority over any competing mitogenic signal. This dominance is a key feature of the TGFβ-induced cytostatic effect [58], which could be essential to tightly regulate the hepatocyte’s response to pro-proliferative signals. In our experiments, treatment with TGFβ alone led to a negative fold change in DNA content. This might be due to the notion that in addition to mediating cell-cycle arrest, TGFβ can also elicit pro-apoptotic effects [58]. Indeed, a small wave of hepatocyte apoptosis during the late phase of liver regeneration following two-thirds partial hepatectomy contributes to the precise re-establishment of the original liver mass [59].

The ligand concentrations used for hepatocyte stimulation are critical parameters of cellular responses, and we chose values known to induce robust pathway activation for our experiments [46]. *In vivo*, however, bioavailability and concentration depend on the location in the liver and change over time [60]. Particularly for IL-6, its precise level seems critical for its ability to induce proliferation [2,61]. Because logical modeling uses the qualitative system’s steady state to evaluate the network behavior, our model does not employ time-dependent input functions. Our approach thus elucidates, independent of the concentration, whether a distinct ligand is in principle capable of inducing DNA synthesis via the presented model structure. To investigate the impact of ligand concentrations, the logical interactions can be translated into an ODE system using the *ODEfy* plugin [62]. It might, however, be difficult to estimate the large number of parameters resulting from this conversion, and thus to perform dynamical analyses. Still, the logical model can be used to identify smaller subnetwork structures essential in mediating a ligand’s network response, which are more suitable for ODE-based analysis. Moreover, direct conversion into ODE systems using *ODEfy* allows reuse of the established Boolean network structure and renders time scales superfluous.

Although a large amount of published data was included in our model, such an interlaced network is prone to errors and missing edges. To assess this structural uncertainty, we randomly substituted or removed one to three interactions and observed that the resulting models could reproduce on average > 91% of the predictions made by the original network. This result illustrates redundancy in our network, which could confer a relative robustness to perturbations ensuring survival upon liver damage. In line with this hypothesis, mouse models featuring distinct mutations fail to show a complete block of hepatocyte proliferation when subjected to two-thirds partial hepatectomy, but rather display a decelerated regenerative response [2]. The interaction map underlying our model contains a detailed description of the G_1_/S transition and simplified representations of important signaling cascades that regulate it. However, uncertainties concerning the wiring of these two entities remain. Based on our interaction map, future work could include connecting the cell-cycle layer to more detailed, experimentally validated logical networks of signaling pathways to analyze activation patterns upon successive stimulation with different ligands, as well as the specific contribution of distinct signaling cascades. For example, the impact of TNFα and IL-6, which do not seem to be strictly required for liver regeneration, could be investigated.

We identified candidate nodes involved in inducing the bistable behavior required to cross the restriction point, and hence gain mitogenic independence. Bistability requires the presence of extrinsic or intrinsic positive feedback loops [49–52]. The model layer ‘Cell Cycle (G_1_/S)’ contained a great number of such loops and by computing the species’ participation in them, we demonstrated that Rb, cyclin D : CDK4/6, cyclin E : CDK2, as well as the nuclear forms of the CDK inhibitors p21 and p27 are of major importance in restriction point and proliferation control. In line with this result is the notion that the CDK4–cyclin D1–INK4–Rb-E2F cascade is deregulated in > 80% of human malignancies [63]. Although CDKs are rarely mutated, deregulation of CDK4/6 has been described in various tumors, e.g. in melanoma [63,64]. In addition, overexpression of cyclin D1 is a frequent feature of neoplasias [63,65], and serves as a prognostic marker in pancreatic cancer [66]. Inhibition of Rb function contributes to cancer initiation and progression [67,68], and loss of Rb is involved in the development of liver cancer and other neoplasias [68]. Overexpression of cyclin E1 is a frequent feature and indicator for poor prognosis in a number of malignancies [66,69–71]. Although somatic mutations of p21 are rare, loss of p27 is a more abundant feature in cancer [66,72]. In addition, the presence of unnatural cytoplasmic p21 and p27 unable to inhibit nuclear CDK activity indicates poor prognosis in a variety of tumors including breast cancer [73].

A comparison of oncogenic modifications predicted by our model and the COSMIC database showed substantial agreement. Targets suggested by the model, but not present in the database, were Akt1 and Myc. Although no mutation of Akt1 has been described to date, it is a central component of the PI3K signaling pathway that is frequently altered in hepatocellular carcinoma [74]. Likewise, deregulation if not mutation of the transcription factor Myc was found to induce aggressive liver cancer [75]. Although Raf, Ink4a, PTEN, ARF, ATR and SMAD appear in the mutation database, they were not part of our MIS prediction. In our network, Raf1 did not induce PI3K pathway activation and was hence not identified by our analysis. It might, however, appear in MIS of larger cardinality. In our model, mitogenic stimulation is required to induce DNA synthesis and accordingly, the inhibitors of the cell cycle Ink4a and PTEN were set to ‘0’ to allow mitogenic signal propagation. *In vivo* however, their presence is required to prevent cell division induced by the basal activity of mitogenic pathways [66,76,77], explaining the discrepancy between our predictions and the database. The tumor suppressors ARF and ATR, as well as the transcription factor SMAD, mediate a cytostatic response upon DNA damage [78] or TGFβ signaling [79]. Deregulation of these species may hence lead to uncontrolled proliferation of damaged cells.

Through model analysis we furthermore predicted that coactivation of the PI3K and MAPK pathways is sufficient and required for induction of DNA synthesis. The latter hypothesis was experimentally validated in primary mouse hepatocytes using inhibitors targeting Akt1/2, PI3K and MEK1/2 kinases. Our results are in line with the work of Heo *et al.* [80] who demonstrated the requirement for the PI3K and MAPK pathways in the regulation of DNA synthesis by TGFα in mouse embryonic stem cells using the same intervention strategies. Furthermore, inhibition of Akt1/2 and PI3K with Akt inhibitor VIII and LY294002, respectively, sensitizes hepatocellular carcinoma-derived cells for apoptotic cell death, which indicates that Akt1/2 and PI3K are prone targets for anticancer therapies [81]. Inhibition of MEK1/2 by U0126 was also shown to mediate cell-cycle arrest and apoptosis in hepatocellular carcinoma cell lines [82].

We lastly employed MIS analysis to propose species whose activation or inhibition prevents induction of DNA synthesis in the presence of the strong mitogen HGF. The large number of possible MIS of size ‘1’ might seem to contradict the relative robustness identified through our sensitivity analysis at first glance. However, although this approach only modified one specific downstream function of a protein at once, MIS constitutively affect all functions of a specific protein at the same time. To experimentally validate our model predictions, selective perturbations were necessary at the population level. Transfection of primary hepatocytes for overexpression or siRNA-based knockdown rarely exceeds 45% efficiency and is therefore insufficient to achieve this goal [83,84]. In line with our results concerning the requirement for coactivation of the PI3K and MAPK pathways, the MIS analysis also identified Akt, PI3K and MEK as potential intervention targets. We employed further small molecule inhibitors to validate the three additional MIS targets PDK1, CRM1 and CDK4/6. Inhibition of PDK1 kinase activity or CRM1 transport function by BX912 [85,86] or LMB [87,88] respectively, led to complete inhibition of DNA synthesis in the presence of HGF, thus confirming our MIS result. In line with our model prediction and experimental observation, inhibition of CRM1 by LMB has been reported to result in G_1_ and G_2_ cell-cycle arrest of mammalian cells and fission yeast [89]. Inhibition of CDK4/6 kinase activity by PD0332991 [90] only resulted in attenuated DNA replication, probably due to metabolic conversion of the compound reducing its efficacy. Although it has shown promising performance in preclinical studies for the treatment of cancer [91,92], very high concentrations, often equivalent to the maximum tolerated dose, were necessary to obtain an antiproliferative effect in xenograft models [92,93]. This suggests that high PD0332991 doses are required to compensate for metabolic clearance and underscore the problem of strong metabolic activity in the treatment of hepatocellular carcinoma.

By employing model-based MIS analysis and DNA synthesis assays in primary mouse hepatocytes, we theoretically predicted and experimentally validated several targets in our complex network critical in mediating HGF-driven proliferative responses. Our results show that MIS analysis of a large integrated logical model following experimental validation is a powerful tool to understand complex biological processes and to predict intervention targets (Fig. 7) to counteract undesired proliferation like in the context of hepatocellular carcinoma.

## Material and methods

### Logical modeling

The model presented here is based on the logical modeling framework of logical interaction hypergraph (LIH) [41] which is directly supported by the software tools promot [44] and cellnetanalyzer [40]. In contrast to studies focusing on the discrete dynamics of logical networks [21,22,25,31], the formalism is tailored to study the qualitative input–output response of signaling networks. Nodes in the network represent biomolecular species, e.g. kinases, transcription factors or genes, each having an associated logical state, which is often Boolean (‘on’/’1’ or ‘off ’/’0’), but multiple discrete levels are allowed as well. In the LIH representation, signaling events are encoded as Boolean or logical operations on the network nodes using only the operators ‘AND’ (·), ‘OR’ (+) and ‘NOT’ (!). They are sufficient to represent any logical relationship and are combined in a so-called sum-of-product representation, where ‘AND’ terms are connected via ‘OR’ operators [40,41]. To exemplify this we assume that the nodes *A* and *B* must be active to activate a third node *C*: *A AND B → C* or, shorter, *A · B → C*. In a graphical representation of the network such an ‘AND’ connection can be displayed as a hyperarc indicating that all start nodes of the hyperarc (*A*, *B*) must be in the ‘on’ state in order to activate the end node of the hyperarc (note that in contrast to simple arcs, hyperarcs are allowed to have several start or end nodes). In Fig. 6A, for example, there is a hyperarc indicating that *mtor*‘AND’*pkc*‘AND’*pdk1* are required to activate *s6k*. The hyperarc consists of a blue circle having a set of inputs which will be combined via ‘AND’s and one output node. Generally, it is also allowed that a hyperarc is a simple edge, i.e. it has only one input node. Here we use ‘interactions’ or ‘reactions’ as synonyms for hyperarcs.

‘NOT’ operators can be applied to variables entering a hyperarc and are graphically indicated by red color and bars. They indicate that a given input must be inactive for the output to be active. Finally, ‘OR’ connections can be accounted for in the hypergraphical representation by allowing a node to be independently activated by more than one incoming hyperarc (i.e. by several independent ‘AND’ connections). For example, there are two different hyperarcs for the activation of *mtor*. In this way, a hypergraph can effectively display the SOP representation. In some cases it is useful to introduce ‘dummy’ nodes, which do not correspond to concrete biological species but may simplify editing large networks. For example, a Boolean equation *X* = (*A* + *B* + *C*) · (*D* + *E* + *F*) can be represented via two dummy nodes *d1* = *A* + *B* + *C* and *d2* = *D* + *E* + *F* which are then combined in a hyperarc (‘AND’ connection) pointing into X (X = *d1·d2*). This representation is simpler than using the explicit SOP representation which would contain nine ‘AND’ terms. Furthermore, some species are defined as reservoirs and characterize a pool of a specific protein that is part of another species, e.g. because it can bind several partners. Thus, setting the reservoir to ‘0’ leads to the inhibition of all complexes that contain this species.

Whereas interaction graphs describe positive or negative influences between species, the LIH integrates the relationship between several regulatory predecessors of the same species. For example, if *A* and *B* are activators of *C* in the interaction graph, the associated logical representation would depend on whether *A* or *B* can activate *C* alone (‘OR’), or whether both (‘AND’) are required. If this information is not available an incomplete truth table (ITT) gate can be used to implement the case where the presence of only one input results in an unknown output. This limits the determinacy of the logical steady state, but provides more reliable results.

Embedded in the LIH formalism, cellnetanalyzer also supports multivalued logic to mimic the fact that, in reality, multiple relevant threshold values or states for a species may exist. In addition to the binary on/off case, other levels for the species can be defined. For example we can formulate a logical function *!A* · 2*B →* 3*C*, expressing that *C* reaches level ‘3’ if *A* is inactive (level ‘0’) ‘AND’*B* is at level ‘2’.

The hypergraphical representation facilitates the analysis of a number of useful properties of a logical network or its underlying interaction graph [11,13–15]. Here, we use our model for computing feedback loops, MIS (see below) and for predicting the qualitative input–output behavior. This is achieved by propagating input signals and perturbations, e.g. knockout of nodes, along the logical hyperarc connections to compute the resulting LSS [43], which reflects the final qualitative response of the network, though not the temporary sequence of signal flows.

### Computation of feedback loops

Computing the feedback loops of a logical network in a LIH representation requires its conversion into an interaction graph by splitting all ‘AND’ gates. Duplicated edges arising from this process are removed before counting the actual number of cycles. For example, for the following multilevel network with two hyperarcs, i.e. ‘AND’ connections, pointing to node *C*: *{A* · *!D = C*; *!D* · *2B = 2C}*, the corresponding interaction graph is represented by the following signed edges: *A → C*, *B → C*, *D −|C*.

### Initial response

In some cases, the presence of feedback loops may prevent the computation of a unique LSS for some or all species. Indeed negative feedback loops might generate oscillations, whereas positive feedback loops can induce multistationarity. One possibility to cope with this problem is to focus on the initial response of the network by introducing time scales. Typically, feedback loops become active only after some time once a signaling event has been triggered. For each feedback loop, we may therefore declare the cycle-closing edge to a higher time scale, i.e. it will be set to ‘0’ when considering the initial response. The obtained LSS then reflects the initial response of the nodes in the network, which is of main interest in most applications.

A typical example is represented by the MAPK cascade as modeled by Kholodenko *et al.* [94] and others. Figure S8A shows the activation in this cascade and the negative feedback from the end kinase (extracellular signal-regulated kinase; ERK) to the input (rat sarcoma; Ras). Simulation without feedback leads to a sustained activation of ERK, whereas inclusion of this negative feedback generates sustained oscillations (Fig. S8B). The potential existence of oscillations prevents the determination of a LSS when translated into a logical structure (Fig. S8C). The signal must be able to propagate down to ERK at first in order to activate the feedback loop and for this to happen the loop can initially be considered to be open in the logical model by defining it at time scale ‘2’. The resulting two time scales do not define the two reactions as slow or fast, but rather specify their sequence of activation. Hence, they represent a local property of the activation of Raf. In order to adjust the time scale within which Raf can be inhibited, a dummy is incorporated, and a time scale of ‘2’ set for the reaction *ERK → dummy*.

Whereas the shape of ERK activation seems to determine cell fate, the outcomes of the different ERK activation modes are somehow unclear. Although it is commonly accepted that a sustained activation characterizes cell differentiation and a transient one cell proliferation, some authors showed that only a sustained ERK activation can induce cyclin D1 expression and cell-cycle entry [95]. Logical models represent a good approach to handle large networks, but dynamic modeling in conjunction with large amounts of quantitative experimental data will be required if the precise transient course and quantitative aspects of protein activation are of interest. This approach can be used with the help of the *ODEfy* plugin [62] which converts logical models into dynamical ODE models and was included to cellnetanalyzer.

In this study, the model prior to modifications contained 37 edges with a time scale of ‘2’ assigned from biological knowledge. Logical analyses were performed for time scale ‘1’ only (where time scale ‘2’ edges are thus temporarily removed from the network). Most, but not all, cycles were disrupted when removing the time scale ‘2’ edges. These remaining cycles can potentially prevent LSS computation in specific scenarios (main text).

### Minimal intervention sets

MIS are defined as irreducible (support-minimal) sets of constitutive activations and inhibitions of species that will enforce a desired network response [41,43]. For this purpose, a context (e.g. external inputs or knockouts) and an intervention goal (activation or inhibition of specific species) have to be defined. MIS are then computed by determining all combinations of constitutive activations and/or inhibitions that enforce an LSS where the intervention goal is satisfied in the given context. The algorithm was described by Samaga *et al.* [43].

### Sensitivity analysis

Because logical models do not depend on kinetic parameters, a sensitivity analysis investigates the effect of structural errors on the predictive power. Structural changes were obtained by removing one or more reactions of time scale ‘1’ as proposed in [13], or by substituting them by an OR or ITT gate. Following each model modification, the effects on the comparison of model predictions and data, i.e. literature as well as experimental datasets, were measured by counting the number of wrong predictions (Doc. S6). Because the scenario where EGF alone is the stimulus was present in both datasets with different outcomes, we chose to include only the result of our own dataset, i.e. EGF induces DNA synthesis. Both scenarios, where TNFα is used as a stimulus, were combined as they were not contradictory.

### Software

The logical models were constructed with the editing tool promot [44] and analyzed with the MATLAB package cellnetanalyzer [40] using its computational methods and application programming interface [96]. Dynamical simulations (Fig. S8B) were performed using the simbiology® toolbox of matlab® (MathWorks, Ismaning, Germany). The model is provided in promot format in the Supporting information. Furthermore, the cellnetanalyzer model files are available from the model repository of this software at the following website: http://www.mpi-magdeburg.mpg.de/projects/cna/repository.html.

### Network construction

The network was constructed by compiling information found in a total of 257 publications as listed in Doc. S2. It represents a master network for mammalian cells with information from different cell types.

### Chemicals

Unless stated otherwise, chemicals were purchased from Sigma-Aldrich (Munich, Germany).

### Isolation of primary mouse hepatocytes

Primary mouse hepatocytes were isolated according to a standardized procedure [97]. All mice were housed at the DKFZ animal facility under a constant light/dark cycle, maintained on a standard mouse diet, and allowed *ad libitum* access to food and water. All animal experiments were approved by the governmental review committee on animal care of the state Baden Württemberg, Germany (reference number A24/10). Briefly, 8–12-week-old male C57BL/6N mice (Charles River, Sulzfeld, Germany) were used for primary hepatocyte isolation. Anesthesia was carried out by intraperitoneal injection of 5 mg ketamine hydrochloride 10% (w/v) (Bayer Health Care, Leverkusen, Germany) per 100 mg body weight and 1 mg xylazine hydrochloride 2% (w/v) (Pfizer, Berlin, Germany) per 100 mg body weight. The liver was successively perfused with EGTA-containing buffer (0.6% w/v glucose, 105 mm NaCl, 2.4 mm KCl, 1.2 mm KH_2_PO_4_, 26 mm Hepes, 490 μm l-glutamine, 512 μm EGTA, 15% v/v amino acid solution, pH 8.3) and collagenase-containing buffer (0.6% w/v glucose, 105 mm NaCl, 2.3 mm KCl, 1.2 mm KH_2_PO_4_, 25 mm Hepes, 490 μm l-glutamine, 5.3 mm CaCl_2_, 12% v/v amino acid solution, 365 μg·mL^−1^ collagenase type 1-A, pH 8.3) in an anterograde fashion via the portal vein. The amino acid solution consisted of 270 mg·L^−1^ l-alanine, 140 mg·L^−1^l-aspartic acid, 400 mg·L^−1^ l-asparagine, 270 mg·L^−1^ l-citrulline, 140 mg·L^−1^ l-cysteine hydrochloride monohydrate, 1 g·L^−1^ l-histidine monohydrochloride monohydrate, 1 g·L^−1^ l-glutamic acid, 1 g·L^−1^ l-glycine, 400 mg·L^−1^ l-isoleucine, 800 mg·L^−1^ l-leucine, 1.3 g·L^−1^ l-lysine monohydrochloride, 550 mg·L^−1^ l-methionine, 650 mg·L^−1^l-ornithine monohydrochloride, 550 mg·L^−1^ l-phenylalanine, 550 mg·L^−1^ l-proline, 650 mg·L^−1^ l-serine, 1.35 g·L^−1^l-threonine, 650 mg·L^−1^ l-tryptophane, 550 mg·L^−1^ l-tyrosine and 800 mg·L^−1^ l-valine, pH 7.6. The liver was subsequently withdrawn and transferred into washing buffer (0.6% w/v glucose, 105 mm NaCl, 2.4 mm KCl, 1.2 mm KH_2_PO_4_, 26 mm Hepes, 1 mm CaCl_2_, 0.4 mm MgSO_4_, 0.2% w/v BSA, 15% v/v amino acid solution, pH 7.6). Hepatocytes were collected by disrupting the liver capsule and filtering the suspension through a 100 μm cell strainer (BD Biosciences, Heidelberg, Germany). Cells were washed twice by centrifugation at 50 ***g*** for 2 min. Cell yield and vitality were determined by Trypan Blue staining, and preparations exhibiting a vitality > 70% were used for our studies. For experiments, hepatocytes were seeded at subconfluency in full medium [phenol red-free Williams E medium (Biochrom, Berlin, Germany) supplemented with 10% v/v fetal bovine serum (Life Technologies, Darmstadt, Germany), 0.1 μm dexamethasone, 10 μg·mL^−1^ insulin, 2 mm l-glutamine and 1% (v/v) penicillin/streptomycin 100× (both Life Technologies)] using collagen I-coated cell ware (BD Biosciences) [Correction added on 10 May 2012 after original online publication: in the preceding sentence ‘1 μm dexamethasone’ was changed to ‘0.1 μm dexamethasone’]. Cells were cultured at 37 °C, 5% CO_2_ and 95% relative humidity. Following adhesion cells were washed with NaCl/P_i_ (PAN Biotech, Aidenbach, Germany) to remove unattached hepatocytes and subsequently cultured in serum-free cultivation medium (phenol red-free Williams E medium supplemented with 0.1 μm dexamethasone, 2 mm l-glutamine, and 1% v/v penicillin/streptomycin 100×) for 24 h prior to experiments [Correction added on 10 May 2012 after original online publication: in the preceding sentence ‘1 μm dexamethasone’ was changed to ‘0.1 μm dexamethasone’].

### Sybr® Green I assay

Cells were washed twice with NaCl/P_i_, received fresh serum-free cultivation medium and were stimulated with the following factors either alone or in combination for a total of 48 h: 40 ng·mL^−1^ recombinant mouse (rm) HGF, 20 ng·mL^−1^ rmTNFα, 1 ng·mL^−1^ recombinant human (rh) TGFβ (all R&D Systems, Wiesbaden, Germany), 50 ng·mL^−1^ rhEGF (Millipore, Molsheim, France), 100 nm insulin, 40 ng·mL^−1^ rhIL-6 (gift from S. Rose-John, Christian-Albrechts-University, Kiel, Germany). Factors were dissolved in cultivation medium or acidified double-distilled H_2_O (insulin). For inhibitor treatments, cells were preincubated with 10 μm Akt inhibitor VIII (Calbiochem EMD Millipore, Darmstadt, Germany), 10 μm LY294002, 10 μm U0126 (both Cell Signaling Technology, Frankfurt, Germany), 1 nm LMB (gift from M. Yoshida, RIKEN Advanced Science Institute, Saitama, Japan), 15 μm BX912 (Axon Medchem, Groningen, the Netherlands), 2 μm PD0332991 (Selleck Chemicals, Houston, TX, USA) or equal volumes of ethanol or dimethylsulfoxide for 30 min prior to addition of 40 ng·mL^−1^ HGF. LMB was dissolved in ethanol; Akt inhibitor VIII, LY294002, U0126, BX912 and PD0332991 were dissolved in ditheylsulfoxide. In each experiment, cells derived from a single animal were used and treatments were performed in technical triplicates. After the first 24 h, medium, stimulus and if applicable inhibitory treatment were renewed. At the end of cultivation, cells were washed twice with NaCl/P_i_ and frozen at –20 °C for at least 24 h. To assay DNA content, plates were incubated with 2 mL·well^−1^ of Sybr® Green I working solution (Sybr® Green I 10.000×) (Life Technologies, Darmstadt, Germany) diluted 1 : 2500 in NaCl/P_i_ supplemented with 0.1% v/v Triton X-100 (Roche Applied Sciences, Mannheim, Germany) for 1 h in the dark. Fluorescence intensity was read using an Ascent Fluoroscan plate reader (Thermo Fisher Scientific, Bonn, Germany) with λ_excitation_ = 485 nm and λ_emission_ = 538 nm.

### Statistical analysis

At least three individual experiments were used together for statistical analysis of the Sybr® Green I assay data, where each experiment was conducted in technical triplicate employing hepatocytes isolated from a single animal. To account for the two levels of variability, i.e. technical variability within an experiment and biological variability between different experiments, a mixed linear model (sas prox mixed, v. 0.2; SAS Institute Inc., Cary, NC, USA) was used with fixed factor stimulation condition and random intercept for the experiment. A detailed description of the use of mixed linear models for statistical analysis can be found in Littell *et al.* [47]. Prior to statistical analysis, data were scaled to the average of the experiment’s fluorescence intensity and log_2_ transformed. Dunnett contrasts were used to compare the different stimulation conditions with the respective control (unstimulated or solvent control). Results were plotted as log_2_ fold change with 95% confidence intervals. The null hypothesis was defined such that the induction of DNA synthesis by any treatment is not significantly different from the induction of DNA synthesis in the reference sample. The significance threshold for *P*-values was set to 5%.

To test the significance of the positive correlation between model predictions on mutations with oncogenic consequences and the COSMIC database, a one-tailed Fisher’s exact test was performed. The null hypothesis was defined as such that there is no significant overlap between the model predictions and the mutations found in the COSMIC database (http://www.sanger.ac.uk/genetics/CGP/cosmic/, 3 April 2011). The significance threshold for *P*-values was set to 5%.

## Abbreviations

Akt: protein kinase B
CDK: cyclin-dependent kinase
CRM1: chromosome maintenance region 1 or exportin 1
EGF: epidermal growth factor
ERK: extracellular signal-regulated kinase
HGF: hepatocyte growth factor
IL-6: interleukin-6
Ink4: inhibitor of CDK4
ITT: incomplete truth table
JAK: Janus kinase
LIH: logical interaction hypergraph
LMB: leptomycin B
LSS: logical steady state
MAPK: mitogen-activated protein kinase
MEK: MAPK kinase
MIS: minimal intervention set
ODE: ordinary differential equations
PDK1: phosphoinositide-dependent kinase 1
PI3K: phosphatidylinositol 3-kinase
PTEN: phosphatase and tensin homolog
Ras: rat sarcoma
Rb/pRb: retinoblastoma protein
SMAD: mothers against decapentaplegic homolog
TGF: transforming growth factor α
TGFβ: transforming growth factor β
TNFα: tumor necrosis factor α

## Supporting information

The following supplementary information is available:

**Doc. S1.** Figs S1–S8.

**Doc. S2.** Model documentation (reactions and species) and list summing up the changes applied to the original model.

**Doc. S3.** Documentation of the hepatocyte-specific scenarios used for the model validation.

**Doc. S4.** Description of the experimental setup for Western blot analysis of BX912 performance.

**Doc. S5.** *P*-values of the statistical analysis performed on the experimental data presented in Figs 4A and 6.

**Doc. S6.** Results of the structural sensitivity analysis.

**Appendix S1.** Supplementary_ProMoT_files.mdl contains the logical model in *ProMoT* format and the library of required logical elements.

This supplementary material can be found in the online version of this article.

Please note: As a service to our authors and readers, this journal provides supporting information supplied by the authors. Such materials are peer-reviewed and may be re-organized for online delivery, but are not copy-edited or typeset. Technical support issues arising from supporting information (other than missing files) should be addressed to the authors.
