# Supplementary material for: An integrative model links multiple inputs and signaling pathways to the onset of DNA synthesis in hepatocytes
Source: FEBS J. 2012 Sep;279(18):3290–313. doi: 10.1111/j.1742-4658.2012.08572.x (PMC3466406; doi:10.1111/j.1742-4658.2012.08572.x)
Supplement: Supplementary file 1 [file febs0279-3290-SD1.zip › febs_8572_sm_DocS1-S6.pdf]

the FEBS Journal
www.febsjournal.org

An integrative model links multiple inputs and signaling pathways to the onset of DNA synthesis in hepatocytes

Jérémy Huard, Stephanie Mueller, Ernst D. Gilles, Ursula Klingmüller and Steffen Klamt

DOI: 10.1111/j.1742-4658.2012.08572.x

Supplementary information

Huard J, Mueller S, Gilles ED, Klingmüller U & Klamt S (2012) An integrative model links multiple inputs and signaling pathways to the onset of DNA synthesis in hepatocytes.

Contents

Doc. S1 Supplementary figures **2**

Doc. S2 Documentation of the original model **10**

Doc. S3 Hepatocyte-specific scenarios for model validation **43**

Doc. S4 Western blot analysis of BX912 performance **48**

Doc. S5 Statistical analysis of experimental data - p-values **49**

Doc. S6 Results of the structural sensitivity analysis **50**

References **51**

Doc. S1 Supplementary figures

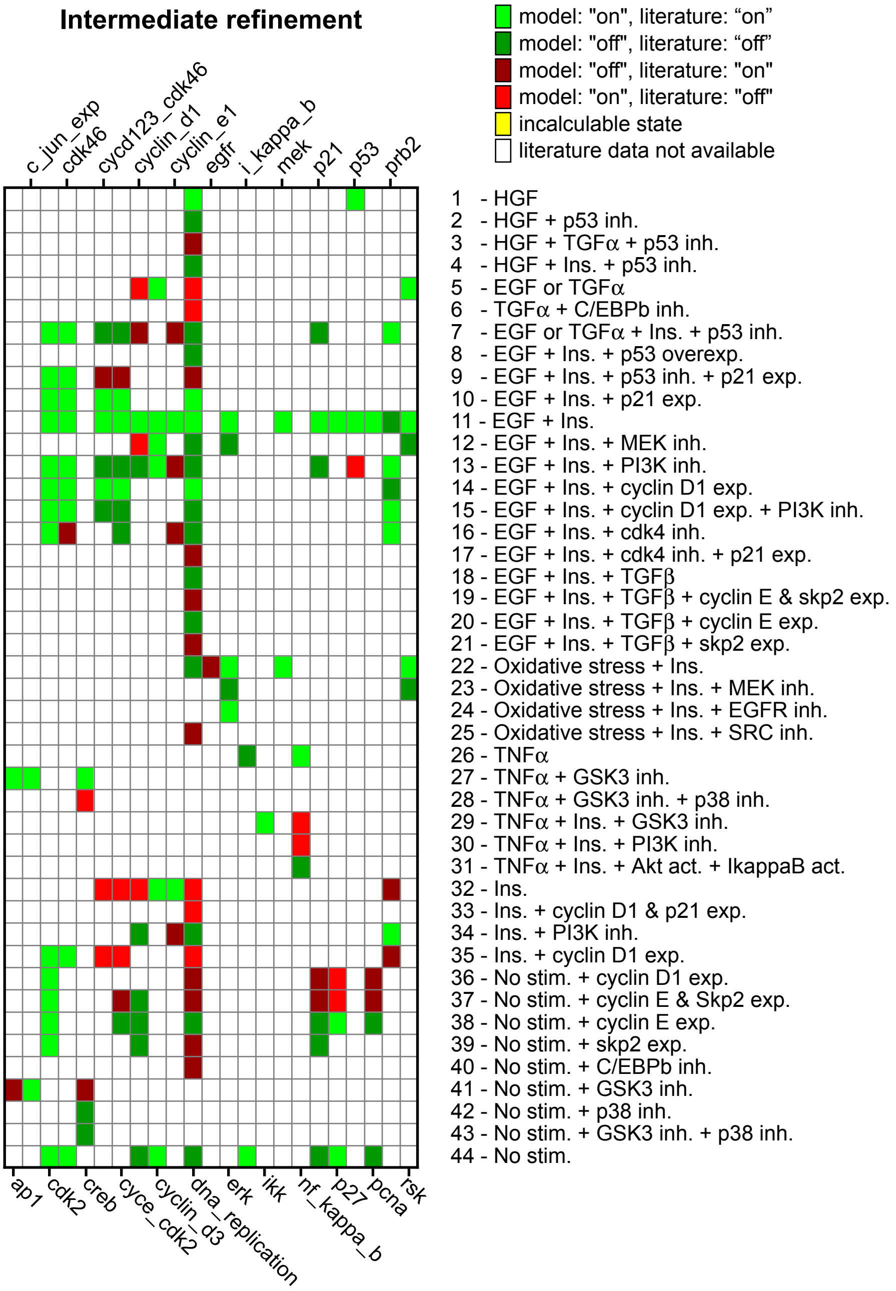

Fig. S1 – Matrix for comparison of model predictions and selected literature dataset after intermediate model refinement. A murine hepatocyte-specific literature dataset comprising 44 scenarios (vertical axis) and 23 species (horizontal axes) was selected and used for comparison with model predictions to qualitatively judge its predictive power. A detailed description of this dataset with all references can be found in Doc. S3. Concordance between model predictions and literature dataset is colorcoded (upper right corner). An intermediate state of model refinement led to an agreement between model predictions and literature dataset of 71.3 %. Divergence of scenarios occured in 28.7 % of the cases. No incalculable states remained. Abbreviations: act: activation, inh.: inhibition, Ins.: Insulin, overex.: overexpression, exp: expression, No stim.: no stimulation.

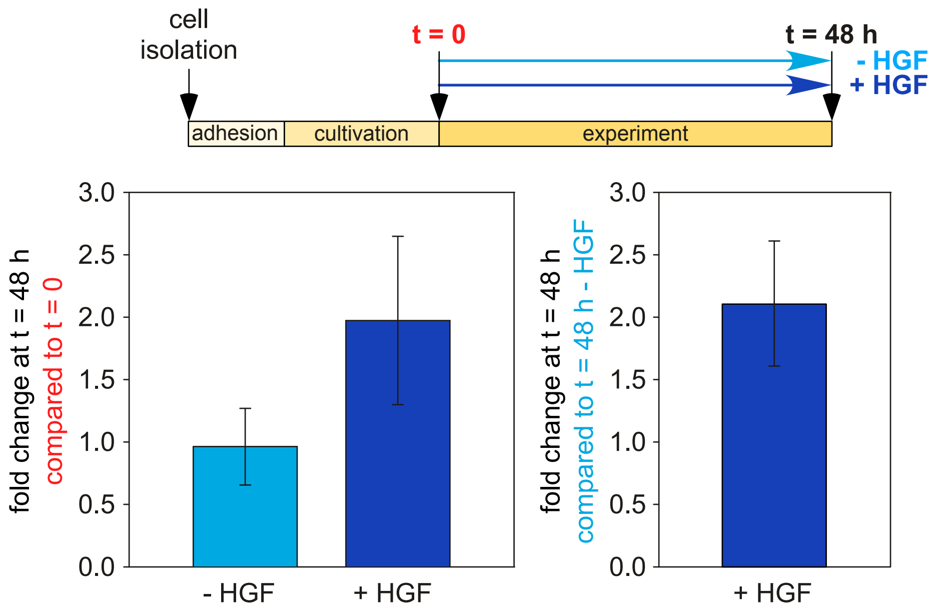

Fig. S2 – **DNA content of hepatocytes over time.** Primary mouse hepatocytes were cultivated under serum-free conditions and stimulated with 40 ng/ml HGF for 48h, or left untreated. DNA content was subsequently assayed using Sybr® Green I (Materials and Methods). Fold changes were calculated by comparison of cells collected at t=48h to unstimulated cells at either t=0 (left panel) or t=48h (right panel). The fold change for unstimulated cells was 0.96 $\pm$ 0.31, showing that the DNA content remained constant in the absence of stimulation over the experimental observation period. HGF-stimulated cells showed a fold change of 1.97 $\pm$ 0.67 (compared to t=0) and 2.11 $\pm$ 0.50 (compared to t=48 h) respectively, indicating a doubling of the genetic information upon HGF treatment. Data represent mean values of 21 experiments. Error bars indicate standard deviation. A schematic representation of the performed experiment is depicted above the data for clarification on time points used for analysis.

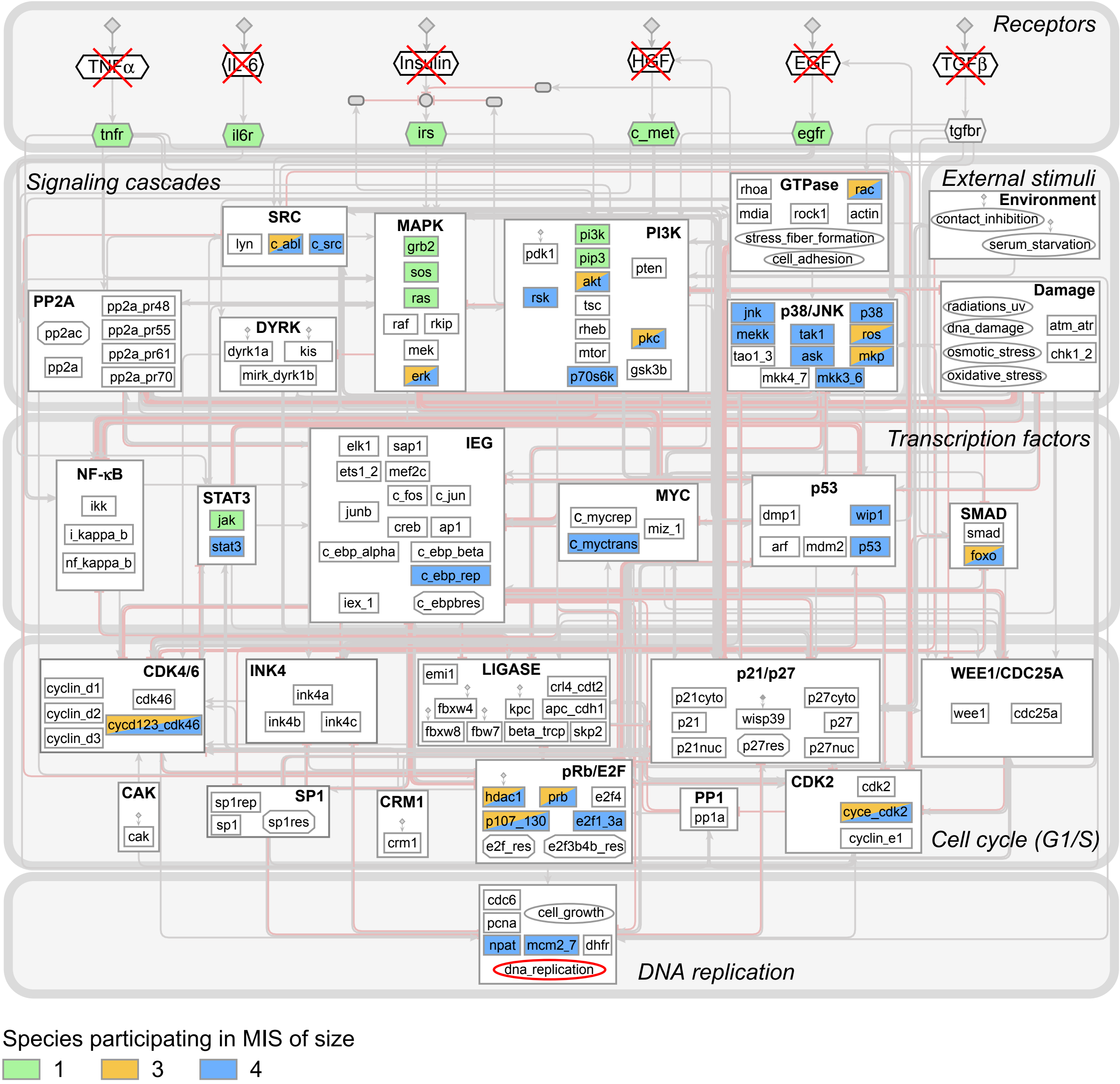

Fig. S3 – Species participation in MIS of size '1' to '4' leading to DNA synthesis in unstimulated cells. Minimal intervention sets (MIS) leading to the state *dna_replication* = '1' (red oval) when all inputs are set to '0' (indicated by red crosses) were computed. Species are color-coded according to the size of MIS they participate in (lower left corner) and depicted in the schematic model (see also Fig. 2). It should be noted that no MIS of size '2' could be identified, and that all MIS of size '3' are also part of MIS of size '4'.

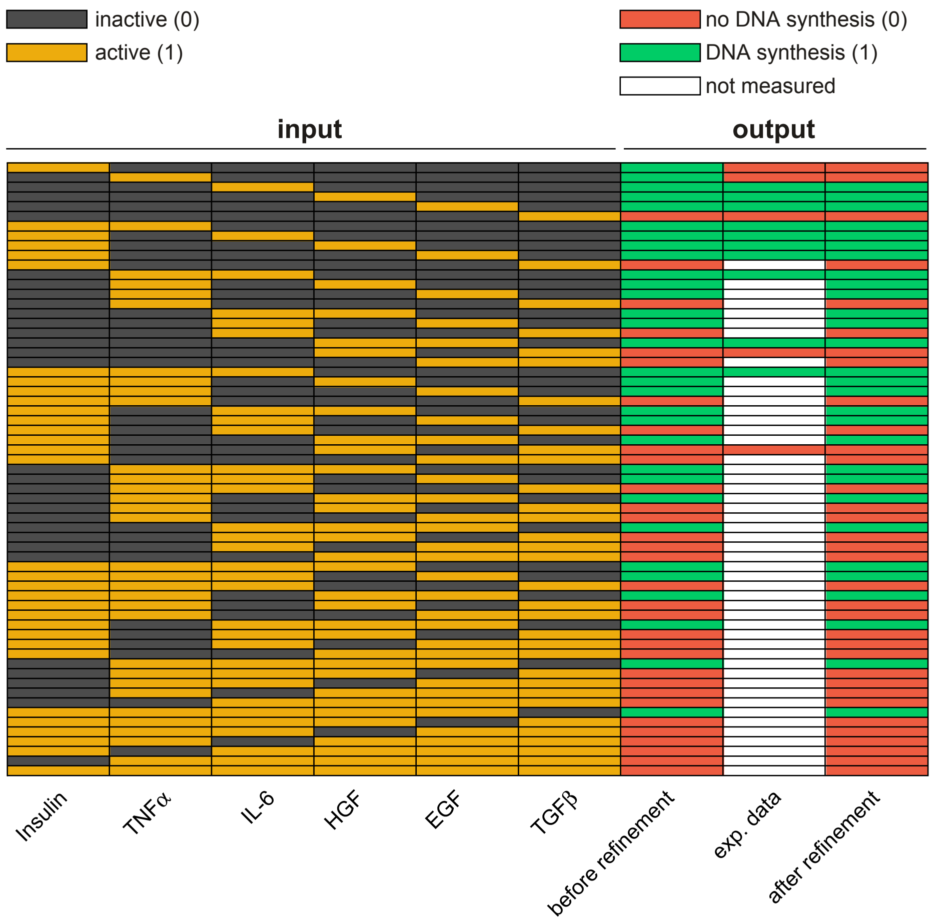

Fig. S4 – Matrix for comparison of model predictions for DNA synthesis of all possible input combinations before and after model refinement with experimental data. Each row represents the comparison of the model predicition for the output (DNA synthesis) before and after model refinement for a distinct input (ligand) combination with the experimental DNA synthesis data obtained in primary mouse hepatocytes (see also Fig. 4A). Inputs in orange are present (active or '1'), while grey ones are absent (inactive or '0'). A green output indicates induction of DNA synthesis, while red outputs equal absence of DNA replication. White fields indicate that no experimental data was generated for the corresponding input combination.

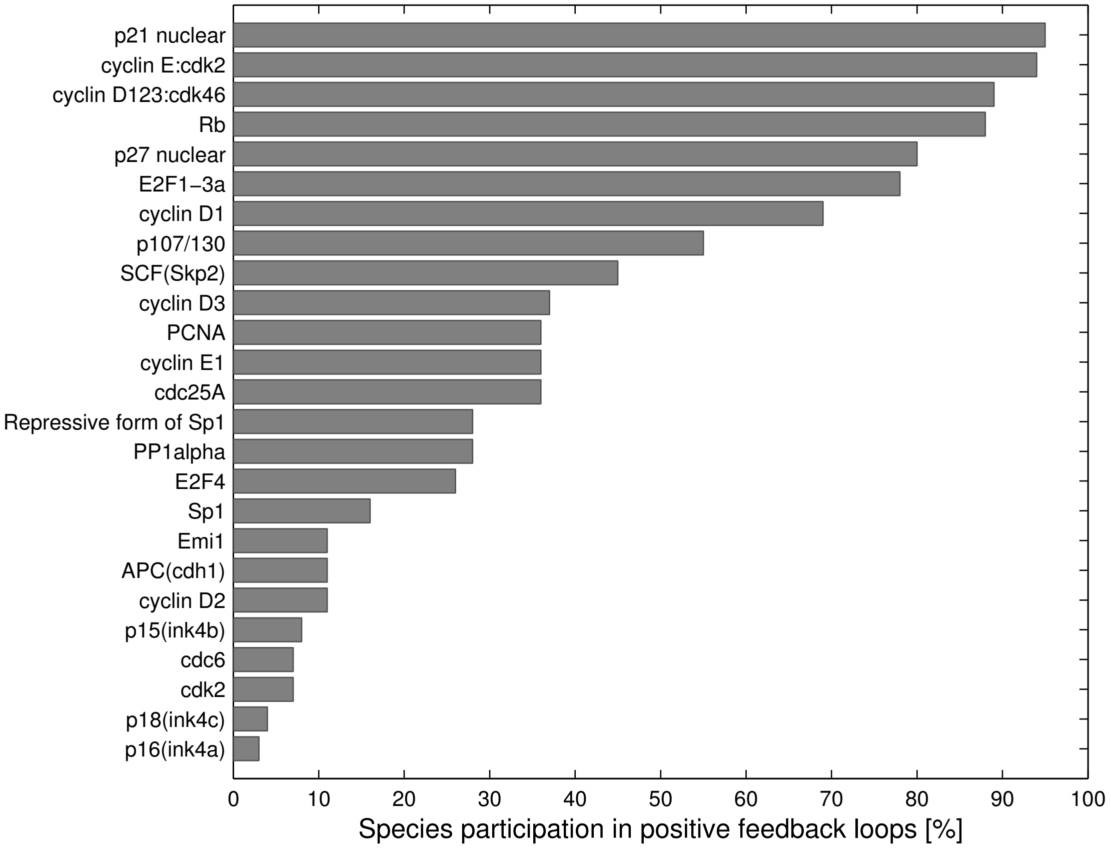

Fig. S5 – Species participation in positive feedback loops of the layer 'Cell Cycle (G1/S)'. The participation of species in positive feedback loops of the layer 'Cell Cycle (G1/S)' (Fig. 2 and Doc. S2) was calculated and is given in % of all positive feedbacks in this section. The nuclear forms of the CDK inhibitors p21 and p27, the activated CDKs 2,4, and 6, Rb as well as E2F1-3a proved to be part of most cycles. Positive feedback loops are essential to convey bistability to a network, e.g. to realize the restriction point at the G1/S transition.

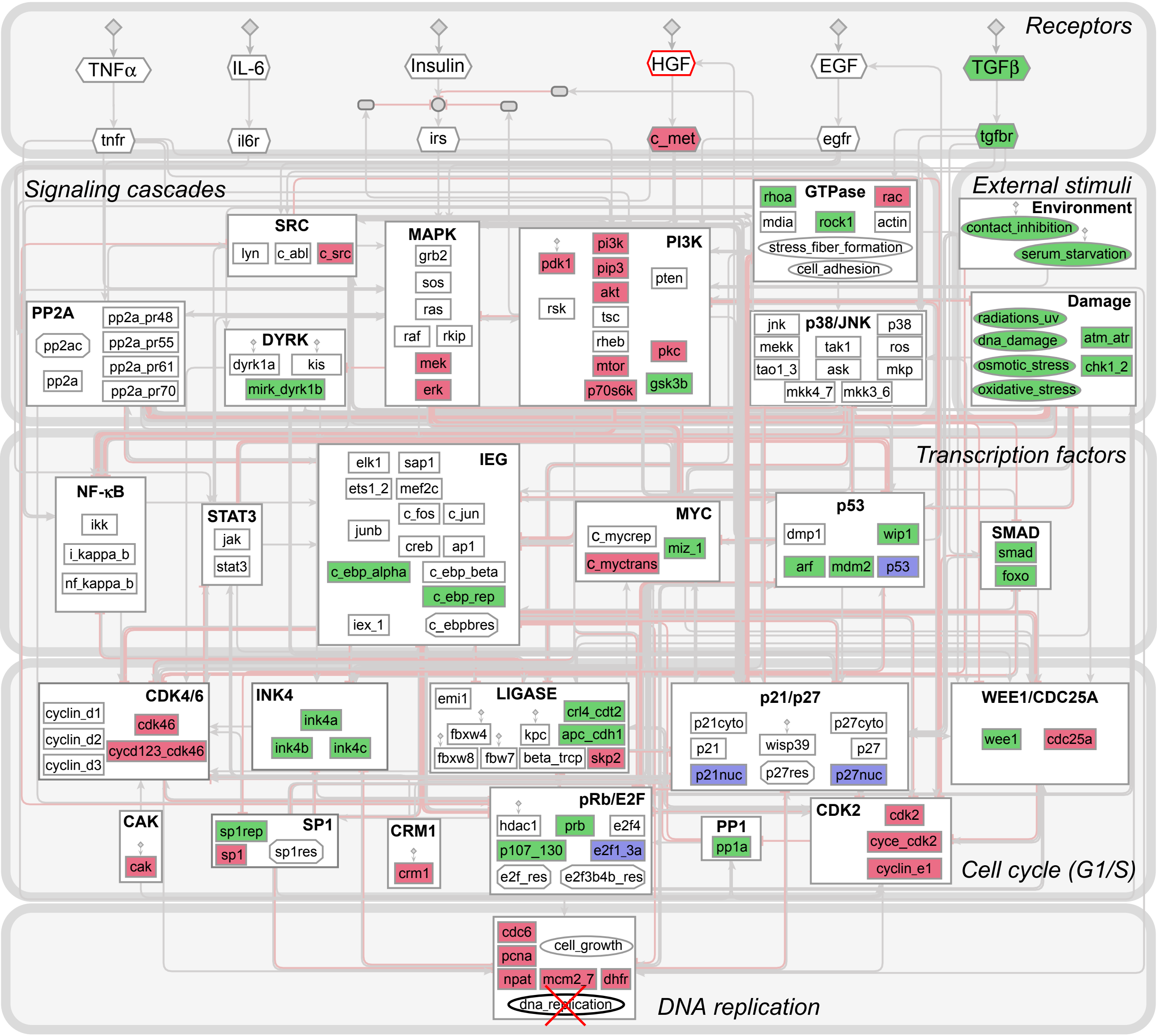

Species has to be:

- fully activated
- inhibited
- either inhibited or fully activated

Fig. S6 – **MIS of size '1' to block HGF-induced DNA synthesis** Minimal intervention sets (MIS) leading to the state *dna_replication* = '0' (red cross) although the input HGF is set to '1' (red hexagon) were computed. Species are color-coded according to their required activation state (lower left corner) and depicted in the schematic model (see also Fig. 2). Four species are colored in blue to indicate their ambiguous role. Depending on their activation level, they can be either activated or inhibited to mediate a block of HGF-stimulated DNA synthesis.

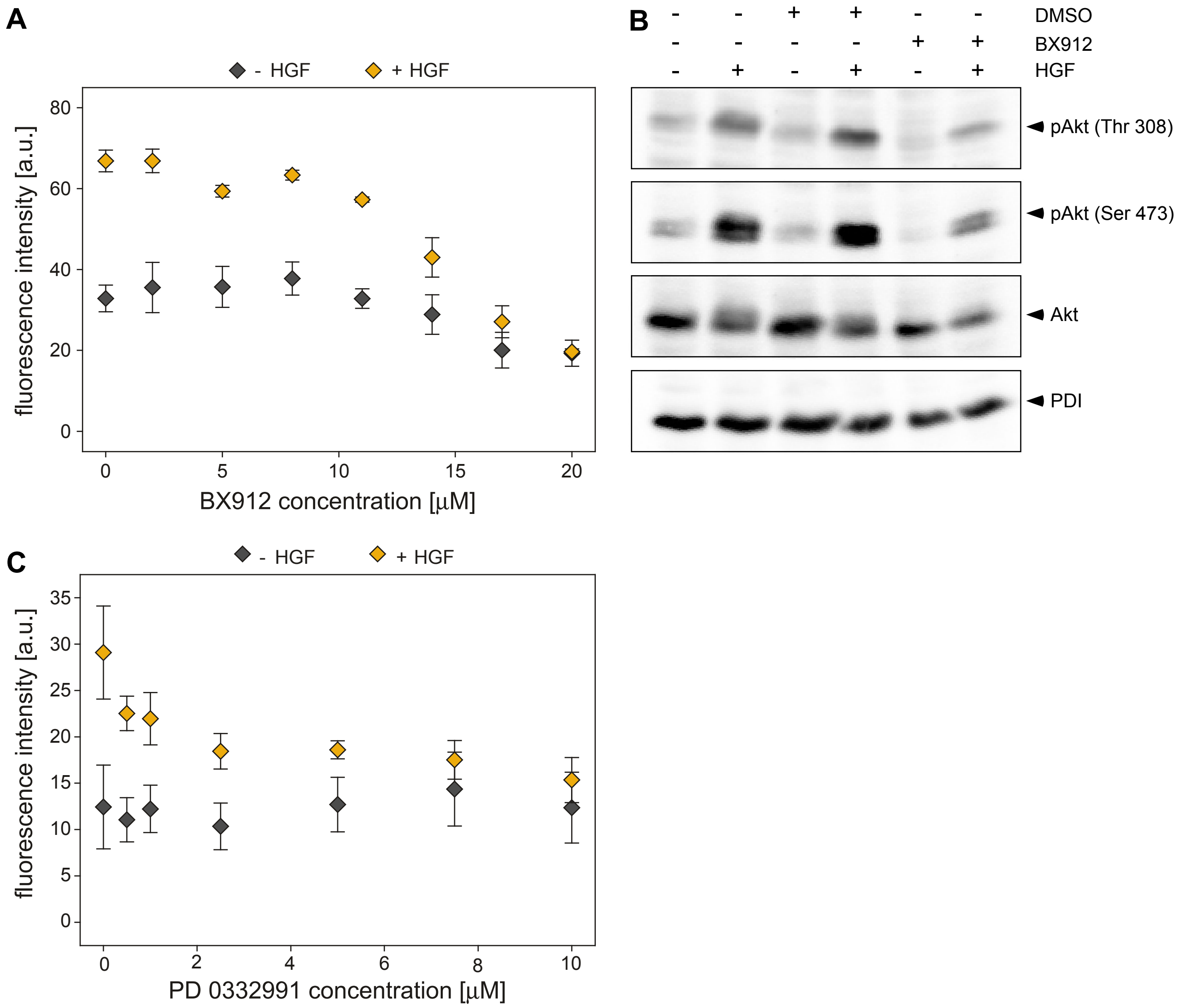

Fig. S7 – **Inhibitor performance. A.** Primary mouse hepatocytes were cultivated in the presence of increasing doses of BX912 or equal volumes of its solvent DMSO. After 30 min of inhibitor pretreatment, hepatocytes were additionally stimulated with 40 ng/ml HGF or left untreated for 48 h. DNA content was assayed using Sybr® Green I. Data represent mean fluorescence intensity of technical triplicate measurements in arbitrary units (a.u.). Error bars indicate standard deviation of triplicates. **B.** Primary mouse hepatocytes were cultivated in the presence of 15 μM BX912, equal volumes of DMSO, or without inhibitor. After 30 min of inhibitor pretreatment, cells were stimulated with 40 ng/ml HGF for 10 min. Cells were lysed and 30 μg of total protein were resolved on a SDS-PAGE and blotted on a PVDF membrane. Western blots for phosphorylated Akt at threonine 308 (pAkt (Thr308)) and serine 473 (pAkt (Ser 473)), as well as total Akt (Akt) and PDI as loading control are shown. For further methodical information refer to Doc. S4. **C.** Primary mouse hepatocytes were cultivated in the presence of increasing doses of PD0332991 or equal volumes of its solvent DMSO. After 30 min of inhibitor pretreatment, hepatocytes were additionally stimulated with 40 ng/ml HGF or left untreated for 48 h. DNA content was assayed using Sybr® Green I. Data represent mean fluorescence intensity of technical triplicate measurements in arbitrary units [a.u.]. Error bars indicate standard deviation of triplicates.

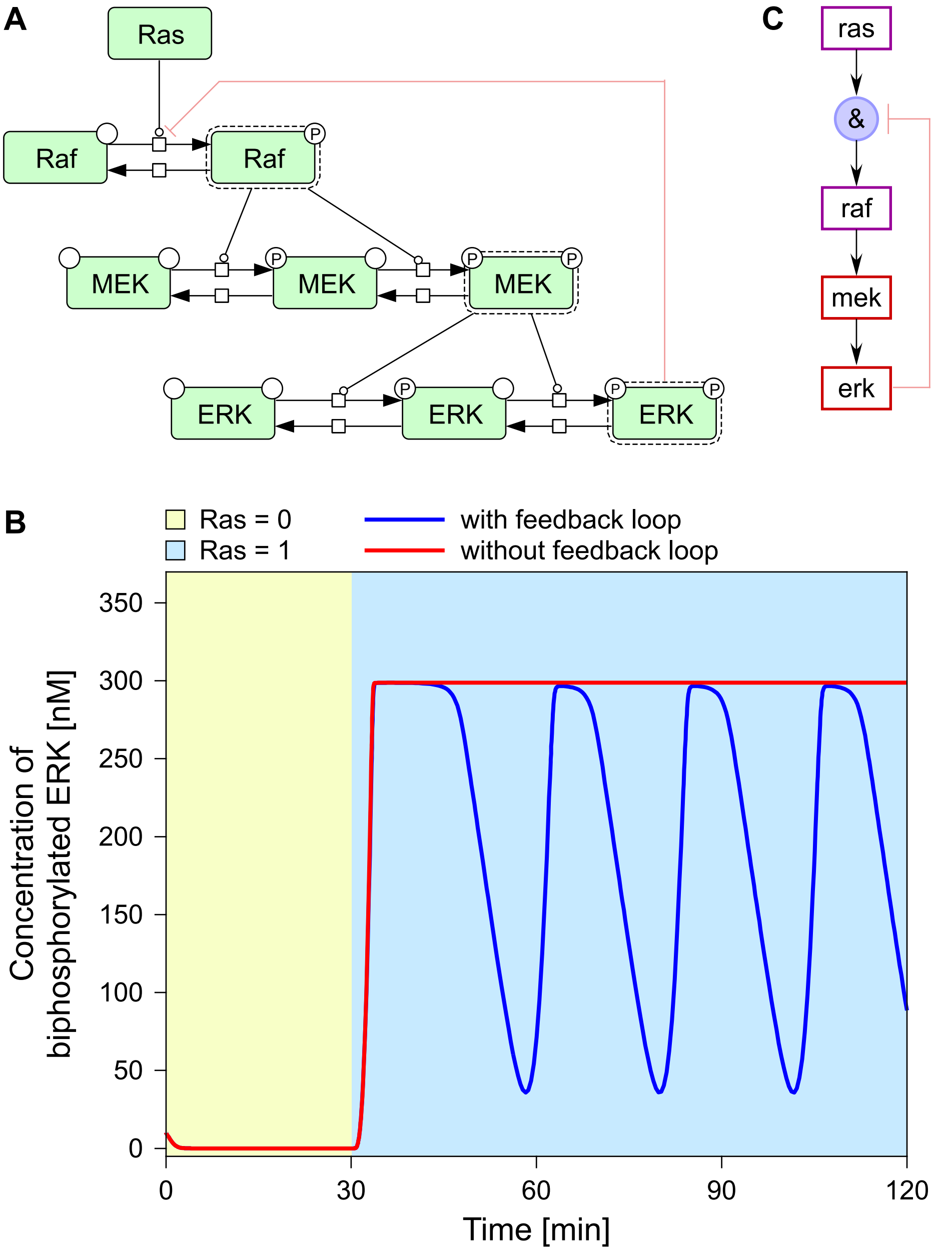

Fig. S8 – Initial response and feedback loops A. Representation of the MAPK cascade as modeled by Kholodenko *et al.* [1]. **B.** Corresponding logical structure. Typically, to allow computation of the initial response the negative feedback loop between ERK and Ras must be opened. **C.** Dynamical course of active ERK concentration using the model of Kholodenko *et al.* ERK steady state in quiescence was obtained by setting Ras to '0' at t=0. At t=30min Ras was set to '1'. The blue curve depicts the dynamics of the original model showing oscillations. The red curve represents the same model with no negative feedback loop between ERK and Ras. Although oscillations arise in the presence of the negative feedback loop, the initial response (phosphorylation of ERK) remains the same.

Doc. S2 Documentation of the original model

2.1 Documentation of the equations contained in the original model

Nº	Reaction	Time scale	References
1	e2f45repress → inh_cdc25a	1	E2F45 has been shown to repress cdc25A in complex with p130 and HDAC1 [2,3].
2	rock1 → inh_cdc25a	1	p160^{ROCK} phosphorylates and inhibits CDC25A after TGF-β signalling, leading to cell cycle arrest (shown in epithelial cells) [4].
3	$2\ \text{e2f1\_3a} \cdot \overline{\text{c\_ebp\_rep}} \rightarrow \text{tf\_cdc25a}$	1	Cdc25A is expressed by E2F1-3 [5].<br>A complex formed by pRb, E2F1-3 and either C/EBP-α or -β represses E2F target genes [6,7].
4	apc_cdh1 → inh_cdc25a	1	[8,9]
5	e2f1_3a · stat3 · free_p21 → inh_cdc25a	1	Repression of cdc25A transcription by binding of p21 to the promoter together with STAT3 and E2F1 [10,11].
6	$\overline{\text{erk}} \cdot \text{ap1} \rightarrow \text{wee1}$	1	Wee1 is expressed by c-Fos/AP-1 (bound to c-Jun, shown in antigen Th1 cells and rheumatoid synovial cells) [12, 13]. It can be phosphorylated (and inhibited) either by Akt [14] (but only during S and G_2 so this interaction is not included) or ERK (shown in vascular smooth muscle) [15]. It is also inhibited by Cdk1 phosphorylation during mitosis, which is not included in our model.
7	beta_trcp · smad → inh_cdc25a	1	[16]
8	$\text{cyce\_cdk2} \cdot \text{tf\_cdc25a} \cdot \overline{\text{inh\_cdc25a}} \rightarrow 2\ \text{cdc25a}$	1	Cdc25A kinase activity is increased by phosphorylation by active cdk2-containing complexes [17].
9	$\text{tf\_cdc25a} \cdot \overline{\text{inh\_cdc25a}} \rightarrow \text{cdc25a}$	1	Cdc25A is regulated at the transcriptional and post-transcriptional levels [4].
10	$\text{c\_myctrans} \cdot \text{stat3} \cdot \overline{\text{c\_ebp\_rep}} \rightarrow \text{tf\_cdc25a}$	1	STAT3 cooperates with Myc to express cdc25a [18]<br>A complex formed by pRb, E2F1-3 and either C/EBP-α or -β represses E2F target genes [6,7].
11	beta_trcp · chk1_2 → inh_cdc25a	1	Actually ubiquitination by SCF$^{\beta-TrCP}$ is triggered by phosphorylation by an unknown kinase but this kinase seems activated after DNA damage like Chk1/2. Furthermore phosphorylation by Chk1/2 seems to be a prior requirement for degradation although it is not directly involved in β-TrCP binding [8].
12	p38 · osmotic_stress → inh_cdc25a	1	p38 phosphorylates cdc25A upon osmotic stress [8,19].
13	2 prb · stat3 → inh_cdc25a	1	STAT3 is recruited to the Cdc25A promoter by unphosphorylated pRb to repress its expression [20].
14	e2f45repress → repress_cyce	1	[21]
15	$\text{sp1} \cdot \overline{\text{c\_ebp\_alpha}} \rightarrow \text{cdk2}$	1	Sp1 induces CDK2 expression [22] but C/EBPα inhibits CDK2 kinase activity by binding [7].
16	c_myctrans → trans_cyce	1	Myc expresses cyclin E [23].
17	e2f1_3a → trans_cyce	1	E2F1-3 expresses cyclin E [24].
18	c_ebp_rep → repress_cyce	1	A complex formed by pRb, E2F1-3 and either C/EBP-α or -β represses E2F target genes [6,7].

Continued on next page

Nº	Reaction	Time scale	References
19	cyce_cdk2 → cyce_cdk2_2	1	Used for model comparison with scenarios. Since `cyce_cdk2` can adopt several values, which cannot be distinguish in the scenarios, this reaction allows `cyce_cdk2_2` to be equal to '1' when cyce_cdk2 > '0'.
20	2 p21nuc → inh_cdk2	1	High concentrations of p21 (e.g. after stress) inhibits CDK2 activity by binding [25,26].
21	sp1rep → repress_cyce	1	A complex formed by pRb, E2F1-3, Sp1 and HDAC has been shown to actively repress cyclin E expression [21].
22	$\overline{\text{wee1}}$ · cdc25a → activ_cdk2	1	cdc25A dephosphorylates CDK2 to increase its kinase activity [27].<br>Wee1 can phosphorylate cyclin E:CDK2 [28].
23	fbw7 · cyce_cdk2 → inh_cdk2	2	Fbw7 ubiquitinates cyclin E after phosphorylation by either GSK3-β alone (dimeric Fbw7) or by GSK3-β and/or active CDK2 (monomeric Fbw7) [29]. Oncogenic Ras also prevents cyclin E turnover by Fbw7 but this interaction is not included in the model since it does not involve wild type Ras [30].
24	skp2 · $\overline{\text{cdk2}}$ → inhib_free_cyce	1	Skp2 ubiquitinates the free, non-phosphorylated form of cyclin E [31].
25	trans_cyce · $\overline{\text{repress\_cyce}}$ · $\overline{\text{inhib\_free\_cyce}}$ → cyclin_e1	1	
26	$\overline{\text{wee1}}$ · 2 cdc25a → 2 activ_cdk2	1	cdc25A dephosphorylates CDK2 to increase its kinase activity [27].<br>The autoamplification loop between cdc25A and CDK2 provides a level '2' to cyclin E:CDK2 when cdc25A is fully activated.<br>Wee1 can phosphorylate cyclin E:CDK2 [28].
27	fbw7 · gsk3b → inh_cdk2	1	Fbw7 ubiquitinates cyclin E after phosphorylation by either GSK3-β alone (dimeric Fbw7) or by GSK3-β and/or active CDK2 (monomeric Fbw7) [29]. Oncogenic Ras also prevents cyclin E turnover by Fbw7 but this interaction is not included in the model since it does not involve wild type Ras [30].
28	$\overline{\text{3 p27nuc}}$ · p21nuc · $\overline{\text{inh\_cdk2}}$ · cyclin_e1 · cdk2 · activ_cdk2 · cak → cyce_cdk2	1	The complex formed by cyclin E and CDK2 proteins must first enter the nucleus to be active. This mechanism is supposed to be dependent on p21 [26,32].<br>p27 inhibits CDK2 activity [33] and its nuclear export allows the activation of `cyce_cdk2` to a level '1'. Furthermore, this activation is allowed by the expression of the cdc25A phosphatase, which removes inhibitory phosphorylations. This mechanism is embedded in `activ_cdk2`. This initiates a positive feedback loop where CDK2 phosphorylates cdc25A to increase its activity [27]. `activ_cdk2` adopts the value '2' and can then increasingly activate `cyce_cdk2` to a level '2'. We suppose that from this activity level, active, free cyclin E:CDK2 complexes are sufficiently numerous to inhibit p27 by phosphorylation on T187 [34]. This reduced level of p27 allows full activation of cyclin E:CDK2, which can now phosphorylate pRb [35].

Continued on next page

Nº	Reaction	Time scale	References
29	$\overline{3\ \text{p27nuc}} \cdot \text{p21nuc} \cdot \overline{\text{inh\_cdk2}} \cdot \text{cyclin\_e1} \cdot \text{cdk2} \cdot 2\ \text{activ\_cdk2} \cdot \text{cak} \rightarrow 2\ \text{cyce\_cdk2}$	1	The complex formed by cyclin E and CDK2 proteins must first enter the nucleus to be active. This mechanism is supposed to be dependent on p21 [26,32].<br>p27 inhibits CDK2 activity [33] and its nuclear export allows the activation of `cyce_cdk2` to a level '1'. Furthermore, this activation is allowed by the expression of the cdc25A phosphatase, which removes inhibitory phosphorylations. This mechanism is embedded in `activ_cdk2`. This innitiates a positive feedback loop where CDK2 phosphorylates cdc25A to increase its activity [27]. `activ_cdk2` adopts the value '2' and can then increasingly activate `cyce_cdk2` to a level '2'. We suppose that from this activity level, active, free cyclin E:CDK2 complexes are sufficiently numerous to inhibit p27 by phosphorylation on T187 [34]. This reduced level of p27 allows full activation of cyclin E:CDK2, which can now phosphorylate pRb [35].
30	$\overline{2\ \text{p27nuc}} \cdot \text{p21nuc} \cdot \overline{\text{inh\_cdk2}} \cdot \text{cyclin\_e1} \cdot \text{cdk2} \cdot 2\ \text{activ\_cdk2} \cdot \text{cak} \rightarrow 3\ \text{cyce\_cdk2}$	1	The complex formed by cyclin E and CDK2 proteins must first enter the nucleus to be active. This mechanism is supposed to be dependent on p21 [26,32].<br>p27 inhibits CDK2 activity [33] and its nuclear export allows the activation of `cyce_cdk2` to a level '1'. Furthermore, this activation is allowed by the expression of the cdc25A phosphatase, which removes inhibitory phosphorylations. This mechanism is embedded in `activ_cdk2`. This initiates a positive feedback loop where CDK2 phosphorylates cdc25A to increase its activity [27]. `activ_cdk2` adopts the value '2' and can increasingly activate `cyce_cdk2` to a level '2'. We assume that from this activity level, active, free cyclin E:CDK2 complexes are sufficiently numerous to inhibit p27 by phosphorylation on T187 [34]. This reduced level of p27 allows full activation of cyclin E:CDK2, which can now phosphorylate pRb [35].

Continued on next page

№	Reaction	Time scale	References
31	deg_cycd123 → deg_cycd3	1	
32	foxo → repress_cyclin_d1	1	cyclin D1 transcription is repressed by FoxO [36,37].
33	junb → repress_cyclin_d1	1	cyclin D1 transcription is repressed by JunB [38].
34	cyclin_d2 → cyclin_d123	1	
35	cyclin_d3 → cyclin_d123	1	
36	ets1_2 → trans_cyclin_d1	1	[39]
37	nf_kappa_b → trans_cyclin_d1	1	[40]
38	creb → trans_cyclin_d1	1	[41]
39	gsk3b → phospho_cycd123	1	Cyclin D1 [42] on T286, cyclin D2 [43] on T280 and cyclin D3 [44,45] on T283 leading to their degradation..
40	ink4 → inh_cdk46	1	[46]
41	e2f4 → trans_cyclin_d1	1	[47]
42	p27nuc → cip_kip	1	
43	2 p21nuc → inh_cdk46	1	Full activation of either p21 or p27 inhibits CDK4/6 kinase activity (probably depending on the number of cip/kip molecules bound to the kinase) [26,48].
44	3 p27nuc → inh_cdk46	1	Full activation of either p21 or p27 inhibits CDK4/6 kinase activity (probably depending on the number of cip/kip molecules bound to the kinase) [26,48].
45	p21nuc → cip_kip	1	
46	c_ebp_alpha → inh_cdk46	1	C/EBPα inhibits CDK4 kinase activity by binding [7].
47	fbx4 → ligase_cycd123	1	[49]
48	fbxw8 → ligase_cycd123	1	[50]
49	c_myctrans → trans_cyclin_d1	1	We chose to model an activatory effect of Myc on cyclin D1 [51,52] although the situation is not clear here. Earlier reports have stated a repressive effect of Myc (independently of Max) on cyclin D1 expression [53].
50	ap1 → trans_cyclin_d1	1	[38]
51	p38 → phospho_cycd123	1	After stress, p38 can phosphorylate cyclin D1 on T286 [42,54], cyclin D2 on T280 [43] and cyclin D3 on T283 leading to their degradation [55].
52	cyclin_d1 → cyclin_d123	1	
53	deg_cycd123 → deg_cycd1	1	
54	c_myctrans → cdk46	1	Myc expresses CDK4 [56].
55	p27nuc · p21nuc → assembly_import_cdk46	1	We suppose here that `p21nuc`=1 and `p27nuc`=1 are necessary for assembly [48,57] and nuclear import of cyclin D:CDK4/6 complexes [32].
56	$\overline{\text{deg\_cycd123}}$ · activ_cycd2 → cyclin_d2	1	
57	$\overline{\text{foxo}}$ · c_myctrans → activ_cycd2	1	c-Myc induces cyclin D2 expression [58]. FoxO can repress cyclin D2 expression [37].
58	2 e2f1_3a · $\overline{\text{c\_ebp\_rep}}$ · $\overline{\text{deg\_cycd3}}$ → cyclin_d3	1	cyclin D3 is expressed by E2F1-3 [59].<br>A complex formed by pRb, E2F1-3 and either C/EBP-α or -β represses E2F target genes [6,7].

Continued on next page

Nº	Reaction	Time scale	References
59	sp1rep → repress_cyclin_d1	1	cyclin D1 transcription is repressed by a complex formed of E2F1 and Sp1 [47].
60	stat3 → trans_cyclin_d1	1	[60]
61	crm1 · phospho_cycd123 · ligase_cycd123 · $\overline{\text{cip\_kip}}$ → deg_cycd123	1	cyclin D is exported from the nucleus after phosphorylation and degraded after ubiquitination [42]. However, binding of p21 or p27 prevents CRM1 binding and thus nuclear export and its subsequent degradation [61]. This is the only considered mechanism of cyclin D:Cdk4/6 complexes activation by CKI.<br>We here assume that this is true for all 3 cyclin D.
62	$\overline{\text{pp1a}}$ · crm1 · ligase_cycd123 · $\overline{\text{cip\_kip}}$ → deg_cycd3	1	cyclin D3 can be degraded by 2 mechanisms: one involves phosphorylation by GSK3-β similar to the mechanism of the other D-type cyclins, while the other GSK3-β is independent and involves a (constitutive?) phosphorylation site dephosphorylated by PP1 [45].<br>However, binding of p21 or p27 prevents CRM1 binding and thus nuclear export and its subsequent degradation [61]. We here assume that this is true for all 3 cyclin D.
63	mirk_dyrk1b · crm1 · ligase_cycd123 · $\overline{\text{cip\_kip}}$ → deg_cycd1	1	Mirk induces cyclin D1 degradation by phosphorylation on T288 [42].<br>cyclin D1 is exported after phosphorylation and degraded after ubiquitination [42]. However,k binding of p21 or p27 prevents CRM1 binding and thus nuclear export and its subsequent degradation [61].<br>Hence, this is the only considered mechanism of activation of cyclin D:Cdk4/6 complexes by CKI.
64	$\overline{\text{contact\_inhibition}}$ · $\overline{\text{inh\_cdk46}}$ · cyclin_d123 · cdk46 · assembly_import_cdk46 · cak → cycd123_cdk46	1	Phosphorylation by CAK is attenuated by contact inhibition [62].<br>p21 seems necessary for CDK4 nuclear import in primary hepatocytes [32].<br>We assume that `p21nuc`=1 and `p27nuc`=1 are necessary for assembly [48,57] and nuclear import of cyclin D:CDK4/6 complexes [32].
65	trans_cyclin_d1 · $\overline{\text{repress\_cyclin\_d1}}$ · $\overline{\text{deg\_cycd1}}$ → cyclin_d1	1	cyclin D1 transcription is repressed by JunB [38], E2F1 [47] or FoxO [36,37].
66	p21cyto → p21	1	
67	p21nuc → p21	1	
68	pkc → p21s153	1	[63]
69	akt → p21t145	1	[63,64]
70	p38 → p21t57stabil	1	p21 is phosphorylated and stabilized by p38α and JNK1 [65]. In addition, p38 positively affects p21 via HubR. p38 MAPK phosphorylates the mRNA binding protein of HuR on T118, which results in cytoplasmic accumulation of HuR and its enhanced binding to the p21^{Cip1} mRNA resulting in its stabilition [66].<br>A positive interaction between p38 and p21 was also confirmed by [67].

Continued on next page

Nº	Reaction	Time scale	References
71	$\overline{\text{sp1rep}} \cdot \text{sp1} \cdot 2\ \text{e2f1\_3a} \cdot \overline{\text{c\_ebp\_rep}} \cdot \overline{\text{rhoa}} \rightarrow \text{tf\_p21}$	1	Gartel *et al.* showed that E2F1 DNA and Sp1 binding domains are both required for high level induction of p21 expression [68].<br>RhoA represses p21 expression [69–71].<br>A complex (`sp1rep`) containing Sp1 and HDAC can bind promoters of Sp1 target genes and repress their expression [72].<br>A complex (`c_ebp_rep`) formed by pRb, E2F1-3 and either C/EBP-α or -β represses E2F target genes [6,7].
72	jnk → p21t57stabil	1	p21 is phosphorylated and stabilized by p38α and JNK1 [65].
73	erk → p21s130degcyto	1	[73]
74	crm1 · p21s153 → transloc_p21	1	[63]
75	crm1 · p21t145 → transloc_p21	1	[63]
76	wisp39 · p21s146 → stabil_p21	1	Phosphorylation on S146 seems to stabilize p21 [64].<br>Binding of Wisp39 has been shown to stabilize p21 and even is required for stabilizing p21 after IR radiations [74].<br>However, it is not clear if p21 has to be phosphorylated to allow Wisp39 to bind or not. Hence, we use here an Incomplete Truth Table gate.
77	wisp39 · p21t57stabil → stabil_p21	1	Phosphorylation on T57 seems to stabilize p21 [64].<br>Binding of Wisp39 has been shown to stabilize p21 and even is required for stabilizing p21 after IR radiations [74].<br>However, it is not clear if p21 has to be phosphorylated to allow Wisp39 to bind or not. Hence, we use here an Incomplete Truth Table gate.
78	p21s130degcyto → deg_p21_cyto	1	[73]
79	$\text{skp2} \cdot \overline{\text{pcna}} \cdot \text{p21s130degnuc} \rightarrow \text{deg\_p21\_nuc}$	1	Skp2 ligase induces p21 degradation at the G1/S transition in unperturbed cells [75] after phosphorylation on S130 by the cyclin E:Cdk2 complex [76]. Ubiquitination by Skp2 can only affect nuclear p21 since Skp2 is exclusively nuclear [77].<br>In contrast to the case of p27, this process is not necessary for downregulation of p21 at the G_1/S transition [76].<br>Skp2 was also shown to ubiquinate p21 but only upon low doses of UV irradiations [8].<br>Binding to PCNA abolishes p21 contact with the proteasome [63]. This affects only the degradation of nuclear p21 since PCNA is nuclear.

Continued on next page

Nº	Reaction	Time scale	References
80	p21s114 → deg_p21_cyto	1	Phosphorylation on S114 targets p21 for degradation by the proteasome [78].
81	p21t57deg → deg_p21_cyto	1	Phosphorylation on T57 makes p21 unstable [63,73].
82	c_ebp_alpha → stabil_p21	1	p21 is stabilized by binding of C/EBPα [79].
83	erk → p21t57deg	1	ERK2 phosphorylates p21 on T57 [73].
84	jnk → p21s130stabil	1	Phosphorylation on S130 by JNK seems to stabilize p21 [63].
85	wisp39 · p21s130stabil → stabil_p21	1	Phosphorylation on S130 seems to stabilize p21 [64].<br>Binding of Wisp39 has been shown to stabilize p21 and even is required for stabilizing p21 after IR radiations [74].<br>However, it is not clear if p21 has to be phosphorylated to allow Wisp39 binding or not. Hence, we use here an Incomplete Truth Table gate.
86	p38 → p21s130stabil	1	Phosphorylation on S130 by p38α seems to stabilize p21 [63].
87	akt → p21s146	1	[64]
88	erk → p21s130degnuc	1	ERK2 phosphorylates p21 on S130 [73].
89	2 tf_p21 → 2 p21nuc	1	Nuclear p21^{Cip} is fully active (or simply abundant) (equivalent to a level '2') in case of stress (relayed by p53) [26].
90	$\overline{\text{pcna}}$ · p21s114 → deg_p21_nuc	1	Phosphorylation on S114 leads to degradation by the proteasome [78].<br>Binding to PCNA abolishes p21 contact with the proteasome [63]. This affects only the degradation of nuclear p21 since PCNA is nuclear.
91	$\overline{\text{pcna}}$ · p21t57deg → deg_p21_nuc	1	Phosphorylation on T57 makes p21 unstable [63,73].<br>Binding to PCNA abolishes p21 contact with the proteasome [63]. This affects only the degradation of nuclear p21 since PCNA is nuclear.
92	$\overline{\text{sp1rep}}$ · $\overline{\text{c\_ebp\_rep}}$ · c_ebp_alpha → tf_p21	1	C/EBPα induces p21 transcription [80].<br>A complex (`c_ebp_rep`) containing either Sp1 or C/EBPα or β can repress p21 transcription [7,72].<br>A complex (`sp1rep`) containing Sp1 and HDAC can bind promoters of Sp1 target genes and repress their expression [72].
93	crl4_cdt2 · pcna → deg_p21_nuc	1	CRL4^{Cdt2} ligase induces p21 degradation after UV irradiations. This process seems to require prior PCNA binding [64].
94	gsk3b → p21s114	1	p21 can be phosphorylated on S114 by GSK3β after UV irradiation, which also targets it for degradation by the proteasome [78].
95	mirk_dyrk1b → p21s153	1	Phosphorylation on S153 by PKC or Dyrk1B induces p21 cytoplasmic accumulation.<br>Under normal conditions, Dyrk1B is a nuclear kinase, so it might not play a key role in phosphorylating p21 in the cytoplasm [81].<br>The phosphorylation by PKC is prevented by binding of Ca2+/CaM to the p21 NLS. However, S153-phosphorylated p21 can not bind CaM [63].

Continued on next page

Nº	Reaction	Time scale	References
96	2 p21nuc → free_p21	1	We assume that p21 can fulfill its inhibitory functions only when released from cyclin D:Cdk4/6 complexes. Activation of the p53 pathway is also supposed to activate p21nuc with a level of '2' and this leads to higher concentrations of p21 compared to cyclin D:CDK complexes.
97	$\overline{\text{sp1rep}} \cdot \text{smad} \cdot \text{miz\_1} \cdot \overline{\text{c\_ebp\_rep}} \cdot \overline{\text{rhoa}} \cdot \text{foxo\_sp1} \rightarrow \text{tf\_p21}$	1	Smad-FoxO activates p21 expression [82] . Smad-Sp1 has been shown to have a similar effect, while requiring presence of Miz1 on the core promoter [83,84].<br>RhoA represses p21 expression [69,70].<br>A complex (`c_ebp_rep`) formed by pRb, E2F1-3 and either C/EBP-α or -β represses E2F target genes [6,7].<br>A complex (`sp1rep`) containing Sp1 and HDAC can bind promoters of Sp1 target genes and repress their expression [72].
98	gsk3b → p21t57deg	1	GSK3-β phosphorylation on T57 reduces p21 stability. This process can be prevented by binding to PCNA which abolishes p21 contact with the proteasome [63].
99	$\overline{\text{sp1rep}} \cdot \text{sp1} \cdot \text{p53} \cdot \text{miz\_1} \cdot \overline{\text{c\_ebp\_rep}} \cdot \overline{\text{rhoa}} \rightarrow \text{tf\_p21}$	1	p53 requires both Sp1 [85] and Miz1 [84] to activate the p21 promoter.<br>RhoA represses p21 expression [69,70].<br>A complex (`sp1rep`) containing Sp1 and HDAC can bind promoters of Sp1 target genes and repress their expression [72].<br>A complex (`c_ebp_rep`) formed by pRb, E2F1-3 and either C/EBP-α or -β represses E2F target genes [6,7].
100	cyce_cdk2 → p21s130degnuc	1	Skp2 ligase induces p21 degradation at the G1/S transition in unperturbed cells [75] after phosphorylation on S130 by the cyclin E:Cdk2 complex [76]. Ubiquitination by Skp2 can only affect nuclear p21 since Skp2 is exclusively nuclear [77].<br>In contrast to the case of p27, this process is not necessary for downregulation of p21 at the G_1/S transition [76].<br>Skp2 was also shown to ubiquinate p21 but only upon low doses of UV irradiations [8].
101	$\text{tf\_p21} \cdot \text{stabil\_p21} \cdot \overline{\text{deg\_p21\_nuc}} \rightarrow \text{p21nuc}$	1	p21 is primarily absent in quiescent cells and becomes expressed after mitogenic stimulation [32]. Since p21 is quite unstable [64], we assume it requires stabilization to be active.
102	$\overline{\text{sp1rep}} \cdot \text{stat3} \cdot \overline{\text{c\_ebp\_rep}} \cdot \overline{\text{rhoa}} \cdot 2\ \overline{\text{cyclin\_d1}} \rightarrow \text{tf\_p21}$	1	Only overexpressed cyclin D1 seems to repress p21 expression [86–88] , and this effect is mediated via recruitment of cyclin D1 to the p21 promoter by STAT3 [88].<br>RhoA represses p21 expression [69,70].<br>A complex (`c_ebp_rep`) formed by pRb, E2F1-3 and either C/EBP-α or -β represses E2F target genes [6,7].<br>A complex (`sp1rep`) containing Sp1 and HDAC can bind promoters of Sp1 target genes and represses their expression [72].

Continued on next page

Nº	Reaction	Time scale	References
103	$\text{transloc\_p21} \cdot \text{tf\_p21} \cdot \text{stabil\_p21} \cdot \overline{\text{deg\_p21\_cyto}} \rightarrow \text{p21cyto}$	1	p21 is primarily absent in quiescent cells and becomes expressed after mitogenic stimulation [32]. Since p21 is quite unstable, we assume it also requires stabilization to be active.<br>p21 can be present in both the cytoplasm and the nucleus where it has different functions [63,89,90].<br>It is supposed to be present in the cytoplasm only if translocated to the cytoplasm and not degraded.
104	$\overline{\text{sequester\_cki}} \cdot \text{tf\_p21} \cdot \text{stabil\_p21} \cdot \overline{\text{deg\_p21\_nuc}} \rightarrow 2\ \text{p21nuc}$	1	p21 is primarily absent in quiescent cells and becomes expressed after mitogenic stimulation [32]. Since p21 is quite unstable [64], we assume it requires stabilization to be active.<br>p21 can be present in both the cytoplasm and the nucleus where it has different functions [63,89,90].<br>Nuclear p21 is fully active (or simply abundant) (equivalent to a level '2') if not bound by cyclin D:CDK4/6 complexes [61].
105	$\overline{\text{transloc\_p21}} \cdot \text{tf\_p21} \cdot \text{stabil\_p21} \cdot \overline{\text{deg\_p21\_nuc}} \rightarrow 2\ \text{p21nuc}$	1	p21 is primarily absent in quiescent cells and becomes expressed after mitogenic stimulation [32]. Since p21 is quite unstable [64], we assume it requires stabilization to be active.<br>p21 can be present in both the cytoplasm and the nucleus where it has different functions [63,89,90].<br>Nuclear p21 is fully active (or simply abundant) (equivalent to a level '2') if not exported to the cytoplasm [63].
106	$\overline{\text{sp1rep}} \cdot \text{sp1} \cdot \text{p53} \cdot 2\ \text{miz\_1} \cdot \overline{\text{c\_ebp\_rep}} \cdot \overline{\text{rhoa}} \rightarrow 2\ \text{tf\_p21}$	1	In case of activation of p53 by stress (level '2'), p21 is expressed to stop the cell cycle [91]. To distinguish this inhibitory from its activatory function, we confer p21 here a level '2'.
107	$\text{sp1} \cdot \text{foxo} \rightarrow \text{foxo\_sp1}$	1	Smad-FoxO activates p21 expression [82], also a complex Smad-Sp1 has been shown to do so as well [83].<br>We don't know if both, FoxO and Sp1 are required or if one is sufficient, that's why we use an Incomplete Truth Table (ITT) here.
108	$\overline{\text{p27t187}} \cdot \text{p27res} \rightarrow 2\ \text{p27nuc}$	1	Sequestration by cyclin D:CDK4/6 complexes is thought to help decrease p27 concentration in the nucleus below a given threshold to allow further inhibition by the cyclin E:CDK2 complex by phosphorylation on T187 and subsequent degradation [34,92]. When this site is not phosphorylated, we hence confer nuclear p27 a level '2'.
109	$\text{akt} \rightarrow \text{p27s10g1}$	1	[93]
110	$\text{erk} \rightarrow \text{p27s10g1}$	1	[92]
111	$\text{c\_abl} \rightarrow \text{inhib\_p27}$	1	Phosphorylation by c-Src or c-Abl changes p27 conformation and attenuates its cdk inhibitory activity [34,94].
112	$\text{serum\_starvation} \rightarrow 3\ \text{p27nuc}$	1	Serum starvation leads to full induction of p27^{Kip} [95].
113	$\overline{\text{transloc\_p27}} \cdot \text{p27res} \rightarrow 3\ \text{p27nuc}$	1	In this network, translocation is supposed to be a prerequisite for p27 nuclear inhibition. So failing to translocate enables full activation of p27 in the nucleus (level '2') [96,97].

Continued on next page

Nº	Reaction	Time scale	References
114	lyn → inhib_p27	1	Phosphorylation by Src, Lyn or Abl change p27 conformation and inhibits its cdk inhibitory activity [34].
115	c_myctrans → repress_p27	1	c-Myc represses p27 expression by binding directly to its promoter (Inr-dependent mechanism) [71].<br>For this repression Myc must be bound to Max, but Max is not included in this model.<br>Also, Myc has been found to bind FoxO3 directly on the p27 promoter to prevent FoxO3-induced expression of p27 [98].
116	mirk_dyrk1b → p27s10g0	1	Mirk phosphorylates S10 of p27 during G_0, which stabilizes p27 in the nucleus [99,100].
117	p27res → p27nuc	1	We suppose there is a pool of p27 that remains bound to cyclin D:CDK4/6 complexes (without inhibiting them) [100]. This is represented by the the level '1'. This level is necessary since p27 binding prevents cyclin D nuclear export [61].
118	p27cyto → p27	1	Used for comparison with scenarios.
119	p27nuc → p27	1	Used for comparison with scenarios.
120	p27res · $\overline{\text{inhib\_p27}}$ → 3 p27nuc	1	Phosphorylation by Src, Lyn or Abl (contained in `inhib_p27`) changes p27 conformation and attenuates its cdk inhibitory activity [34].<br>Hence lack of this phosphorylation imparts p27 a level '2' since this is the minimal level required to inhibit CDK activity in our model.
121	c_ebp_rep → repress_p27	1	A complex formed by pRb, E2F1-3 and either C/EBP-α or -β represses E2F target genes [6,7].
122	contact_inhibition → 3 p27nuc	1	Contact inhibition leads to full induction of p27 [62].
123	2 e2f1_3a → tf_p27	1	[101]
124	c_src → inhib_p27	1	Phosphorylation by Src, Lyn or Abl change p27 conformation and inhibits its cdk inhibitory activity [34].
125	rsk → p27t198	1	Phosphorylation of p27 at T198 by Akt or RSK prevents nuclear import [100,102].
126	crm1 · p27s10g1 → transloc_p27	1	Phosphorylation on S10 regulates p27 cytoplasmic translocalization [92].
127	crm1 · p27t198 → transloc_p27	1	Phosphorylation on T198 promotes p27 cytoplasmic localization [102].
128	foxo → tf_p27	1	FoxO induces p27 expression [103] but myc can repress p27 transcription [98].
129	kis → p27s10g1	1	[34]
130	akt → p27t198	1	[100]
131	p27s10g0 · p27res → 3 p27nuc	1	Mirk phosphorylates S10 during G_0, which stabilizes p27 in the nucleus [99,100].
132	$\overline{\text{kpc}}$ · transloc_p27 · p27res → p27cyto	1	Cytoplasmic p27 is active only after prior nuclear export and subsequent degradation after ubiquitination by KPC [102]. p27 contains a NLS (nuclear localization sequence) and enters the nucleus if not prevented to do so [102].

Continued on next page

Nº	Reaction	Time scale	References
133	tf_p27 · $\overline{\text{repress\_p27}}$ → 3 p27nuc	1	Transcription (for instance by E2F1 transcription factors [101]) is supposed to activate p27 fully.
134	$\overline{\text{sequester\_cki}}$ · p27res → 3 p27nuc	1	We assume that sequestration by cyclin D:CDK4/6 complexes [102] is also required for p27 nuclear inhibition. This might help decreasing p27 concentration in the nucleus below a given threshold (here level '1') to allow further inhibition by the cyclin E:CDK2 complex.
135	crm1 · phospho_cycd123 · cdk46 · activ_cycd2 → transloc_p27	1	cyclin D2:cdk4/6 complexes contribute to nuclear export of p27. Indeed cyclin D2 is phosphorylated and exported to the cytoplasm and carries bound Cdk4/6 and p27 with it [96,97].
136	skp2 · 2 cyce_cdk2 → p27t187	1	Phosphorylation on T187 by cyclin E:CDK2 complexes leads to ubiquitination by Skp2 and degradation [34].
137	$\overline{\text{kpc}}$ · transloc_p27 · tf_p27 · $\overline{\text{repress\_p27}}$ → p27cyto	1	Cytoplasmic p27 is active only after prior nuclear export and subsequent degradation after ubiquitination by KPC [102]. p27 contains a NLS (nuclear localization sequence) and enters the nucleus if not prevented to do so [102].
138	cyclin_d123 · cdk46 → sequester_cki	1	Binding of the p21 and p27 inhibitors to cyclin D:CDK4/6 complexes prevents them from inhibiting cyclin E:CDK2 complexes [46].
139	radiations_uv → dna_damage	1	[104]
140	pp2a → pp2a_t2_c	2	
141	dna_damage → atm_atr	1	[105]
142	3 e2f1_3a → atm_atr	1	Induction of expression of ATM/ATR was shown for a deregulated E2F1-3, hence the level '3' [106].
143	$\overline{\text{akt}}$ · $\overline{\text{wip1}}$ · $\overline{\text{pp2a\_t2\_c}}$ · atm_atr → chk1_2	1	Chk1 can be phosphorylated on Ser280 by active Akt, which prevents its translocation into the nucleus [107, 108].<br>Furthermore, phosphorylation of Chk1/2 by ATM/ATR is antagonized by the PP2A phosphatase [109] and Wip1 [110].
144	p70s6k → cell_growth	1	[111,112]
145	cyclin_d1 → cycd1_t2	2	
146	2 e2f1_3a · $\overline{\text{c\_ebp\_rep}}$ → cdc6	1	E2F1-3 activates cdc6 expression [5,113].<br>The role of cdc6 is here simplified. Actually cdc6 is required for firing of the replication but during this, it is phophorylated by cyclin A/Cdk2 complexes to avoid rereplication. This phosphorylation is reversed by PP2A:PR48 provoking a cell cycle arrest in case of constitutive activity [114].<br>A complex (`c_ebp_rep`) formed by pRb, E2F1-3 and either C/EBP-α or -β represses E2F target genes [6,7].

Continued on next page

Nº	Reaction	Time scale	References
147	$p53 \cdot \overline{cycd1\_t2} \rightarrow pcna$	1	p53 activates PCNA by binding its promoter [115].
148	$2\ e2f1\_3a \cdot \overline{cycd1\_t2} \rightarrow pcna$	1	E2F activates PCNA expression [116]. cyclin D1 in excess binds PCNA and inhibits its activity [117].<br>p21 (unphosphorylated on T145) associates also with PCNA but it does not seem to have any impact on its activity on DNA replication during cell cycle progression due to an insufficient p21 to PCNA ratio. However, it does after UV radiations [118].
149	$2\ e2f1\_3a \cdot 3\ cyce\_cdk2 \rightarrow mcm2\_7$	1	MCM2-7 needs both, transcription by E2F1-3 transcription factors, and subsequent phosphorylation by cyclin E:CDK2 complexes for activation [119].
150	$2\ e2f1\_3a \cdot 3\ cyce\_cdk2 \rightarrow npat$	1	NPAT levels are regulated by E2F and peaks at G_1/S. Furthermore NPAT needs phosphorylation by cyclin E:CDK2 complexes to activate histone gene transcription [120].
151	$\overline{sp1rep} \cdot sp1 \cdot 2\ e2f1\_3a \cdot \overline{c\_ebp\_rep} \rightarrow dhfr$	1	DHFR is expressed by E2F1-3 in synergy with Sp1 [121,122].<br>A complex (`sp1rep`) containing Sp1 and HDAC can bind promoters of Sp1 target genes and repress their expression [72].<br>A complex (`c_ebp_rep`) formed by pRb, E2F1-3 and either C/EBP-α or -β represses E2F target genes [6,7].
152	$pcna \cdot npat \cdot mcm2\_7 \cdot dhfr \cdot cell\_growth \cdot cdc6 \rightarrow dna\_replication$	1	Cdc6 and Mcm2-7 are required for firing of replication [113,119].<br>NPAT is also essential for DNA replication [120], PCNA and dhfr as well [118,122].
153	$actin \rightarrow stress\_fiber\_formation$	1	[70]
154	$stress\_fiber\_formation \rightarrow cell\_adhesion$	1	[102]
155	$rhoa \rightarrow mdia$	1	[123]
156	$c\_abl \rightarrow rac$	1	[124]
157	$p21cyto \rightarrow p21cyto\_t2\_2$	2	
158	$ras \rightarrow rac$	1	[125]
159	$pip3 \rightarrow rac$	1	PIP3 activates Rac via activation of guanine-nucleotide exchange factors (GEFs) [126].
160	$rhoa \cdot \overline{p21cyto\_t2\_2} \rightarrow rock1$	1	Cytoplasmic p21 can bind to ROCK, inhibiting its kinase activity [90].<br>We use a time scale '2' for the inhibition by p21 to take the gene regulation involved in p21 regulation into account.
161	$rock1 \cdot rac \cdot mdia \rightarrow actin$	1	Rock activates actin stress fiber formation and mDia and Rac1 induces their polymerization and protrusion [70].
162	$tgfbr \cdot \overline{p27t198} \rightarrow rhoa$	1	Phosphorylation of p27 on T198 is thought to facilitate binding to RhoA. Otherwise p27 binds poorly to RhoA [102].<br>This inhibition seems to be cell type-specific, but it was shown in hepatocellular carcinoma cells [127].

Continued on next page

Nº	Reaction	Time scale	References
163	tgfbr · $\overline{\text{2 p27cyto}}$ → rhoa	1	Cytoplasmic p27 [90,123,128] inhibits Rho. This effect seems to be cell type-specific but it was shown to be inhibitory in hepatocellular carcinoma cells [127]. RhoA is activated by TGF-β [129].
164	mef2c → trans_jun	1	p38 activates MEF2c by phosphorylation, which leads to c-jun gene induction [130].
165	p38 → mef2c	1	[130]
166	elk1 → trans_fos	1	[131,132]
167	jnk → elk1	1	[133]
168	rsk → phos_fos	1	RSK has been shown to phosphorylate c-Fos [134].
169	pp2a → pp2a_t2_b	2	
170	$\overline{\text{pp2a}}$ · $\overline{\text{pkc}}$ → c_ebp_alpha	1	PP2a inhibits C/EBPα [135] as well as PKC [136].
171	rsk · $\overline{\text{akt}}$ · c_ebpbres · creb → c_ebp_beta	1	RSK activates C/EBPβ by phosphorylation [135]. CREB induces its expression [136]. Akt seems to inactivate C/EBPβ after Insulin stimulation [137].
172	c_ebp_beta → trans_fos	1	[136]
173	sp1 → trans_jun	1	[138]
174	creb → trans_jun	1	[132]
175	ap1 → trans_jun	1	[132]
176	ap1 → trans_fos	1	[132]
177	creb → trans_fos	1	[132]
178	$\overline{\text{pp1a}}$ · akt → creb	1	CREB is activated through phosphorylation by either Akt or RSK [134,139]. PP1 dephosphorylates CREB [140].
179	erk → elk1	1	[133,141]
180	$\overline{\text{pp1a}}$ · rsk → creb	1	CREB is activated through phosphorylation by either Akt or RSK [134,139]. PP1 dephosphorylates CREB [140].
181	rsk → trans_fos	1	RSK phosphorylates the Serum Response Factor which induces Fos expression [142].
182	sap1 → trans_fos	1	Sap-1 activation (a member of the Ternary Complex Factor family) leads to c-fos gene induction [143].
183	jnk → phos_fos	1	[132,144]
184	erk → phos_fos	1	[132]
185	p38 → sap1	1	p38 activates Sap-1 (a member of the Ternary Complex Factor family) by phosphorylation, which leads to c-fos gene induction [133,143].
186	erk → sap1	1	ERK activates Sap-1 (a member of the Ternary Complex Factor family) by phosphorylation, which leads to c-fos gene induction [133,143].
187	$\overline{\text{sp1rep}}$ · sp1 · $\overline{\text{akt}}$ · $\overline{\text{p53}}$ → iex_1	1	Inhibition of Sp1 activty on the IEX-1 promoter by p53 does not occur through direct interaction but rather via a cofactor binding both p53 and Sp1 [145]. Akt inhibits IEX-1 [146]. A complex containing Sp1 and HDAC can bind promoters of Sp1 target genes and repress their expression [72].

Continued on next page

№	Reaction	Time scale	References
188	smad · nfkbres → junb	1	Smad cooperates with NF-κB to induce JunB expression after TGF-β stimulation [147].<br>However even if NF-κB activity is required, NF-κB activation seems to be mediated differently from the standard IκB pathway. So to account for this effect, we introduced a reservoir for NF-κB, whose presence is required for JunB expression.<br>JunB is also activated by JNK in T cell differentiation [144] but it was not shown to occur during normal cell cycle progression.
189	$\overline{\text{pp1a}}$ · dyrk1a → creb	1	Dyrk1a activates CREB by phosphorylation [148]. Since PP1 dephosphorylates CREB [140], we assume that this mechanism is reversed by PP1.
190	trans_jun · $\overline{\text{deg\_c\_jun}}$ → c_jun_exp	1	
191	c_jun · c_fos → ap1	1	Dimerization of c-Jun with c-Fos increases c-Jun transcriptional activity [130].
192	jnk · $\overline{\text{junb}}$ · c_jun_exp → c_jun	1	After expression, c-jun requires phosphorylation by JNK to become active [132].<br>Dimerization with JunB decreases c-jun transcriptional activity [130].
193	trans_fos · $\overline{\text{pp2a\_t2\_b}}$ · phos_fos → c_fos	1	[132]
194	p38 · erk → elk1	1	ERK and p38 cooperate to activate Elk-1 after UV irradiation [133].
195	erk · ap1 → ets1_2	1	[139]
196	fbw7 · gsk3b → deg_c_jun	1	Fbw7 ubiquitinates c-jun after phosphorylation by GSK3β [30].
197	$\overline{\text{sp1rep}}$ · $\overline{\text{akt}}$ · p53 · nfkbres · $\overline{\text{c\_mycrep}}$ → iex_1	1	p53 alone induces a 26-fold change in IEX-1 expression and NF-κB alone a 70-fold change while both together function synergistically and induce a 148-fold change. So we decide to model this synergy in an AND gate although an OR gate could be correct as well [145].<br>Akt inhibits IEX-1 [146].<br>A complex containing Sp1 and HDAC can bind promoters of Sp1 target genes and repress their expression [72].
198	prb · e2f_res · c_ebp_alpha → c_ebp_rep	1	A complex formed by pRb, E2F1-3 and either C/EBP-α or -β represses E2F target genes [6,7].
199	rsk · $\overline{\text{akt}}$ · stat3 · c_ebpbres → c_ebp_beta	1	RSK activates C/EBPβ by phosphorylation [135].<br>STAT3 induces its expression [136].<br>Akt seems to inactivate C/EBPβ after Insulin stimulation [137].
200	prb · e2f_res · $\overline{\text{rsk}}$ · c_ebpbres · $\overline{\text{cyclin\_d1}}$ → c_ebp_rep	1	A complex formed by pRb, E2F1-3 and either C/EBP-α or -β represses E2F target genes [6,7].<br>However, only its unphosphorylated form (phosphorylation by RSK) or unbound to cyclin D1 can be a repressor [7].

Continued on next page

Nº	Reaction	Time scale	References
201	ink4a → ink4	1	
202	ink4b → ink4	1	
203	ink4c → ink4	1	
204	2 e2f1_3a · $\overline{\text{c\_ebp\_rep}}$ → ink4c	2	E2F1-3a induces Ink4c expression [149].<br>A complex formed by pRb, E2F1-3 and either C/EBP-α or -β represses E2F target genes [6,7].
205	junb → tf_ink4a	1	[144]
206	c_abl → tf_ink4a	1	[150]
207	cdc6 → repress_ink4a	1	[150]
208	c_myctrans → repress_ink4a	1	Myc interacts directly with Smad2 and Smad3 on p15 promoter [84].<br>Furthermore it induces bmi1 expression, which represses INK4b [151].
209	ets1_2 → tf_ink4a	1	[139]
210	tf_ink4a · $\overline{\text{repress\_ink4a}}$ → ink4a	1	
211	$\overline{\text{sp1rep}}$ · sp1 · smad · miz_1 · $\overline{\text{cdc6}}$ → ink4b	1	Smad complexed with Sp1 can induce expression of p15 if not repressed by cdc6 [150] but it also requires the binding of Miz-1 to the core promoter [84].<br>A complex containing Sp1 and HDAC can bind promoters of Sp1 target genes and repress their expression [72].
212	free_p21 → free_p21_t2	2	We include a time scale '2' here since, in contrast to the activation of p21, STAT3 activation only involves post-transcriptional changes.
213	il6r → jak	1	[152]
214	cyclin_d1 · $\overline{\text{cdk46}}$ → free_cycd1	2	cyclin D1 inhibition of STAT3 has been shown to be CDK4/6 independent [153], so we assume that only free cyclin D1 can inhibit STAT3.
215	jak · $\overline{\text{free\_p21\_t2}}$ · $\overline{\text{free\_cycd1}}$ → stat3	1	JAK activates STAT3 [154].<br>p21 inhibits STAT3 by binding [155].<br>Free cyclin D1 represses STAT3 activation [153].
216	c_src · $\overline{\text{free\_p21\_t2}}$ · $\overline{\text{free\_cycd1}}$ → stat3	1	Src activates STAT3 [18,156].<br>p21 inhibits STAT3 by binding [155].<br>Free cyclin D1 represses STAT3 activation [153].
217	mkk3_6 · $\overline{\text{erk}}$ · rhoa → mirk_dyrk1b	1	Mirk transcription is activated by a RhoA dependent mechanism and requires ERK inhibition [157].<br>Mirk/Dyrk1b kinase activaty is upregulated by MKK3/6 phosphorylation [148,158].<br>Furthermore, it can be sequestered by p38 [159]. However, this was shown in cancer cells with mutant p53 [158]. Hence, this effect was not included in our model.<br>Mirk expression seems to be cell cycle dependent (high in G_0/G_1, low (one tenth) in S phase and the rest of the cell cycle). In G_0/G_1, its expression is much higher than that of p38 so that one can assume that its inhibition by p38 can only occur in S phase, and not in G_0/G_1. Hence, we delayed this mechanism with a time scale '2' [157].

Continued on next page

№	Reaction	Time scale	References
218	pp2a_pr55 → phosphatase	1	Either PP1A or PP2A:PR55 (or both) is needed to dephosphorylate Raf allowing for its activation by Ras [160].<br>We however include this reaction in a OR gate instead of an ITT (Incomplete Truth Table) gate since in our model, proliferative signal leads to the inactivation of PP1A.
219	pp1a → phosphatase	1	Either PP1A or PP2A:PR55 (or both) is needed to dephosphorylate Raf allowing for its activation by Ras [160].<br>We however include this reaction in a OR gate instead of an ITT (Incomplete Truth Table) gate since in our model, proliferative signal leads to the inactivation of PP1A.
220	sos → ras	1	[132]
221	c_met → grb2	1	[161]
222	$\overline{\text{pkc}} \cdot \overline{\text{erk}}$ → rkip	1	PKC inhibits RKIP by phosphorylation on S153 [162] and so does ERK [163].
223	rsk → rsk_t2	2	
224	$\overline{\text{rsk\_t2}} \cdot \overline{\text{p27cyto\_t2}}$ · grb2 → sos	1	Grb2 activates Sos [132].<br>p27 inhibits Grb2 function by binding it after export to the cytoplasm [164].<br>Sos can be phosphorylation by RSK. This leads to the release of Sos from Grb2 [134]. A time scale of ‘2’ is used for the feedback with RSK because RSK is itself activated by ERK.
225	pp2a → pp2a_t2	2	
226	pp2a_pr61 → pr61_t2_b	2	
227	2 p27cyto → p27cyto_t2	2	
228	rkip → rkip_t2	2	
229	mekk · $\overline{\text{pp2a\_t2}}$ → mek	1	MEKK activates MEK [132]. PP2A dephosphorylates MEK1/2 [165].
230	ras · phosphatase → activ_raf	1	Raf needs to be dephosphorylated on S259 by PP1 and/or PP2A (hence the somehow included in the variable `phosphatase`) which seems to be allowed by Ras [160].
231	$\overline{\text{inhib\_raf}}$ · activ_raf → raf	1	
232	irs → grb2	1	[166]
233	$\overline{\text{pp2a\_t2}}$ · erk → inhib_raf	2	ERK phosphorylates and inhibits Raf but only after prior activation of Raf (hence the time scale ‘2’). PP2A can dephosphorylate ERK and allow for a new activation of Raf-1 [160].
234	c_src · phosphatase → activ_raf	1	src can activate Raf independently of Ras [109].<br>We suppose activation by c-src also requires previous dephosphorylation by PP1A and/or PP2A (included in `phosphatase`) like in the case of activation by Ras.
235	$\overline{\text{rkip\_t2}}$ · raf · $\overline{\text{pp2a\_t2}}$ → mek	1	Raf activates MEK [132].<br>PP2A dephosphorylates MEK1/2 [165].<br>RKIP prevents activation of MEK by ERK via binding of Raf and impeding their interaction [167].

Continued on next page

Nº	Reaction	Time scale	References
236	pkc · $\overline{\text{pp2a\_t2}}$ → mek	1	PKC activates MEK1 [168]. PP2A dephosphorylates MEK1/2 [165].
237	$\overline{\text{mkp}}$ · $\overline{\text{pr61\_t2\_b}}$ · mek → erk	1	PP2A can dephosphorylate ERK [109].<br>ERK translocation to the nucleus requires Rhoa-mediated actin rearrangement [169].<br>MKP dephosphorylates ERK [132,170].
238	egfr → grb2	1	[132]
239	jak → grb2	1	JAK1 activates Grb2-SOS [152,171,172].
240	tnfr → grb2	1	[173]
241	nf_kappa_b → trans_myc	1	[108,174]
242	c_jun → trans_myc	1	[174]
243	ets1_2 → trans_myc	1	[174]
244	sp1 · 2 e2f1_3a → trans_myc	1	[72]
245	free_p21 → repress_myc	2	The action of p21 on Myc seems to be a two step process: First it binds to the myc promoter and represses Myc expression [10]. Second it binds the Myc proteins directly and inhibits its transcriptional activity [90].<br>We give here a time scale of '2' because p21 must be free to inhibit myc and the sequestration of p21 requires cdk4/6, which requires Myc activity first. So the influence of p21 on Myc expression is present only after a possible expression of CDK4/6 by Myc.
246	2 p53 → repress_myc	1	Activated p53 represses c-Myc. Abrogation of this inhibition impedes cell cycle arrest by p53 [175].<br>Since p53 activation should provoke cell cycle arrest, we confer an activity of 2 to p53 so that only activation by stress leads to c-myc repression.
247	pp2a_pr61 → pr61_t2_c	2	
248	sp1rep → repress_myc	1	A complex (`sp1rep`) containing Sp1 and HDAC can bind promoters of Sp1 target genes and repress their expression [72].
249	c_ebp_rep → repress_myc	1	A complex (`c_ebp_rep`) formed by pRb, E2F1-3 and either C/EBP-α or -β represses E2F target genes [6,7].
250	radiations_uv → deg_myc	1	Myc is degraded after UV induced DNA damage independently of Skp2, Fbw7, the PI3K pathway, ATM/ATR, etc [104].
251	dna_damage → deg_myc	1	Myc is degraded after UV induced DNA damage independently of Skp2, Fbw7, the PI3K pathway, ATM/ATR, etc [104].
252	skp2 · pr61_t2_c → deg_myc	1	PP2A can destabilize Myc by dephosphorylating S62 (phosphorylated by ERK) and targets it to ubiquitination by Skp2 [176].<br>Since Skp2 is activated in late G_1, its activation occurs mainly downstream of Myc and we hence confer it a time scale '2'.

Continued on next page

Nº	Reaction	Time scale	References
253	skp2 · $\overline{\text{erk}}$ → deg_myc	1	Only unphosphorylated Myc (by ERK) is targeted by Skp2 for degradation [176].<br>Since Skp2 is activated in late G_1, its activation occurs mainly downstream of Myc and we hence confer it a time scale '2'.
254	$\overline{\text{akt}}$ · $\overline{\text{c\_myctrans}}$ → miz_1	1	Myc binds Miz-1 and blocks its transactivation [84].
255	$\overline{\text{akt}}$ · dna_damage → miz_1	1	Miz-1 is activated after DNA damage by dissociation of its inhibitor TOPBP1 [84]. Akt inhibits Miz-1 by phosphorylation on S428 [177].
256	$\overline{\text{akt}}$ · radiations_uv → miz_1	1	Miz-1 is activated after UV irradiation but can be inhibited by binding to Myc [84]. Akt inhibits Miz-1 by phosphorylation on S428 [177].
257	smad · e2f45repress → repress_myc	1	Myc is repressed by a complex formed of Smad3, E2F4 and p107 [178].
258	trans_myc · $\overline{\text{repress\_myc}}$ · $\overline{\text{deg\_myc}}$ → c_mycrep	1	Myc is regulated both transcriptionally and post-transcriptionally.
259	stat3 → trans_myc	1	[86]
260	fbw7 · gsk3b · pr61_t2_c · $\overline{\text{ras}}$ · erk → deg_myc	1	Degradation after ubiquitination by Fbw7 when Myc got phosphorylated by both ERK and GSK3-β [176]. But ubiquitination by Fbw7 can be prevented by Ras [30].<br>Dephosphorylation of S62 by PP2A is required for Fbw7 to recognize Myc [176].
261	$\overline{\text{arf}}$ · trans_myc · $\overline{\text{repress\_myc}}$ · $\overline{\text{deg\_myc}}$ → c_myctrans	1	ARF inhibits Myc transcriptional activity by binding but not its repressional potential [179].<br>Myc funtions are also regulated through its binding to Max. However Max is in stoichiometric excess to Myc and not rate-limiting for Myc activation [177].
262	oxidative_stress → ikk	1	[180]
263	$\overline{\text{beta\_trcp}}$ · $\overline{\text{rsk}}$ · $\overline{\text{ask}}$ · $\overline{\text{ikk}}$ · $\overline{\text{rock1}}$ → i_kappa_b	1	IκB is inhibited by IKK after phosphorylation [181].<br>ASK1 also inhibits IκB [169].<br>PP2A seems to play a role in the NF-κB pathway, but its contribution is not very clear [109].<br>ROCK inhibits IκB [182].<br>SCF$^{\beta-TrCP}$ ubiquitinates IκB and its overexpression has been shown to lead to activation of NF-κB [77].<br>RSK has also been shown to phosphorylate IκB [134].
264	akt → activ_ikk	1	Akt phosphorylates IKKα/β on S180 [108,141].
265	akt · $\overline{\text{2 p53}}$ · nfkbres · $\overline{\text{i\_kappa\_b}}$ → nf_kappa_b	1	IκB inhibits NF-κB by binding. Its removal allows NF-κB nuclear import [181].<br>Akt indirectly leads to the phosphorylation of the p65 subunit, which is necessary for NF-κB activation [183].<br>p53 inhibits NF-κB by binding: it sequestrates it in the cytoplasm and competes with its co-activator p300/CBP [145].

Continued on next page

Nº	Reaction	Time scale	References
266	nf_kappa_b → i_kappa_b	2	[184]
267	tnfr · pkc → activ_ikk	1	PKCζ activates IKK by phosphorylation. But for this it needs to be specifically located by p62, a protein activated by TNF-α [168].
268	$\overline{\text{p53}}$ · nfkbres · $\overline{\text{i\_kappa\_b}}$ · rock1 → nf_kappa_b	1	Activation of NF-κB by ROCK is a two step process: First, it inhibits IκB, second its phosphorylates the p65 subunit of NF-κB, which is necessary for induction of transcription [182].<br>p53 inhibits NF-κB by binding: it sequestrates it in the cytoplasm and competes with its co-activator p300/CBP [145].<br>IκB inhibits NF-κB by binding. Its removal allows NF-κB nuclear import [181].
269	radiations_uv → ikk	1	[180]
270	$\overline{\text{tao1\_3}}$ · tak1 → activ_ikk	1	TAK activates IKK but after osmotic stress, activation of IKK by TAK is prevented by the activation of TAO2 after osmotic stress [185].
271	activ_ikk · $\overline{\text{chk1\_2}}$ → ikk	1	Chk1 inhibits IKK [108].
272	tao1_3 → mkk4_7	1	[186]
273	tak1 → mkk4_7	1	[181]
274	ask → mkk4_7	1	[181]
275	tgfbr → mekk	1	[129]
276	rac → mekk	1	[125]
277	mekk → mkk4_7	1	[187]
278	p53 · $\overline{\text{ros}}$ → mkp	1	MKP expression is activated by p53 [188] and MKP phosphatase activity inhibited by Ros [181].
279	pp2a → pp2a_t2_d	2	
280	p21cyto → p21cyto_t2	2	
281	$\overline{\text{wip1}}$ · mkk3_6 → p38	1	p38 is phosphorylated by MKK [187] and dephosphorylated by Wip1 [189].
282	radiations_uv → ros	1	[190]
283	oxidative_stress → ros	1	[190]
284	ask → mkk3_6	1	[187]
285	mekk → mkk3_6	1	[187]
286	tak1 → mkk3_6	1	[187]
287	rac → ros	1	[169]
288	tnfr → ros	1	[181]
289	osmotic_stress → tak1	1	[185]
290	atm_atr → tao1_3	1	[186]
291	$\overline{\text{pp2a\_t2\_d}}$ · $\overline{\text{p21cyto\_t2}}$ · $\overline{\text{mkp}}$ · mkk4_7 → jnk	1	MKK4/7 activates JNK by phosphorylation [181].<br>PP2A can dephosphorylate and inhibit JNK. Furthermore, it dephosphorylates MKK4, which prevents JNK activation but does not influence p38 activation [109,191].<br>Cytoplasmic p21 binds to JNK and prevents its activation by upstream kinases [90].

Continued on next page

Nº	Reaction	Time scale	References
292	ros · $\overline{\text{p21cyto\_t2}}$ → ask	1	ASK1 is activated by ROS [181] but also inhibited by cytoplasmic p21 [192].<br>We use a time scale '2' for the inhibition by p21 to take the gene regulation involved in p21 regulation.
293	tnfr · $\overline{\text{p38}}$ → tak1	1	TNF-α activates TAK1 but phosphorylation of the TAB1 subunit of TAK on S423, T431 and S438 by p38 leads to its inhibition [193].
294	tgfbr · $\overline{\text{p38}}$ → tak1	1	TGF-β activates TAK1 [129] but phosphorylation of the TAB1 subunit of TAK on S423, T431 and S438 by p38 leads to its inhibition [193].
295	c_src → mkk4_7	1	[191]
296	$\overline{\text{wip1}}$ · jnk → p53	1	JNK can phosphorylate S15, T81 and S34 [188]. Wip1 dephosphorylates S15, which leads to a decrease in p53 levels [110].
297	dmp1 → trans_arf	1	[194]
298	$\overline{\text{mdm2}}$ · $\overline{\text{c\_jun}}$ → 2 p53	1	MDM2 ubiquitinates p53 and targets it for degradation [195].<br>Since MDM2 first requires the activation of p53, we gives a time scale of '2' to this reaction.<br>c-jun represses p53 expression. We assume here that c-jun prevents the full activation of p53 (level '2') since it blocks p53 induced cell cycle arrest but keeps p53 in a basal activity level [67].
299	$\overline{\text{wip1}}$ · p38 → p53	1	p38 phosphorylates p53 on S15, S33, S46 [110,188,189] but it is dephosphorylated by Wip1 [110].
300	2 e2f1_3a → trans_arf	1	[189,194]
301	c_myctrans → trans_arf	1	Myc induces ARF transcription [179].
302	e2f45repress → repress_arf	1	[196]
303	p53 → repress_arf	1	[197]
304	arf → inh_mdm2	1	[198]
305	cdc6 → repress_arf	1	[150]
306	$\overline{\text{wip1}}$ · $\overline{\text{stat3}}$ · $\overline{\text{c\_jun}}$ · atm_atr → 2 p53	1	ATM/ATR phosphorylates p53 on S15, which inhibits the interaction of p53 with Mdm2 and results in p53 stabilization [105,199]. This site is dephosphorylated by Wip1 [110].<br>Repression by STAT3 partially inhibits p53 activation [18,200] but not completely as shown in [200]. Hence, we assume that p53 activity is only reduced to a level of '2'.<br>c-jun represses p53 expression. We assume here that c-jun prevents the full activation of p53 (level '2') since it blocks p53 induced cell cycle arrest but keeps p53 in a basal activity level [67].

Continued on next page

Nº	Reaction	Time scale	References
307	c_ebp_rep → repress_arf	1	A complex formed by pRb, E2F1-3 and either C/EBP-α or -β represses E2F target genes [6,7].
308	$\overline{\text{wip1}}$ · erk → p53	1	ERK1/2 phosphorylates p53 on S15 leading to its activation. In murine cells, it phosphorylates also T73 and T83 [188].<br>Wip1 dephosphorylates S15, which leads to a decrease in p53 levels [110].
309	p53 → mdm2	2	p53 induces expression of MDM2 [195].
310	$\overline{\text{inh\_mdm2}}$ → mdm2	1	Inhibition of MDM2 assures presence of p53 in the cytoplasm and hence a fast response to cellular damage [195].
311	2 p53 → wip1	2	Since this is observed after stress, we suppose p53 requires an activity of '2' corresponding to its activation after stress in our model [188,189].<br>A time scale '2' is used since Wip is only activated by p53, so its negative effect is only possible after prior activation by p53. This prevents Wip1 from inhibiting p53 activators.
312	c_abl · $\overline{\text{wip1}}$ · atm_atr → inh_mdm2	1	Inhibition of MDM2 (phosphorylation on S395) by ATM after DNA damage requires c-abl [188,201].<br>Wip1 can dephosphorylates S395 [202].
313	$\overline{\text{stat3}}$ · $\overline{\text{c\_jun}}$ · chk1_2 → 2 p53	1	Chk1/2 phosphorylates p53 on S20 and stabilizes it [105]. Repression by STAT3 partially inhibits p53 activation [18,200] but not completely as shown in [200]. Hence, we assume that this effect can only reduce p53 activity to a level of '2'.<br>c-jun represses p53 expression. We assume here that c-jun prevents the full activation of p53 (level '2') since it blocks p53 induced cell cycle arrest but keeps p53 in a basal activity level [67].
314	$\overline{\text{wip1}}$ · trans_arf · $\overline{\text{repress\_arf}}$ → arf	1	Wip1 suppresses Arf levels [110].
315	akt → mdm2	1	Akt phosphorylation on S166 and S188 allows for its nuclear import and stabilization [195,203].<br>MDM2 has also been shown to be phosphorylated by cyclin A:CDK2 on T216, which attenuates its activity [204]. This site is also dephosphorylated by PP2:PR61. Since cyclin A:CDK2 is not part of the model, this interaction will not be included.
316	$\overline{\text{e2f1\_3a}}$ · junb · c_jun · cycd123_cdk46 → dmp1	1	E2F1-3 has been shown to repress dmp1 expression. Furthermore, induction by Ras depends on c-Jun and JunB, and its transcriptional activity on the Arf promoter is dependent on cyclin D:CDK46 [194] .
317	egfr → activ_pi3k	1	PI3K is activated by growth factors [103].
318	$\overline{\text{tsc}}$ → rheb	1	[111]
319	rheb → mtor	1	[111]
320	irs · $\overline{\text{erk2\_t2}}$ → activ_pi3k	1	PI3K is activated by the insulin receptor [103,166]. But association of ERK2 to IRS-1 prevents PI3K activation [205].

Continued on next page

Nº	Reaction	Time scale	References
321	atm_atr → gsk3b	1	ATR stimulates GSK3β activity after UV irradiation [78].
322	c_met → activ_pi3k	1	PI3K is activated by growth factors [103].
323	pdk1 · erk → rsk	1	[142]
324	ras → activ_pi3k	1	[132]
325	radiations_uv → pkc	1	[191]
326	erk → erk2_t2	2	
327	tnfr → activ_pi3k	1	TNF-α induces PI3K activation in human hepatocytes [146].
328	iex_1 → iex1_t2	2	
329	pp2a_pr55 → pr55_t2	2	
330	pp2a_pr61 → pr61_t2	2	
331	pten → pten_t2	2	
332	p53 → pten	1	[195]
333	rsk → mtor	1	[142]
334	actin → activ_pi3k	2	Welch *et al.* [126] describe the upstream activity of Rac on PI3K and argues that it occurs upon prior activation of PI3K, which provides a positive feedback loop. This was later confirmed by Inoue *et al.* [206] who showed that PI3K activation is amplified by the combined action of actin (activated by Rac, itself activated by PI3K) and another PI3K (Rac-independent) pathway. Hence, since PI3K has to be activated first, we confer a time scale '2' to this reaction.
335	rock1 → pten	1	[207]
336	$\overline{\text{iex1\_t2}} \cdot \text{activ\_pi3k} \rightarrow \text{pi3k}$	1	IEX-1 inhibits PI3K activation [146].
337	$\overline{\text{rsk}} \cdot \overline{\text{akt}} \rightarrow \text{tsc}$	1	TSC is inhibited by RSK [142] and by Akt [111].
338	pip3 · pdk1 → pkc	1	Full activation of PKC requires direct interaction with both PIP3 and PDK1 [168].
339	$\overline{\text{pten\_t2}} \cdot \text{pi3k} \rightarrow \text{pip3}$	1	The catalytic subunit (p110) of PI3K phosphorylates PIP2 to generate PIP3 which is dephosphorylated by PTEN [208].
340	$\overline{\text{rsk}} \cdot \overline{\text{p70s6k}} \cdot \overline{\text{akt}} \rightarrow \text{gsk3b}$	1	Akt inhibits GSK3β by phosphorylation [132]. RSK and S6K has been shown to phosphorylate and inhibit GSK3 [134,209].
341	$\overline{\text{pr61\_t2}} \cdot \overline{\text{pr55\_t2}} \cdot \text{pip3} \cdot \text{pdk1} \cdot \text{rac} \rightarrow \text{akt}$	1	Akt is phosphorylated by PDK1 after activation of PIP3 by PI3K [210]. PP2A(PR55 and PR61) dephosphorylates Akt [109,111,211,212]. Rac activity is needed as scaffold to induce Akt activation through PAK [213]. However, knockdown of PAK inhibited growth factor-induced phosphorylation of Akt suggesting that PAK is important for effective activation of Akt in growth factor-stimulated cells. So we embedded Rac in this AND gate so that Rac and PIP3 are necessary for full Akt activation.

Continued on next page

№	Reaction	Time scale	References
342	$\overline{\text{pr55\_t2}}$ · pkc · pdk1 · mtor → p70s6k	1	mTOR activates S6K by phosphorylation. But binding of PDK1 and PKC(δ or ζ) is also necessary for full activation of S6K [112].<br>PP2A activated by TGF-β was shown to dephosphorylate S6K and to decrease its activity [129].
343	atm_atr → pp1a	1	[214,215]
344	$\overline{\text{2 cyce\_cdk2}}$ → pp1a	1	[216]
345	tgfbr · pp2ac · $\overline{\text{atm\_atr}}$ → pp2a_pr55	1	TGF-β activates PR55 [129].<br>ATM activation leads to the dissociation of PR55 and PP2Ac [109].
346	pp2ac · erk → pp2a_pr48	1	ERK activates PP2A and a decrease in MEK phosphorylation is observed [165]. Since this study didn't discover which subunit of PP2A gets activated, we assume that all of them are activated.
347	pp2a_pr48 → pp2a	1	
348	pp2ac · $\overline{\text{inhib\_pr61}}$ · chk1_2 → pp2a_pr61	1	Chk1/2 activates the B56δ of PP2A after DNA damage [217].
349	pp2a_pr55 → pp2a	1	
350	pp2a_pr61 → pp2a	1	
351	pp2a_pr70 → pp2a	1	
352	pp2ac · erk · $\overline{\text{atm\_atr}}$ → pp2a_pr55	1	ERK activates PP2A and a decrease in MEK phosphorylation is observed [165]. Since this study didn't discover which subunit of PP2A gets activated, we assume that all of them are activated.<br>ATM activation leads to the dissociation of PR55 and PP2Ac [109].
353	pp2ac · $\overline{\text{inhib\_pr61}}$ · erk → pp2a_pr61	1	ERK activates PP2A and a decrease in MEK phosphorylation is observed [165]. Hence, we confer a time scale '2' to MAPK dephosphorylation by PP2A. Since this study didn't discover which subunit of PP2A gets activated, we assume here that all of them are activated.
354	pp2ac · erk → pp2a_pr70	1	ERK activates PP2A and a decrease in MEK phosphorylation is observed [165]. Since this study didn't discover which subunit of PP2A becomes activated, we assume here that all of them are activated.
355	pp2ac · ros → pp2a_pr70	1	ROS activate the PR70 subunit of PP2A [218].
356	erk · iex_1 → inhib_pr61	1	ERK in complex with the product of the early gene IEX-1 phosphorylates PR61 leading to its dissociation from PP2Ac [109,212].
357	egfr · pp2ac · $\overline{\text{atm\_atr}}$ → pp2a_pr55	1	EGF and PDGF were found to recruit PR55 to the membrane in order to activate Raf [219].
358	c_met · pp2ac · $\overline{\text{atm\_atr}}$ → pp2a_pr55	1	EGF and PDGF were found to recruit PR55 to the membrane in order to activate Raf [219].<br>Since this seems to be a general property of growth factors, we assume that c-Met is also capable of this effect.

Continued on next page

Nº	Reaction	Time scale	References
359	$\overline{\text{2 cyce\_cdk2}}$ → prb	1	Fully activated cyclin E:CDK2 complexes (level '3') inhibit full pRb activity after phosphorylation [35].<br>p21 induces pRb degradation after DNA damage [220]. This could lead to E2F1-3 activation and probably apoptosis, a process that we don't include in this model.
360	prb → prb2	1	This reaction is only used for validation purposes (hepatocyte-specific scenarios). prb2 is equal to '1' when prb > '0', and '0' otherwise.
361	2 e2f1_3a → e2f_res	1	Free E2F1-3 (level '2') induces its own expression [221].
362	pp1a → prb	1	PP1 dephosphorylate all pRb sites but cyclin D/Cdk4 sites are dephosphorylated first [222,223].
363	chk1_2 → 2 prb	1	[224]
364	2 e2f1_3a → p107_130	2	It has been shown that E2F1-3 binds the p107 promoter [225].
365	foxo → p107_130	1	FoxO3a and FoxO4 upregulates p130 protein expression [226].
366	$\overline{\text{cycd123\_cdk46}}$ → 2 prb	1	Phosphorylation by cyclin D:CDK4/6 complexes partially inhibits pRb activity [35].<br>It prevents binding of HDAC to the pRb:e2F complex and removes active repression of genes like CCNE1. In addition, this first phosphorylation seems to facilitate further phosphorylation by cyclin E:CDK2 complexes [227].
367	pp2a_pr70 → 2 prb	2	PP2A:PR70 interacts directly with and dephosphorylates pRb [218].<br>This dephosphorylation seems to counterbalance CDK-induced phosphorylation [228].
368	$\overline{\text{p107\_130}}$ · e2f3b45_res → e2f4	1	E2F4-5 is released by inhibition of p107/130 proteins after phosphorylation [21].
369	p107_130 · hdac1 · e2f3b45_res → e2f45repress	1	A complex formed by p107/130, E2F3b4-5 is bound by HDAC1 to repress E2F genes [227].
370	2 prb · hdac1 · e2f3b45_res → e2f45repress	1	A complex formed by p107/130, E2F3b4-5 is bound by HDAC1 to repress E2F genes [227].<br>pRb can substitute for p107/130 in p107/130 deficient cells [21].<br>We assume here that pRb must be unphosphorylated for this to happen.
371	$\overline{\text{cycd123\_cdk46}}$ · $\overline{\text{2}}$ $\overline{\text{cyce\_cdk2}}$ → p107_130	1	cyclin D:Cdk4/6 and cyclin E:Cdk2 complexes phosphorylate and inhibit p107/130 [21].
372	$\overline{\text{2 prb}}$ · e2f_res · $\overline{\text{gsk3b}}$ · $\overline{\text{arf}}$ · c_myctrans · $\overline{\text{c\_ebp\_alpha}}$ · $\overline{\text{free\_p21}}$ → e2f1_3a	1	Myc induces expression of E2F1/2 [52]. Furthermore, Myc seems required to allow the interaction of the E2F1 protein with the E2F gene promoters [229].<br>p21 directly binds and inhibits E2F transcription factors [230].<br>García-Alvarez *et al.* [231] showed also that GSK3β binds to E2F1 and inhibits its activity. HDAC actively represses genes expressed by E2F such as CCND3 [232], so we included this effect here. But this active repression is not directly included on the cyclin D3 state itself.<br>Arf inhibits E2F1-3 transcriptional activity by binding independently of its ability to induce E2F1-3 degradation [233,234] .<br>Binding of C/EBPα to E2F represses its transcriptional activity [235].<br>Here, pRb must have a value <2 for E2F to reach the value '1'. This simulates the partial inhibition of pRb by cyclin D:CDK4/6 complexes and the partial activation of E2F, allowing the expression of the CCNE gene.

Continued on next page

Nº	Reaction	Time scale	References
373	$\overline{\text{prb}}$ · e2f_res · $\overline{\text{gsk3b}}$ · $\overline{\text{arf}}$ · c_myctrans · $\overline{\text{c\_ebp\_alpha}}$ · $\overline{\text{free\_p21}}$ → 2 e2f1_3a	1	Myc induces expression of E2F1/2 [52]. Furthermore, Myc seems required to allow the interaction of the E2F1 protein with the E2F gene promoters [229].<br>p21 directly binds and inhibits E2F transcription factors [230].<br>García-Alvarez *et al.* [231] showed also that GSK3β binds to E2F1 and inhibits its activity. HDAC actively represses genes expressed by E2F such as CCND3 [232], so we included this effect here. But this active repression is not directly included on the cyclin D3 state itself.<br>Arf inhibits E2F1-3 transcriptional activity by binding independently of its ability to induce E2F1-3 degradation [233,234].<br>Binding of C/EBPα to E2F represses its transcriptional activity [235]<br>Here, pRb must be completely inhibited for E2F to become fully activated (value '2'). This simulates the full inhibition of pRb by cyclin E:CDK2 complexes and the full activation of E2F, allowing the expression of S phase genes.
374	chk1_2 → activ_foxo_b	1	CDK2 phosphorylates and inhibits FoxO. Chk12 induces dephosphorylation of the sites phosphorylated by CDK2 [37].<br>We assume that cyclin E:CDK2 must be fully active, hence the activity level of '2'.
375	$\overline{\text{2 e2\_t2}}$ → activ_foxo_b	1	CDK2 phosphorylates and inhibits FoxO. Chk12 induces dephosphorylation of the sites phosphorylated by CDK2 [37].<br>We assume that cyclin E:CDK2 must be fully active, hence the activity level of '2'.
376	smad → activ_foxo_a	1	[236]
377	serum_starvation → activ_foxo_a	1	[237]
378	oxidative_stress → activ_foxo_a	1	[237]
379	$\overline{\text{akt}}$ → activ_foxo_a	1	After phosphorylation by Akt, FoxO can either be degraded by Skp2 or simply exported to the cytoplasm by 14-3-3 proteins [37]. Hence, Skp2 does not appear in the equation.
380	cycd123_cdk46 → d46_t2	2	
381	cyce_cdk2 → e2_t2	2	
382	activ_foxo_b · activ_foxo_a → foxo	1	We here use 2 dummies activated by OR gates and bound together by a AND gate to avoid the accumulation of AND gates.
383	tgfbr · $\overline{\text{e2\_t2}}$ · $\overline{\text{d46\_t2}}$ · $\overline{\text{erk}}$ → smad	1	Smad is activated by TGF-β signalling [236].<br>Smad3 is phosphorylated by Cdk4 and Cdk2, which decreases its transcriptional activity [238].<br>ERK inhibits Smad1 and Smad2/3 [236].

Continued on next page

№	Reaction	Time scale	References
384	sp1res · prb → sp1	1	Reports indicate that pRb regulates Sp1 DNA binding activity [138].
385	sp1res · $\overline{\text{2 mdm2}}$ → sp1	1	MDM2 interferes with Sp1 DNA binding but this happens only with amplified levels of MDM2, not during normal growth [239].
386	sp1res · 2 prb · hdac1 · e2f_res · $\overline{\text{p53}}$ · $\overline{\text{2 mdm2}}$ → sp1rep	1	Sp1 is often seen as repressive when bound to E2F1 and pRb. We assume here that this is attributed to binding to HDAC, which is mediated by pRb when it has a level '2' [21].<br>Binding of either free E2F1 or p53 is supposed to disrupt direct binding of HDAC to Sp1 [72]. But since E2F is free only if pRb has a level '0', it is modelled the same way as the previous assertion.<br>Indeed we would need 2prb+!2e2f, which is equivalent to 2prb+prb. This can be summarized by 2prb.<br>MDM2 interferes with Sp1 DNA binding but this happens only with amplified levels of MDM2, not during normal growth [239].
387	pp2a_pr55 → pr55_t2_b	2	
388	egfr → activ_abl	1	EGFR can activate c-abl via interaction between its SH2 domain and phosphorylated tyrosine residues of the receptor [124].
389	atm_atr → activ_abl	1	ATM activates c-abl by phosphorylation on S465 after DNA damage [201,240].
390	dna_damage → lyn	1	[241]
391	c_src → activ_abl	1	Reports indicate that Src activates c-abl after growth factor activation (demonstrated with PDGF) [124].
392	tgfbr · pi3k → activ_abl	1	TGF-β activates c-abl. This activation needs both PAK2 and PI3K activities [124].
393	$\overline{\text{pr55\_t2\_b}}$ · pkc → c_src	1	Phosphorylation of S12 by PKC after UV irradiation leads to c-Src activation. This phosphorylation is reversed by PP2A:PR55 [191].<br>Also growth factors such as EGF and HGF activate c-Src [242], but c-Src activity seems to be reversed by PP2A [241]. However, since PKC is activated by both EGF and HGF, we suppose that this activation is induced by PKC as well.
394	activ_abl · $\overline{\text{2 prb}}$ → c_abl	1	Unphosphorylated pRb inhibits c-abl by binding. It can form ternary complexes with E2F. This inhibition is released after pRb phosphorylation on S807/811 by cyclin D:CDK4/6 complexes [243].
395	radiations_uv → crl4_cdt2	1	[75]
396	2 e2f1_3a → trans_skp2	1	E2F activates the expression of Skp2 [244].
397	nf_kappa_b → trans_skp2	1	[108]
398	3 cyce_cdk2 → stabil_skp2	1	cyclin E:Cdk2 can phosphorylate Skp2 on S64 and prevents its ubiquitination by APC(cdh1) [245].
399	mdia → trans_skp2	1	mDia activates Skp2 expression [123].
400	$\overline{\text{apc\_cdh1}}$ → stabil_skp2	1	APC(Cdh1) can ubiquitinate Skp2 [77,245].
401	2 e2f1_3a · $\overline{\text{c\_ebp\_rep}}$ → emi1	1	E2F1-3 expresses Emi1 [246].<br>A complex formed by pRb, E2F1-3 and either C/EBP-α or -β represses E2F target genes [6,7].<br>Emi1 is also targeted by SCF$^{\beta-TrCP}$ for ubiquitination, but this event requires prior phosphorylation by active Cdk1 and thus it can only happen starting in early mitosis [9]. Hence, we don't include any interaction between SCF$^{\beta-TrCP}$ and Emi1.

Continued on next page

№	Reaction	Time scale	References
402	jnk → beta_trcp	1	[180]
403	radiations_uv → skp2	1	UV irradiation is sufficient to increase Skp2 levels [75].
404	$\overline{\text{emi1}}$ · $\overline{\text{ros}}$ → apc_cdh1	1	Emi1 inhibits APC^{cdh1} [246].<br>ROS promotes the dissociation of APC^{cdh1} to allow for G_1/S progression [247].
405	trans_skp2 · stabil_skp2 · $\overline{2}$ $\overline{\text{prb}}$ · $\overline{\text{c\_ebp\_rep}}$ → skp2	1	pRb inhibits Skp2 by direct interaction [248,249].<br>We assume that only unphosphorylated pRb interacts with Skp2 although this has not been proven.<br>A complex formed by pRb, E2F1-3 and either C/EBP-α or -β represses E2F target genes [6,7].
406	p70s6k → s6k_t2	2	
407	egf_tgf_alpha → egfr	1	[132]
408	pkc → pkc_t2	2	
409	jnk → jnk_t2	2	
410	hgf → c_met	1	[161]
411	p53 → hgf	2	p53 binds the promoters of the EGF receptor, TGF-α and HGF [115].
412	tgf_beta → tgfbr	1	TGF-β binds the TGF-β receptor of type I to recruit and activate by phosphorylation the receptor of type II [250].
413	il_6 → il6r	1	[251]
414	tnf_alpha → tnfr	1	[252]
415	p53 → egf_tgf_alpha	2	p53 binds the promoters of the EGF receptor, TGF-α and HGF.<br>TGF-α induction has been shown in primary rat hepatocytes stimulated with HGF (medium contains also Insulin among other things) [115].
416	$\overline{\text{s6k\_t2}}$ · $\overline{\text{pkc\_t2}}$ · $\overline{\text{jnk\_t2}}$ · insulin → irs	1	IRS is activated by Insulin [166].<br>S6K as well as JNK and PKCζ inhibit IRS by phosphorylation [205].

Table S2.1 – Documentation of the equations contained in the original model.

2.2 Species contained in the original model (excluding dummies) and their default values

Species Name	Default value	Notes
tnf_alpha	0	Tumor Necrosis Factor-α (TNF-α).
tnfr		Tumor Necrosis Factor Receptor (TNFR).
tgf_beta	0	Transforming Growth Factor β (TGF-β).
tgfbr		Phosphorylated TGF-β receptor of type II bound to receptor of type I.
irs		Insulin receptor substrate.
insulin	0	Insulin.
il_6	0	Interleukin-6 (IL-6).
il6r		Interleukin 6 receptor (IL6R), also known as CD126 (Cluster of Differentiation 126).<br>The receptor for IL-6 consists of a heterodimeric complex of two Ig-like containing proteins, the IL-6 specific chain gp80 (CD126), and gp130 (CD130) [251].

Continued on next page

Species Name	Default value	Notes
hgf	0	Hepatocyte Growth Factor (HGF).
egf_tgf_alpha	0	Epidermial Growth Factor (EGF).<br>Transfoming growth Factor α (TGF-α).
egfr		Epidermal growth factor receptor (EGFR).
c_met		c-Met (or hepatocyte growth factor receptor (HGFR)).
skp2		S-phase kinase-associated protein 2 (Skp2). Subunit of the ubiquitin-protein ligase complex SCF.
kpc	1	Kip1 ubiquitylation-Promoting Complex (KPC).<br>Seems constitutively active during the cell cycle [253].
fbxw8	1	F-box/WD repeat-containing protein 8 (FBXW8).
fbx4	1	F-box only protein 4 (FBXO4 or FBX4).
fbw7	1	F-box/WD repeat-containing protein 7 (FBXW7 or FBW7).
emi1		F-box only protein 5 (or FBXO5), also known as Emi1.
crl4_cdt2		CRL4^{Cdt2} E3 ligase (composed of the Cul4A/B, DDB1 [damage-specific DNA-binding protein-1], and the DCAF subunit Cdt2) [75].
beta_trcp		F-box/WD repeat-containing protein 1A also known as β-TrCP, subunit of the ubiquitin-protein ligase complex SCF.
apc_cdh1		Cdc20homologue-1 (Cdh1 also known as Hct1 or Fizzy-related), substrate adaptor protein of the anaphase-promoting complex (APC).
lyn		Lyn kinase, member of the Src family of tyrosine kinases.
c_src		c-src (cellular-src, short for sarcoma), member of the Src family of tyrosine kinases.<br>c-src is activated for example through PDGF receptor activation.
c_abl		c-Abl tyrosine kinase.<br>Is not a member of the src family.
sp1res	1	Reservoir of Sp1.
sp1rep		Repressive form of the Sp1 transcription factor bound to E2F1 and pRb.
sp1		Sp1 is a basal transcription factor [254]. Although cell cycle proteins increases or decreases its abundance, it seems that this regulation is dependent on the Sp1 protein itself [255,256]. Hence, we do not include this regulation.
smad		Complex of the common-mediator Smad (Smad4) and the receptor-regulated Smads (Smad1, 2, or 3).
foxo		Family of Forkhead box protein O (FoxO1, FoxO3a, FoxO4).
prb		Retinoblastoma protein (RB or RB1 or pRb). Member of the pocket protein family.
p107_130		Retinoblastoma-like protein 1 (p107).<br>Retinoblastoma-like protein 2 (p130).<br>Members of the pocket protein family.
hdac1	1	Histone deacetylase 1 (HDAC1).
e2f_res	1	Reservoir of transcription factors E2F1, E2F2, E2F3a.
e2f4		E2F transcription factors 3b, 4 and 5, p107/p130-binding.
e2f3b45_res	1	Reservoir of E2F transcription factors 3b, 4 and 5.
e2f1_3a		Transcription factors E2F1, E2F2, E2F3a.
pp2a_pr70		PP2A activated by the regulatory subunit PR70.
pp2a_pr61		PP2A activated by the regulatory subunit PR61 (also known as B56 or B').

Continued on next page

Species Name	Default value	Notes
pp2a_pr55		PP2A activated by the regulatory subunit PR55 (also known as B55 or B).
pp2a_pr48		PP2A activated by the regulatory subunit PR48 (also known as B”).
pp2ac	1	PP2A consists of a dimeric core enzyme composed of the structural A and catalytic C subunits, and a regulatory B subunit. `pp2ac` refers here to the complex formed by the catalytic C subunit together with the structural A subunit.
pp2a		Protein phosphatase 2 (PP2), also known as PP2A.
pp1a		Phosphoprotein phosphatase 1α (PP1a).
tsc		Tuberous sclerosis protein 1 and 2 (TSC1/2).
rsk		p90 ribosomal s6 kinase.
rheb		GTP-binding protein Rheb also known as Ras homolog enriched in brain (RHEB).
pten		Phosphatase and tensin homolog (PTEN).
pkc		Protein kinase C (PKC). PKC here stands for PKCη and ζ, the only two isoforms that were shown to influence the cell cycle [81].
pip3		Phosphatidylinositol (3,4,5)-trisphosphate (PtdIns(3,4,5)P3) (PIP3).
pi3k		Phosphatidylinositol 3-kinase (PI3K).
pdk1	1	Pyruvate dehydrogenase kinase, isozyme 1 (PDK1).
p70s6k		p70S6 kinase (p70S6K).
mtor		Mammalian target of rapamycin (mTOR).
gsk3b		Glycogen synthase kinase 3β (GSK-3β).
akt		RAC serine/threonine-protein kinase, also known as Akt or protein kinase B (PKB).
wip1		Wip1 phosphatase.
p53		Protein 53 (or tumor protein 53 or simply p53). We could confer an activity of ‘2’ to p53 after stress (leading to G_1 arrest [188]) so that p53 can adopt an activity of ‘1’ after growth factor stimulation leading to proliferation as observed in [257].
mdm2		Murine double minute (mdm2).
dmp1		Dentin matrix acidic phosphoprotein 1.
arf		p14^{ARF} is an alternate reading frame (ARF) product of the CDKN2A (p16) locus.
tao1_3		TAO kinase 1 and 3.
tak1		Mitogen-activated protein kinase kinase kinase 7, also known as TAK1.
ros		Reactive oxygen species (ROS).
p38		p38 mitogen-activated protein kinase.
mkp		MAP kinase phosphatase.
mkk4_7		Mitogen-activated protein kinase kinase 4 and 7.
mkk3_6		Mitogen-activated protein kinase kinase 3 and 6.
mekk		Mitogen-activated protein kinase kinase kinase.
jnk		c-Jun N-terminal kinase (JNK).
ask		Mitogen-activated protein kinase kinase kinase 5 (MAP3K5), also known as Apoptosis signal-regulating kinase 1 (ASK1).

Continued on next page

Species Name	Default value	Notes
nf_kappa_b		nuclear factor kappa-light-chain-enhancer of activated B cells (NF-κB).
nfkbres	1	Reservoir of NF-κB.
i_kappa_b		Nuclear factor of kappa light polypeptide gene enhancer in B-cells inhibitor, α (IκBα).
ikk		IκB kinase (IKK).
miz_1		Zinc finger and BTB domain-containing protein 17, also known as Miz-1.
c_myctrans		Transcriptionally active form of the Myc (c-Myc) transcription factor.
c_mycrep		Repressive form of the Myc (c-Myc) transcription factor.
sos		Son of Sevenless (SOS).
rkip		Raf Kinase Inhibitory Protein (RKIP).
ras		RAt Sarcoma (RAS).
raf		Proto-oncogene c-RAF.
mek		Mitogen-activated protein kinase kinase or MAP2K or MEK 1 and 2.
grb2		Growth factor receptor-bound protein 2 (Grb2).
erk		Extracellular-signal-regulated kinases 1 and 2.
mirk_dyrk1b		Dual specificity tyrosine-phosphorylation-regulated kinase 1B (Dyrk1B), also known as Mirk.<br>Mirk levels are high in G_0 and then diminish in G_1 [99].<br>Dyrk1A/B posses a NLS sequence and are predominantly nuclear, but can also be found in the cytoplasm [157].<br>Although its higher expression is found in the skeletal muscle, it is also found in the brain and the liver as well.
kis	1	Kinase interacting stathmin.
dyrk1a	0	Dual specificity tyrosine-phosphorylation-regulated kinase 1A.<br>Dyrk1A/B posses a NLS sequence and are predominantly nuclear but can also be found in the cytoplasm [157].<br>Found in the brain but was also proved to be expressed in the liver.
stat3		Signal transducer and activator of transcription 3.
jak		Janus kinase 2.
ink4c		Cyclin-dependent kinase inhibitor 2C, also known as inhibitor of CDK4 kinase (Ink4) or p18.
ink4b		Cyclin-dependent kinase inhibitor 2B, also known as inhibitor of CDK4 kinase (Ink4) or p15.
ink4a		Cyclin-dependent kinase inhibitor 2A, also known as inhibitor of CDK4 kinase (Ink4) or p16.
sap1		SRF accessory protein 1 (Sap-1) also known as ETS domain-containing protein Elk-4.
mef2c		MADS box transcription enhancer factor 2.
junb		JunB transcription factor.
ets1_2		(E-twenty six) (ETS) transcription factor 1 and 2.
elk1		E twenty-six (ETS)-like transcription factor 1 (Elk1).
c_jun		c-Jun transcription factor.
c_fos		c-fos transcription factor.
c_ebp_rep		A complex formed by pRb, E2F1-3 and either C/EBP-α or -β represses E2F target genes [6,7].

Continued on next page

Species Name	Default value	Notes
c_ebp_beta		CCAAT-enhancer-binding protein β.<br>Is highly expresssed in quiescent hepatocytes but unphosphorylated [136].<br>We refer here to the phosphorylated form of C/EBPβ.
c_ebp_alpha		CCAAT-enhancer-binding protein α.<br>Expressed at high level in the liver [136].
c_ebpbres	1	Reservoir of C/EBPβ.
creb		cAMP response element-binding transcription factor (CREB).
ap1		Here AP-1 refers to the complex c-fos/c-jun only.
stress_fiber_formation		Stress fiber formation.
rock1		Rho-associated, coiled-coil containing protein kinase 1 (ROCK1).
rhoa		Ras homolog gene family, member A (RhoA).
rac		Rac subfamily of the Rho family of GTPases.
mdia		Protein diaphanous homolog 1 (mDia1).
cell_adhesion		Cell adhesion.
actin		Actin filaments.
pcna		Proliferating Cell Nuclear Antigen (PCNA).
npat		Nuclear protein, ataxia-telangiectasia (NPAT).
mcm2_7		DNA replication licensing factor Minichromosome Maintenance (MCM) 2 and 7.
dna_replication		DNA replication.
dhfr		Dihydrofolate reductase (DHFR).
cell_growth		Cell growth.
cdc6		Cell Division Cycle 6 (CDC6).
serum_starvation	0	Serum starvation (deprivation of growth factors).
contact_inhibition	0	Contact inhibition.
radiations_uv	0	UV radiations.
oxidative_stress	0	Oxidative stress.
osmotic_stress	0	Osmotic stress.
dna_damage		DNA damage.
chk1_2		Checkpoint kinases 1 and 2.
atm_atr		Ataxia telangiectasia mutated (ATM) and Ataxia telangiectasia and Rad3 related (ATR).
crm1	1	Exportin 1.
p27res	1	Reservoir of p27.
p27nuc		Nuclear form of Cyclin-dependent kinase inhibitor 1B, also known as $p27^{Kip1}$.<br>p27 concentration is high in the nucleus during G_0 and contributes to the quiescent state [100].
p27cyto		Cytoplasmic form of Cyclin-dependent kinase inhibitor 1B, also known as $p27^{Kip1}$.
p27		Cyclin-dependent kinase inhibitor 1B, also known as $p27^{Kip1}$ (cytoplasmic or nuclear).
wisp39	1	WAF-1/CIP1 stabilizing protein 39
p21nuc		Nuclear form of cyclin-dependent kinase inhibitor 1, also known as $p21^{Cip1/Waf1}$.

Continued on next page

Species Name	Default value	Notes
p21cyto		Cytoplasmic form of cyclin-dependent kinase inhibitor 1, also known as p21$^{Cip1/Waf1}$.
p21		Cyclin-dependent kinase inhibitor 1, also known as p21$^{Cip1/Waf1}$ (cytoplasmic or nuclear).
cyclin_d3		Cyclin D3.
cyclin_d2		Cyclin D2.
cyclin_d1		Cyclin D1.
cycd123_cdk46		Active state of the complex formed by either cyclin D1, cyclin D2 or cyclin D3 with CDK4 or CDK6.
cdk46		Cyclin-dependent kinases 4 and 6.<br>This species corresponds to the presence of the kinase, not to the kinase activity.
cyclin_e1		Cyclin E1.
cyce_cdk2		Active state of the complex formed by cyclin E1 and CDK2.
cdk2		Cyclin-dependent kinase 2.<br>This species corresponds to the presence of the kinase, not to the kinase activity.
wee1		Wee1 kinase.
cdc25a		Cdc25A phosphatase.
cak	1	CDK-activating kinase. Constitutively active [258].

Table S2.2 – Species contained in the original model (excluding dummies) and their default values when applicable.

2.3 Interpretation of dummy names according to their suffix/prefix

Names containing	Meaning
trans, tf, exp	Gene expression (trans=transcription, tf=transcription factor, exp=expression).
activ	Activation.
inh, inhib	Inhibition.
deg	Degradation.
repress	Gene repression.
stabil	Protein stabilisation.
dephospho	Dephosphorylation.
phospho, phos	Phosphorylation.
transloc	Cytoplasmic translocation = Nuclear export.
ligase	Ubiquitin ligases used targeting a given species.
sequester	Sequestration of a protein by binding.
free	Free (unbound) state of a protein.
t(+number)	Phosphorylation on a given threonine site.
s(+number)	Phosphorylation on a given serine site.
t2	Species regulated in time scale 2.

Table S2.3 – Interpretation of dummy names according to their suffix/prefix.

2.4 Changes made to the original model as described in the paper

Iteration	Changes	Model changes	Agreement with literature scenarios
0	Original model.	—	60.00% correct<br>30.63% wrong<br>9.37% uncalculable
1	Removal of sp1rep dependence on prb.	Added:<br>$\overline{\text{2 prb}} \rightarrow \text{prb2\_sp1}$ (time scale '2')<br><br>Changed:<br>$\overline{\text{2 mdm2}} \cdot \text{p53} \cdot \text{e2f\_res} \cdot \text{hdac1} \cdot \text{2 prb} \cdot \text{sp1res} \rightarrow \text{sp1rep}$<br><br>into:<br>$\overline{\text{2 mdm2}} \cdot \text{p53} \cdot \text{e2f\_res} \cdot \text{hdac1} \cdot \overline{\text{prb2\_sp1}} \cdot \text{sp1res} \rightarrow \text{sp1rep}$	64.37% correct<br>35.63% wrong<br>0.00% uncalculable
2	cdk2, cdk46 and cyclin_d3 defined as inputs.	Set default value to 1 for cdk2, cdk46 and cyclin_d3.	71.25% correct<br>28.75% wrong<br>0.00% uncalculable
3	Insulin and TNFα can induce PI3K and MAPK activation only in combination.	Removed:<br>$\text{tnfr} \rightarrow \text{grb2}$<br>$\text{irs} \rightarrow \text{grb2}$<br>$\text{irs} \cdot \overline{\text{erk2\_t2}} \rightarrow \text{activ\_pi3k}$<br>$\text{tnfr} \rightarrow \text{activ\_pi3k}$<br><br>Added:<br>$\text{tnfr} \cdot \text{irs} \rightarrow \text{grb2}$<br>$\text{tnfr} \cdot \text{irs} \cdot \overline{\text{erk2\_t2}} \rightarrow \text{activ\_pi3k}$	76.25% correct<br>23.75% wrong<br>0.00% uncalculable

Table S2.4 – Changes made to the original model.

Doc. S3 Hepatocyte-specific scenarios for model validation

Nº	Cell type	Stimulation	Perturbation	Model inputs	Output	Comments	Ref.
1	primary rat hepatocytes	HGF (10 ng/mL)	None.	`hgf=1, all other receptors=0`	`DNA synthesis, p53=1`	TGFα is also synthesized.	[115]
2	primary rat hepatocytes	HGF (10 ng/mL)	p53 inhibition (pifithrin-α).	`p53=0, hgf=1, all other receptors=0`	`no DNA synthesis`	No difference with respect to the control is observed for a concentration of 8μM pifithrin-α. The result of this scenario contrasts with Arora *et al.* who showed that p53 inhibition in liver regeneration leads to an increased hepatocyte proliferation [259].	[115]
3	primary rat hepatocytes	HGF (10 ng/mL) + TGFα (40 ng/mL)	p53 inhibition (antisense oligonucleotide)	`p53=0, egf_tgf_alpha=1,hgf=1,all other receptors=0`	`DNA synthesis`	Full proliferation is recovered by adding TGFα (40 ng/mL) to the medium when p53 is inhibited by antisense oligonucleotide. From this and the previous experiment, one could conclude that p53 primary function is the sole synthesis of TGFα but it has been shown in other cell types that TGFα stimulation leads to DNA synthesis in p53 positive cells but fails in p53 negative cells.	[115]
4	primary rat hepatocytes	HGF (10 ng/mL) + Insulin (100 nM)	dominant-negative p53.	`p53=0, hgf=1, insulin=1, all other receptors=0`	`no DNA synthesis`		[32]
5	primary mouse [260] and rat [260,261] hepatocytes	EGF (10 ng/mL) [261] or TGFα (12 nM) [260]	None.	`egf_tgf_alpha=1, all other receptors = 0`	`no DNA synthesis, cyclin_d1=0, cyclin_d3=1, rsk=1`	C/EBPβ becomes phosphorylated by RSK but its activity can still be inhibited indirectly by Akt [137].	[260,261]
6	primary mouse and rat hepatocytes	TGFα (12 nM)	C/EBPβ-/-	`c_ebp_beta=0, egf_tgf_alpha=1, all other receptors = 0`	`no DNA synthesis`		[260]
7	primary rat hepatocytes	EGF (10 ng/mL) + Insulin (100 nM)	dominant-negative p53.	`p53=0, egf_tgf_alpha=1, insulin=1, all other receptors=0`	`no DNA synthesis, p21=0, prb>0, cyclin_d1=1, cyclin_e=1, cycd123_cdk46=0, cyce_cdk2=0, cdk2=1, cdk46=1`	Cdk4 and Cdk2 stay cytoplasmic. This worked also by replacing EGF by TGFα. Bellamy *et al.* found p53 inhibition having no effect on proliferation and even that the addition of TGFβ does not impede hepatocyte proliferation [262].	[32]
8	primary rat hepatocytes	EGF (10 ng/mL) + Insulin (100 nM)	p53 overexpression.	`p53=2, egf_tgf_alpha=1, insulin=1, all other receptors=0`	`no DNA synthesis`		[32]
9	primary rat hepatocytes	EGF (10 ng/mL) + Insulin (100 nM)	dominant-negative p53 + p21 ectopic expression.	`p53=0, tf_p21=1, egf_tgf_alpha=1, insulin=1, all other receptors=0`	`DNA synthesis, cyce_cdk2=1, cdk2=1, cycd123_cdk46=1, cdk46=1`	Cdk4 and Cdk2 are nuclear.	[32]
10	primary rat hepatocytes	EGF (10 ng/mL) + Insulin (100 nM)	p21 ectopic expression.	`tf_p21=1, egf_tgf_alpha=1, insulin=1, all other receptors=0`	`DNA synthesis, cycd123_cdk46=1, cyce_cdk2=1, cdk2=1`	Cdk4 and Cdk2 are nuclear.	[32]
11	primary rat hepatocytes	EGF (10 ng/mL [32,257, 261,263], 10 nM [264]) + Insulin (20 mUnits/mL [261,263], 100 nM [32, 257,264])	None.	`egf_tgf_alpha=1, insulin=1, all other receptors=0`	`DNA synthesis, cyclin_d1=1, cyclin_d3=1, cycd123_cdk46=1, pcna=1, p21=1, mek=1, erk=1, rsk=1, p53=1, cyclin_e=1, cyce_cdk2=1, prb=0, p27>0, cdk2=1, cdk46=1`	cyclin A is also expressed. $\frac{\mathrm{ERK_{cyto}}}{\mathrm{ERK_{nuc}}} = \frac{1}{4}$.	[32,257, 261,263,264]

Continued on next page

№	Cell type	Stimulation	Perturbation	Model inputs	Output	Comments	Ref.
12	primary rat hepatocytes	EGF (10 nM [264], 10 ng/mL [261]) + Insulin (100 nM [264], 20 mUnits/mL [261])	MEK1/2 inhibition (U0126 inhibitor [264]), MEK1 inhibition (PD98059 inhibitor [261])	mek=0, egf_tgf_alpha=1, insulin=1, all other receptors=0	no DNA synthesis, rsk=0, erk=0, cyclin_d1=0, cyclin_d3=1	Scheving *et al.* found induction of DNA synthesis when inhibiting MEK with 50μM of PD98059 before stimulation with 115nM Insulin and 20ng/mL EGF [265].	[261,264]
13	primary rat hepatocytes	EGF (10 ng/mL for all references) + Insulin (20mUnits/mL [261], 100 nM for all other references)	PI3K inhibitor (LY294002)	pi3k=0, egf_tgf_alpha=1, insulin=1, all other receptors=0	no DNA synthesis, cyclin_d1=0, cyclin_d3=1, cyclin_e=1, p53=0, p21=0, prb=2, cdk2=1, cyce_cdk2=0, cycd123_cdk46=0, cdk46=1	Inhibition of DNA synthesis confirmed by Scheving *et al.* [265]. Cdk4 and Cdk2 as well as cyclin D1 and cyclin E stay cytoplasmic.	[32,257,261]
14	primary rat hepatocytes	EGF (10 ng/mL) + Insulin (100 nM)	cyclin D1 expression.	cyclin_d1=1, egf_tgf_alpha=1, insulin=1, all other receptors=0	DNA synthesis, cyce_cdk2=1, cycd123_cdk46=1, prb=0,cdk2=1, cdk46=1		[257]
15	primary rat hepatocytes	EGF (10 ng/mL) + Insulin (100 nM)	cyclin D1 overexpressed and PI3K inhibited.	cyclin_d1=1, pi3k=0, egf_tgf_alpha=1, insulin=1, all other receptors=0	no DNA synthesis, cyce_cdk2=0, prb>0, cycd123_cdk46=0, cdk2=1, cdk46=1		[257]
16	primary rat hepatocytes	EGF (10 ng/mL) + Insulin (100 nM)	cdk4 inhibition.	cdk46=0, egf_tgf_alpha=1, insulin=1, all other receptors=0	no DNA synthesis, cdk2=1, cdk46=1, cyclin_e=1, cyce_cdk2=0, prb>0	In our model, Cdk4 and Cdk6 share the same variable so setting the variable cdk46 to '0' does not stimulate correctly this scenario. However since there is no visible compensation by Cdk6, it seems to be a good approximation. Cdk2 is cytoplasmic only and there is no cyclin E in the nucleus.	[257]
17	primary rat hepatocytes	EGF (10 ng/mL) + Insulin (100 nM)	p21 ectopic expression + dominant negative CDK4.	cdk46=0, tf_p21=1, egf_tgf_alpha=1, insulin=1, all other receptors=0	DNA synthesis	In our model, Cdk4 and Cdk6 share the same variable so setting the variable cdk46 to 0 does not stimulate correctly this scenario. However since there is no visible compensation by Cdk6, it seems to be a good approximation.	[266]
18	primary rat hepatocytes	EGF (10 ng/mL) + Insulin (20 mUnits/mL) + TGFβ (1 ng/mL)	None.	egf_tgf_alpha=1, insulin=1, tgf_beta=1, all other receptors=0	no DNA synthesis		[263]
19	primary rat hepatocytes	EGF (10 ng/mL) + Insulin (20 mUnits/mL) + TGFβ (1 ng/mL)	cyclin E and Skp2 adenovirus.	cyclin_e=1, skp2=1, egf_tgf_alpha=1, insulin=1, tgf_beta=1, all other receptors=0	DNA synthesis		[263]
20	primary rat hepatocytes	EGF (10 ng/mL) + Insulin (20 mUnits/mL) + TGFβ (1 ng/mL)	cyclin E adenovirus.	cyclin_e=1, egf_tgf_alpha=1, insulin=1, tgf_beta=1, all other receptors set to 0.	no DNA synthesis		[263]
21	primary rat hepatocytes	EGF (10 ng/mL) + Insulin (20 mUnits/mL) + TGFβ (1 ng/mL)	skp2 adenovirus.	skp2=1, egf_tgf_alpha=1, insulin=1, tgf_beta=1, all other receptors set to 0.	DNA synthesis		[263]
22	primary rat hepatocytes	Oxidative Stress (H_2O_2 1mM) + Insulin (20 mUnits/mL)	None.	oxidative_stress=1, insulin=1, all other receptors set to 0.	no DNA synthesis, rsk=1, mek=1, erk=1, egfr=1	Phosphorylated ERK is present in the cytoplasm but not in the nucleus. However in our model, nuclear translocation is not explicitly included,so phosphorylated ERK corresponds to the activated state of the species erk. RSK is nuclear.	[264]

Continued on next page

Nº	Cell type	Stimulation	Perturbation	Model inputs	Output	Comments	Ref.
23	primary rat hepatocytes	Oxidative Stress (H_2O_2 1mM) + Insulin (20 mUnits/mL)	MEK inhibition (U0126 inhibitor).	oxidative_stress=1, mek=0, insulin=1, all other receptors set to 0.	erk=0, rsk=0		[264]
24	primary rat hepatocytes	Oxidative Stress (H_2O_2 1mM) + Insulin (20 mUnits/mL)	EGF receptor inhibition.	oxidative_stress=1, egfr=0, insulin=1, all other receptors set to 0.	erk=1	ERK is activated by phosphorylation but cytoplasmic only. Since nuclear import is not included in our model, this state correspond to the species erk.	[264]
25	primary rat hepatocytes	Oxidative Stress (H_2O_2 1mM) + Insulin (20 mUnits/mL)	SRC inhibition (PP1 inhibitor, inhibits src family kinases).	oxidative_stress=1, c_src=0, lyn=0, insulin=1, all other receptors set to 0.	DNA synthesis		[264]
26	primary mouse hepatocytes	TNFα (1 ng/mL)	None.	tnf_alpha=1, all other receptors set to 0.	nf_kappa_b=1, i_kappa_b=0		[267]
27	primary mouse hepatocytes	TNFα (1 ng/mL)	GSK3 inhibition (SB216763)	gsk3b=0, tnf_alpha=1, all other receptors set to 0.	c_jun=1, creb=1, ap1=1		[267]
28	primary mouse hepatocytes	TNFα (1 ng/mL)	GSK3 inhibition (SB216763) and p38 inhibition (ML3403)	gsk3b=0, p38=0, tnf_alpha=1, all other receptors set to 0.	creb=0		[267]
29	primary rat hepatocytes	TNFα (25 ng/mL) + Insulin (0.1mmol/L)	GSK3 inhibition (LiCl)	gsk3b=0, tnf_alpha=1, insulin=1, all other receptors set to 0.	nf_kappa_b=0, ikk=1		[268]
30	primary mouse hepatocytes	TNFα (30 ng/mL) + Insulin (0.1 μM)	PI3K inhibition (LY-294002 inhibitor)	pi3k=0, tnf_alpha=1, insulin=1, all other receptors set to 0.	nf_kappa_b=0		[183]
31	primary mouse hepatocytes	TNFα (30 ng/mL) + Insulin (0.1 μM)	Akt constitutive activation (adenovirus expressing constitutively active Akt encoding an amino-terminal myristylation signal (AdmyrAkt)) + IκB activation (adenovirus)	akt=1,i_kappa_b=1, tnf_alpha=1, insulin=1, all other receptors set to 0.	nf_kappa_b=0		[183]
32	primary rat hepatocytes	Insulin (20 mUnits/mL [261], 100 nM [32,257])	None.	insulin=1, all other receptors=0	no DNA synthesis, cyclin_d1=0, cyclin_d3=1, cyclin_e=1, prb=2, cyce_cdk2=0, cycd123_cdk46=0	Wierød *et al.* argues that hepatocyte isolation and plating induce the priming phase [257]. But the presence of a Insulin in high concentration in the medium must also be taken into account. This paper also contains information about p21, p53, Cdk4 and Cdk2 but it is difficult to know if they are activated from these data.	[32,257,261]
33	primary rat hepatocytes	Insulin (100 nM)	cyclin D1 and p21 ectopic expression.	cyclin_d1=1, tf_p21=1, insulin=1, all other receptors=0	no DNA synthesis		[266]
34	primary rat hepatocytes	Insulin (100 nM)	PI3K inhibition	pi3k=0, insulin=1, all other receptors=1	no DNA synthesis, prb=2, cyclin_e=1, cyclin_d1=0		[257]
35	primary rat hepatocytes	Insulin (100 nM)	cyclin D1 expression.	cyclin_d1=1, insulin=1, all other receptors=0	no DNA synthesis, cyce_cdk2=0, prb>0, cdk2=1, cycd123_cdk46=0, cdk46=1	The contrary has been shown in intact rats [269] and also in cultured rat hepatocytes [270] but Wierød argues that the adenovirus transfection system used by the group of Albrecht might explain this discrepancy.	[257]

Continued on next page

Nº	Cell type	Stimulation	Perturbation	Model inputs	Output	Comments	Ref.
36	primary rat hepatocytes	None.	cyclin D1 adenovirus.	cyclin_d1=1, all receptors set to 0.	DNA synthesis, pcna=1, p21=1, p27=0, cdk2=1	cyclin A expressed.	[263,269, 270]
37	primary rat hepatocytes	None.	cyclin E and Skp2 adenovirus.	cyclin_e=1, skp2=1 and all receptors set to 0.	DNA synthesis, cyclin_d1=0, pcna=1, p21=1, p27=0, cdk2=1, cyce_cdk2=1	cyclin A is also expressed. But transfection system might be a problem.	[263]
38	primary rat hepatocytes	None.	cyclin E adenovirus.	cyclin_e=1, all receptors set to 0.	no DNA synthesis, pcna=0, p21=0, cyclin_d1=0, p27>0, cyce_cdk2=0, cdk2=1	cyclin A absent.	[263]
39	primary rat hepatocytes	None.	skp2 adenovirus	skp2=1, all receptors set to 0.	DNA synthesis, p21=0, cyclin_d1=0, p27=0, cdk2=1	cyclin A absent. PCNA level are higher than the unstimulated control but much lower than the control after EGF stimulation so we ignore this piece of information.	[263]
40	primary mouse and rat hepatocytes	None.	C/EBPβ phosphorylated	c_ebp_beta=1	DNA synthesis		[260]
41	primary mouse hepatocytes	None.	GSK3 inhibition (SB216763)	gsk3b=0, all receptors set to 0.	c_jun=1, creb=1, ap1=1		[267]
42	primary mouse hepatocytes	None.	p38 inhibition (ML3403)	p38=0,all receptors set to 0.	creb=0		[267]
43	primary mouse hepatocytes	None.	GSK3 inhibition (SB216763) and p38 inhibition (ML3403)	p38=0, gsk3b=0, all receptors set to 0.	creb=0		[267]
44	primary mouse [267] and rat [261,263] hepatocytes	None.	None.	all receptors set to 0	no DNA synthesis, pcna=0, p21=0, cyclin_d1=0, cyclin_d3=1, cdk46=1, cdk2=1, p27>0, i_kappa_b=1	cyclin A absent. Cdk4 is also absent but Cdk6 is present.	[261,263, 267]

Table S3.5 – Hepatocyte-specific scenarios for model validation.

Doc. S4 Western blot analysis of BX912 performance

For Western blot analysis primary mouse hepatocytes were used at sub-confluency. Cells were washed twice with PBS, received fresh cultivation medium and were pre-incubated with 15 μM BX912 or equal volumes of DMSO for 30 min prior to addition of 40 ng/ml rmHGF and further cultivation for 10 min. Cells were lysed in NP40 lysis buffer (1 % (v/v) NP40 (Roche Applied Sciences), 150 mM NaCl, 20 mM Tris pH 7.4, 10 mM NaF, 1 mM EDTA pH 8.0, 1 mM $ZnCl_2$ pH 4.0, 1 mM $MgCl_2$, 1 mM Na_3VO_4, 10 % (v/v) glycerol, 2 μg/ml aprotinin, 200 μg/ml AEBSF). Lysates were rotated for 20 min at 4 ℃ and centrifuged for 10 min at 14,000 rpm and 4 ℃ in a standard tabletop centrifuge. Supernatant was assayed for BCA to determine protein content according to the manufacturers instructions (Thermo Scientific). 30 μg of total protein were resolved on a 10 % SDS-PAGE and blotted on a PVDF membrane (Merck Millipore). Proteins were immobilized with Ponceau S solution and membranes were probed with α-pAkt (Thr308) (# 9275), α-pAkt (Ser473) (# 4058), α-Akt (# 9272), α-PDI (# 2446) (all Cell Signaling) as primary antibodies, and HRP-coupled -rabbit (GE Healthcare) as secondary antibody. Signals were detected using enhanced chemiluminescence (GE Healthcare) and a CCD camera-based Lumi Imager (Roche Applied Sciences).

Doc. S5 Statistical analysis of experimental data - p-values

	Condition	Reference	Adjusted p-value
Fig. 4A	TGFβ	unstimulated control	<0.0001
	TNFα	unstimulated control	1.0000
	Insulin	unstimulated control	0.0762
	IL-6	unstimulated control	0.0124
	EGF	unstimulated control	<0.0001
	HGF	unstimulated control	<0.0001
	IL-6 + Insulin	unstimulated control	0.0031
	IL-6 + TNFα	unstimulated control	0.0028
	TNFα + Insulin	unstimulated control	<0.0001
	TNFα + IL-6 + Insulin	unstimulated control	<0.0001
	HGF + Insulin	unstimulated control	<0.0001
	HGF + EGF	unstimulated control	<0.0001
	EGF + Insulin	unstimulated control	<0.0001
	HGF + TGFβ	unstimulated control	0.8092
	HGF + Insulin + TGFβ	unstimulated control	1.0000
Fig. 6A	DMSO + HGF	DMSO	<0.0001
	Akt VIII	DMSO	0.1596
	Akt VIII + HGF	DMSO	0.9847
	LY294002	DMSO	0.0042
	LY294002 + HGF	DMSO	0.9970
	U0126	DMSO	0.0429
	U0126 + HGF	DMSO	1.0000
Fig. 6B	DMSO + HGF	DMSO	<0.0001
	BX912	DMSO	0.0013
	BX912 + HGF	DMSO	1.0000
	PD 0332991	DMSO	1.0000
	PD 0332991 + HGF	DMSO	<0.0001
Fig. 6C	EtOH + HGF	EtOH	<0.0001
	LMB	EtOH	0.0181
	LMB + HGF	EtOH	0.999

Table S5.6 – p-values of statistical analysis for data presented in Fig. 4A and Fig. 6. To test statistical significance a mixed linear model was used (Materials and Methods). p-values for the comparison of log2 fold changes for all conditions and the corresponding reference measurement are displayed.

Doc. S6 Results of the structural sensitivity analysis

	1 reaction	2 reactions	3 reactions
ITT gate	**96.8%**, 100.0%, 73.5%, 88.3%	**93.8%**, 100.0%, 50.0%, 77.4%	**91.0%**, 100.0%, 44.1%, 67.4%
OR gate	**97.4%**, 100.0%, 79.4%, 89.2%	**95.1%**, 101.0%, 59.8%, 79.1%	**93.1%**, 101.0%, 51.0%, 69.9%
Removal	**95.8%**, 102.0%, 38.2%, 83.3%	**92.1%**, 103.9%, 27.4%, 69.0%	—

Table S6.7 – Structural sensitivity analysis. A single 'AND' gate of time scale '1' or combination of 2 or 3 was converted one by one into 'Incomplete Truth Table' or 'OR' gates. The predictions of the resulting models were compared to a dataset combining the literature dataset and our experimental dataset. Since the scenario where EGF alone is given as a stimulus is present in both datasets with different outcomes, we chose to include only the result of our own dataset, *i.e.* the induction of DNA synthesis. Both scenarios where TNFα is given as stimulus where combined as they are not contradictory. The model without structural perturbation is taken as reference. The value in **black** provides the average percentage of agreement with the reference model over all models. The percentages in red and green represent the maximal and minimal values respectively. Finally in blue we show the proportion of the model pool that agrees with at least 90% of the predictions of the reference model. The procedure was applied also after random removal of any single reaction of time scale '1' or a combination of 2 or 3. Conversion into 'ITT' or 'OR' gate was applied to the 128 'AND' gates of time scale '1' present in the model whereas removal concerned any of the 377 reactions of time scale '1'. Computation time for the removal of all combinations of 2 reactions of time scale '1' was 7 hours 49 minutes on a 8 core Intel® Xeon® CPU E5440 @ 2.83GHz with 6MB cache size and 8GB RAM with parallelized code. Analysis for removal of combinations of 3 reactions was not performed due to the time consuming computation.

References

1 Kholodenko BN (2000) Negative feedback and ultrasensitivity can bring about oscillations in the mitogen-activated protein kinase cascades. *Eur J Biochem* **267**, 1583–1588.

2 Iavarone A & Massagué J (1999) E2F and histone deacetylase mediate transforming growth factor beta repression of cdc25A during keratinocyte cell cycle arrest. *Mol Cell Biol* **19**, 916–922.

3 Ren B, Cam H, Takahashi Y, Volkert T, Terragni J, Young RA & Dynlacht BD (2002) E2F integrates cell cycle progression with DNA repair, replication, and G(2)/M checkpoints. *Genes Dev* **16**, 245–256.

4 Fernandez-Vidal A, Mazars A & Manenti S (2008) CDC25A: a rebel within the CDC25 phosphatases family? *Anticancer Agents Med Chem* **8**, 825–831.

5 Müller H & Helin K (2000) The E2F transcription factors: key regulators of cell proliferation. *Biochim Biophys Acta* **1470**, M1–12.

6 Johnson PF (2005) Molecular stop signs: regulation of cell-cycle arrest by C/EBP transcription factors. *J Cell Sci* **118**, 2545–2555.

7 Nerlov C (2007) The C/EBP family of transcription factors: a paradigm for interaction between gene expression and proliferation control. *Trends Cell Biol* **17**, 318–324.

8 Busino L, Chiesa M, Draetta GF & Donzelli M (2004) Cdc25A phosphatase: combinatorial phosphorylation, ubiquitylation and proteolysis. *Oncogene* **23**, 2050–2056.

9 Frescas D & Pagano M (2008) Deregulated proteolysis by the F-box proteins SKP2 and β-TrCP: tipping the scales of cancer. *Nat Rev Cancer* **8**, 438–449.

10 Vigneron A, Cherier J, Barré B, Gamelin E & Coqueret O (2006) The cell cycle inhibitor p21^{waf1} binds to the myc and cdc25A promoters upon DNA damage and induces transcriptional repression. *J Biol Chem* **281**, 34742–34750.

11 Rother K, Kirschner R, Sänger K, Böhlig L, Mössner J & Engeland K (2007) p53 downregulates expression of the G_1/S cell cycle phosphatase Cdc25A. *Oncogene* **26**, 1949–1953.

12 Kawasaki H, Komai K, Ouyang Z, Murata M, Hikasa M, Ohgiri M & Shiozawa S (2001) c-Fos/activator protein-1 transactivates wee1 kinase at G_1/S to inhibit premature mitosis in antigen-specific Th1 cells. *EMBO J* **20**, 4618–4627.

13 Kawasaki H, Komai K, Nakamura M, Yamamoto E, Ouyang Z, Nakashima T, Morisawa T, Hashiramoto A, Shiozawa K, Ishikawa H *et al.* (2003) Human wee1 kinase is directly transactivated by and increased in association with c-Fos/AP-1: rheumatoid synovial cells overexpressing these genes go into aberrant mitosis. *Oncogene* **22**, 6839–6844.

14 Katayama K, Fujita N & Tsuruo T (2005) Akt/protein kinase B-dependent phosphorylation and inactivation of WEE1Hu promote cell cycle progression at G2/M transition. *Mol Cell Biol* **25**, 5725–5737.

15 Chen S & Gardner DG (2004) Suppression of WEE1 and Stimulation of CDC25A Correlates with Endothelin-dependent Proliferation of Rat Aortic Smooth Muscle Cells. *The Journal of Biological Chemistry* **279**, 13755–13763.

16 Ray D, Terao Y, Nimbalkar D, Chu LH, Donzelli M, Tsutsui T, Zou X, Ghosh AK, Varga J, Draetta GF *et al.* (2005) Transforming growth factor β facilitates β-TrCP-mediated degradation of Cdc25A in a Smad3-dependent manner. *Mol Cell Biol* **25**, 3338–3347.

17 Karlsson-Rosenthal C & Millar JBA (2006) Cdc25: mechanisms of checkpoint inhibition and recovery. *Trends in Cell Biology* **16**, 285–292.

18 Barré B, Vigneron A, Perkins N, Roninson IB, Gamelin E & Coqueret O (2007) The STAT3 oncogene as a predictive marker of drug resistance. *Trends Mol Med* **13**, 4–11.

19 Goloudina A, Yamaguchi H, Chervyakova DB, Appella E, Fornace AJ & Bulavin DV (2003) Regulation of human Cdc25A stability by Serine 75 phosphorylation is not sufficient to activate a S phase checkpoint. *Cell Cycle* **2**, 473–478.

20 Barré B, Vigneron A & Coqueret O (2005) The STAT3 transcription factor is a target for the Myc and retinoblastoma proteins on the Cdc25A promoter. *J Biol Chem* **280**, 15673–15681.

21 Cobrinik D (2005) Pocket proteins and cell cycle control. *Oncogene* **24**, 2796–2809.

22 Xie RL, Gupta S, Miele A, Shiffman D, Stein JL, Stein GS & van Wijnen AJ (2003) The tumor suppressor interferon regulatory factor 1 interferes with SP1 activation to repress the human CDK2 promoter. *J Biol Chem* **278**, 26589–26596.

23 Pérez-Roger I, Solomon DL, Sewing A & Land H (1997) Myc activation of cyclin E/Cdk2 kinase involves induction of *cyclin E* gene transcription and inhibition of p27^{Kip1} binding to newly formed complexes. *Oncogene* **14**, 2373–2381.

24 Möröy T & Geisen C (2004) Cyclin E. *The International Journal of Biochemistry & Cell Biology* **36**, 1424–1439.

25 Shiyanov P, Bagchi S, Adami G, Kokontis J, Hay N, Arroyo M, Morozov A & Raychaudhuri P (1996) p21 Disrupts the interaction between cdk2 and the E2F-p130 complex. *Mol Cell Biol* **16**, 737–744.

26 He G, Siddik ZH, Huang Z, Wang R, Koomen J, Kobayashi R, Khokhar AR & Kuang J (2005) Induction of p21 by p53 following DNA damage inhibits both Cdk4 and Cdk2 activities. *Oncogene* **24**, 2929–2943.

27 Hoffmann I, Draetta G & Karsenti E (1994) Activation of the phosphatase activity of human cdc25A by a cdk2-cyclin E dependent phosphorylation at the G_1/S transition. *The EMBO Journal* **13**, 4302–4310.

28 Chen HC, Lee HC, Lin TY, Li WH & Chen BS (2004) Quantitative characterization of the transcriptional regulatory network in the yeast cell cycle. *Bioinformatics* **20**, 1914–1927.

29 Welcker M & Clurman BE (2008) FBW7 ubiquitin ligase: a tumour suppressor at the crossroads of cell division, growth and differentiation. *Nat Rev Cancer* **8**, 83–93.

30 Minella AC & Clurman BE (2005) Mechanisms of tumor suppression by the SCF^{Fbw7}. *Cell Cycle* **4**, 1356–1359.

31 Nakayama KI & Nakayama K (2005) Regulation of the cell cycle by SCF-type ubiquitin ligases. *Semin Cell Dev Biol* **16**, 323–333.

32 Wierød L, Rosseland CM, Lindeman B, Oksvold MP, Grøsvik H, Skarpen E & Huitfeldt HS (2008) Activation of the p53-p21(Cip1) pathway is required for CDK2 activation and S-phase entry in primary rat hepatocytes. *Oncogene* **27**, 2763–2771.

33 Sherr CJ & Roberts JM (1995) Inhibitors of mammalian G_1 cyclin-dependent kinases. *Genes Dev* **9**, 1149–1163.

34 Borriello A, Cucciolla V, Oliva A, Zappia V & Ragione FD (2007) $p27^{Kip1}$ metabolism: a fascinating labyrinth. *Cell Cycle* **6**, 1053–1061.

35 Sherr CJ (2000) The Pezcoller Lecture: Cancer Cell Cycles Revisited. *Cancer Res* **60**, 3689–3695.

36 Schmidt M, Fernandez de Mattos S, van der Horst A, Klompmaker R, Kops GJPL, Lam EWF, Burgering BMT & Medema RH (2002) Cell Cycle Inhibition by FoxO Forkhead Transcription Factors Involves Downregulation of Cyclin D. *Mol Cell Biol* **22**, 7842–7852.

37 Huang H & Tindall DJ (2007) Dynamic FoxO transcription factors. *J Cell Sci* **120**, 2479–2487.

38 Bakiri L, Lallemand D, Bossy-Wetzel E & Yaniv M (2000) Cell cycle-dependent variations in c-Jun and JunB phosphorylation: a role in the control of cyclin D1 expression. *The EMBO Journal* **19**, 2056–2068.

39 Albanese C, Johnson J, Watanabe G, Eklund N, Vu D, Arnold A & Pestell RG (1995) Transforming p21ras mutants and c-Ets-2 activate the cyclin D1 promoter through distinguishable regions. *J Biol Chem* **270**, 23589–23597.

40 Vermeulen K, Berneman ZN & Van Bockstaele DR (2003) Cell cycle and apoptosis. *Cell Prolif* **36**, 165–175.

41 Coqueret O (2002) Linking cyclins to transcriptional control. *Gene* **299**, 35–55.

42 Alao JP (2007) The regulation of cyclin D1 degradation: roles in cancer development and the potential for therapeutic invention. *Mol Cancer* **6**, 24.

43 Kida A, Kakihana K, Kotani S, Kurosu T & Miura O (2007) Glycogen synthase kinase-3β and p38 phosphorylate cyclin D2 on Thr280 to trigger its ubiquitin/proteasome-dependent degradation in hematopoietic cells. *Oncogene* **26**, 6630–6640.

44 Naderi S, Gutzkow KB, Låhne HU, Lefdal S, Ryves WJ, Harwood AJ & Blomhoff HK (2004) cAMP-induced degradation of cyclin D3 through association with GSK-3β. *J Cell Sci* **117**, 3769–3783.

45 Låhne HU, Kloster MM, Lefdal S, Blomhoff HK & Naderi S (2006) Degradation of cyclin D3 independent of Thr-283 phosphorylation. *Oncogene* **25**, 2468–2476.

46 Sherr CJ & Roberts JM (1999) CDK inhibitors: positive and negative regulators of G_1-phase progression. *Genes Dev* **13**, 1501–1512.

47 Watanabe G, Albanese C, Lee RJ, Reutens A, Vairo G, Henglein B & Pestell RG (1998) Inhibition of cyclin D1 kinase activity is associated with E2F-mediated inhibition of cyclin D1 promoter activity through E2F and Sp1. *Mol Cell Biol* **18**, 3212–3222.

48 Larrea MD, Liang J, Silva TD, Hong F, Shao SH, Han K, Dumont D & Slingerland JM (2008) Phosphorylation of p27Kip1 regulates assembly and activation of cyclin D1-Cdk4. *Mol Cell Biol* **28**, 6462–6472.

49 Barbash O, Lin DI & Diehl JA (2007) SCF Fbx4/alphaB-crystallin cyclin D1 ubiquitin ligase: a license to destroy. *Cell Div* **2**, 2.

50 Okabe H, Lee SH, Phuchareon J, Albertson DG, McCormick F & Tetsu O (2006) A critical role for FBXW8 and MAPK in cyclin D1 degradation and cancer cell proliferation. *PLoS ONE* **1**, e128.

51 Perez-Roger I, Kim SH, Griffiths B, Sewing A & Land H (1999) Cyclins D1 and D2 mediate myc-induced proliferation via sequestration of $p27^{Kip1}$ and $p21^{Cip1}$. *The EMBO Journal* **18**, 5310–5320.

52 Bernard S & Eilers M (2006) Control of Cell Proliferation and Growth by Myc Proteins. In *Cell Cycle Regulation*, *Results and Problems in Cell Differentiation*, vol. 42 (Kaldis P, ed.), pp. 329–342. Springer-Verlag.

53 Philipp A, Schneider A, Väsrik I, Finke K, Xiong Y, Beach D, Alitalo K & Eilers M (1994) Repression of cyclin D1: a novel function of MYC. *Mol Cell Biol* **14**, 4032–4043.

54 Casanovas O, Jaumot M, Paules AB, Agell N & Bachs O (2004) $p38^{SAPK2}$ phosphorylates cyclin D3 at Thr-283 and targets it for proteasomal degradation. *Oncogene* **23**, 7537–7544.

55 Casanovas O, Miró F, Estanyol JM, Itarte E, Agell N & Bachs O (2000) Osmotic stress regulates the stability of cyclin D1 in a $p38^{SAPK2}$-dependent manner. *J Biol Chem* **275**, 35091–35097.

56 Hermeking H, Rago C, Schuhmacher M, Li Q, Barrett JF, Obaya AJ, O'Connell BC, Mateyak MK, Tam W, Kohlhuber F *et al.* (2000) Identification of CDK4 as a target of c-MYC. *Proc Natl Acad Sci U S A* **97**, 2229–2234.

57 Cheng M, Olivier P, Diehl JA, Fero M, Roussel MF, Roberts JM & Sherr CJ (1999) The $p21^{Cip1}$ and $p27^{Kip1}$ CDK 'inhibitors' are essential activators of cyclin D-dependent kinases in murine fibroblasts. *The EMBO Journal* **18**, 1571–1583.

58 Bouchard C, Thieke K, Maier A, Saffrich R, Hanley-Hyde J, Ansorge W, Reed S, Sicinski P, Bartek J & Eilers M (1999) Direct induction of cyclin D2 by Myc contributes to cell cycle progression and sequestration of p27. *The EMBO Journal* **18**, 5321–5333.

59 Ma Y, Yuan J, Huang M, Jove R & Cress WD (2003) Regulation of the cyclin D3 promoter by E2F1. *J Biol Chem* **278**, 16770–16776.

60 Leslie K, Lang C, Devgan G, Azare J, Berishaj M, Gerald W, Kim YB, Paz K, Darnell JE, Albanese C *et al.* (2006) Cyclin D1 is transcriptionally regulated by and required for transformation by activated signal transducer and activator of transcription 3. *Cancer Res* **66**, 2544–2552.

61 Alt JR, Gladden AB & Diehl JA (2002) p21(Cip1) Promotes cyclin D1 nuclear accumulation via direct inhibition of nuclear export. *J Biol Chem* **277**, 8517–8523.

62 Blomen VA & Boonstra J (2007) Cell fate determination during G_1 phase progression. *Cell Mol Life Sci* **64**, 3084–3104.

63 Child ES & Mann DJ (2006) The intricacies of p21 phosphorylation: protein/protein interactions, subcellular localization and stability. *Cell Cycle* **5**, 1313–1319.

64 Abbas T & Dutta A (2009) p21 in cancer: intricate networks and multiple activities. *Nat Rev Cancer* **9**, 400–414.

65 Kim GY, Mercer SE, Ewton DZ, Yan Z, Jin K & Friedman E (2002) The stress-activated protein kinases p38 alpha and JNK1 stabilize p21(Cip1) by phosphorylation. *J Biol Chem* **277**, 29792–29802.

66 Lafarga V, Cuadrado A, Lopez de Silanes I, Bengoechea R, Fernandez-Capetillo O & Nebreda AR (2009) p38 Mitogen-activated protein kinase- and HuR-dependent stabilization of p21(Cip1) mRNA mediates the G(1)/S checkpoint. *Mol Cell Biol* **29**, 4341–4351.

67 Stepniak E, Ricci R, Eferl R, Sumara G, Sumara I, Rath M, Hui L & Wagner EF (2006) c-Jun/AP-1 controls liver regeneration by repressing p53/p21 and p38 MAPK activity. *Genes Dev* **20**, 2306–2314.

68 Gartel AL, Goufman E, Tevosian SG, Shih H, Yee AS & Tyner AL (1998) Activation and repression of p21(WAF1/CIP1) transcription by RB binding proteins. *Oncogene* **17**, 3463–3469.

69 Liberto M, Cobrinik D & Minden A (2002) Rho regulates p21(CIP1), cyclin D1, and checkpoint control in mammary epithelial cells. *Oncogene* **21**, 1590–1599.

70 Besson A, Assoian RK & Roberts JM (2004) Regulation of the cytoskeleton: an oncogenic function for CDK inhibitors? *Nat Rev Cancer* **4**, 948–955.

71 Gartel AL & Shchors K (2003) Mechanisms of c-myc-mediated transcriptional repression of growth arrest genes. *Exp Cell Res* **283**, 17–21.

72 Wierstra I (2008) Sp1: emerging roles–beyond constitutive activation of TATA-less housekeeping genes. *Biochem Biophys Res Commun* **372**, 1–13.

73 Hwang CY, Lee C & Kwon KS (2009) Extracellular signal-regulated kinase 2-dependent phosphorylation induces cytoplasmic localization and degradation of $p21^{Cip1}$. *Mol Cell Biol* **29**, 3379–3389.

74 Jascur T, Brickner H, Salles-Passador I, Barbier V, Khissiin AE, Smith B, Fotedar R & Fotedar A (2005) Regulation of p21(WAF1/CIP1) stability by WISp39, a Hsp90 binding TPR protein. *Mol Cell* **17**, 237–249.

75 Abbas T, Sivaprasad U, Terai K, Amador V, Pagano M & Dutta A (2008) PCNA-dependent regulation of p21 ubiquitylation and degradation via the $CRL4^{Cdt2}$ ubiquitin ligase complex. *Genes Dev* **22**, 2496–2506.

76 Bornstein G, Bloom J, Sitry-Shevah D, Nakayama K, Pagano M & Hershko A (2003) Role of the SCF^{Skp2} Ubiquitin Ligase in the Degradation of $p21^{Cip1}$ in S Phase. *The Journal of Biological Chemistry* **278**, 25752–25757.

77 Nakayama KI & Nakayama K (2006) Ubiquitin ligases: cell-cycle control and cancer. *Nat Rev Cancer* **6**, 369–381.

78 Lee JY, Yu SJ, Park YG, Kim J & Sohn J (2007) Glycogen synthase kinase 3β phosphorylates p21WAF1/CIP1 for proteasomal degradation after UV irradiation. *Mol Cell Biol* **27**, 3187–3198.

79 Takiguchi M (1998) The C/EBP family of transcription factors in the liver and other organs. *Int J Exp Pathol* **79**, 369–391.

80 Timchenko NA, Wilde M, Nakanishi M, Smith JR & Darlington GJ (1996) CCAAT/enhancer-binding protein alpha (C/EBPα) inhibits cell proliferation through the p21 (WAF-1/CIP-1/SDI-1) protein. *Genes Dev* **10**, 804–815.

81 Agell N, Jaumot M, Rodríguez-Vilarrupla A, Brun S, Abella N, Canela N, Estanyol JM & Bachs O (2006) The diverging roles of calmodulin and PKC in the regulation of p21 intracellular localization. *Cell Cycle* **5**, 3–6.

82 Seoane J, Le HV, Shen L, Anderson SA & Massagué J (2004) Integration of Smad and forkhead pathways in the control of neuroepithelial and glioblastoma cell proliferation. *Cell* **117**, 211–223.

83 Opitz OG & Rustgi AK (2000) Interaction between Sp1 and cell cycle regulatory proteins is important in transactivation of a differentiation-related gene. *Cancer Res* **60**, 2825–2830.

84 Wanzel M, Herold S & Eilers M (2003) Transcriptional repression by Myc. *Trends in Cell Biology* **13**, 146–150.

85 Koutsodontis G, Vasilaki E, Chou WC, Papakosta P & Kardassis D (2005) Physical and functional interactions between members of the tumour suppressor p53 and the Sp families of transcription factors: importance for the regulation of genes involved in cell-cycle arrest and apoptosis. *Biochem J* **389**, 443–455.

86 Barré B, Avril S & Coqueret O (2003) Opposite Regulation of Myc and $p21^{waf1}$ Transcription by STAT3 Proteins. *The Journal of Biological Chemistry* **278**, 2990–2996.

87 Giraud S, Hurlstone A, Avril S & Coqueret O (2004) Implication of BRG1 and cdk9 in the STAT3-mediated activation of the p21^{waf1} gene. *Oncogene* **23**, 7391–7398.

88 Bienvenu F, Barré B, Giraud S, Avril S & Coqueret O (2005) Transcriptional regulation by a DNA-associated form of cyclin D1. *Mol Biol Cell* **16**, 1850–1858.

89 Blagosklonny MV (2002) Are p27 and p21 cytoplasmic oncoproteins? *Cell Cycle* **1**, 391–393.

90 Besson A, Dowdy SF & Roberts JM (2008) CDK inhibitors: cell cycle regulators and beyond. *Dev Cell* **14**, 159–169.

91 Stewart ZA & Pietenpol JA (2001) p53 Signaling and Cell Cycle Checkpoints. *Chem Res Toxicol* **14**, 243–263.

92 Rodier G, Montagnoli A, Marcotullio LD, Coulombe P, Draetta GF, Pagano M & Meloche S (2001) p27 cytoplasmic localization is regulated by phosphorylation on Ser10 and is not a prerequisite for its proteolysis. *EMBO J* **20**, 6672–6682.

93 Fujita N, Sato S, Katayama K & Tsuruo T (2002) Akt-dependent phosphorylation of p27Kip1 promotes binding to 14-3-3 and cytoplasmic localization. *J Biol Chem* **277**, 28706–28713.

94 Ray A, James MK, Larochelle S, Fisher RP & Blain SW (2009) p27Kip1 inhibits cyclin D-cyclin-dependent kinase 4 by two independent modes. *Mol Cell Biol* **29**, 986–999.

95 Hulleman E & Boonstra J (2001) Regulation of G_1 phase progression by growth factors and the extracellular matrix. *Cell Mol Life Sci* **58**, 80–93.

96 Susaki E, Nakayama K & Nakayama KI (2007) Cyclin D2 translocates p27 out of the nucleus and promotes its degradation at the G_0-G_1 transition. *Mol Cell Biol* **27**, 4626–4640.

97 Susaki E & Nakayama KI (2007) Multiple mechanisms for p27(Kip1) translocation and degradation. *Cell Cycle* **6**, 3015–3020.

98 Chandramohan V, Mineva ND, Burke B, Jeay S, Wu M, Shen J, Yang W, Hann SR & Sonenshein GE (2008) c-Myc represses FOXO3a-mediated transcription of the gene encoding the p27(Kip1) cyclin dependent kinase inhibitor. *J Cell Biochem* **104**, 2091–2106.

99 Deng X, Mercer SE, Shah S, Ewton DZ & Friedman E (2004) The cyclin-dependent kinase inhibitor p27Kip1 is stabilized in G_0 by Mirk/dyrk1B kinase. *J Biol Chem* **279**, 22498–22504.

100 Vervoorts J & Lüscher B (2008) Post-translational regulation of the tumor suppressor p27(KIP1). *Cell Mol Life Sci* **65**, 3255–3264.

101 Wang C, Hou X, Mohapatra S, Ma Y, Cress WD, Pledger WJ & Chen J (2005) Activation of p27^{Kip1} Expression by E2F1. A negative feedback mechanism. *The Journal of Biological Chemistry* **280**, 12339–12343.

102 Larrea MD, Wander SA & Slingerland JM (2009) p27 as Jekyll and Hyde: regulation of cell cycle and cell motility. *Cell Cycle* **8**, 3455–3461.

103 Greer EL & Brunet A (2005) FOXO transcription factors at the interface between longevity and tumor suppression. *Oncogene* **24**, 7410–7425.

104 Britton S, Salles B & Calsou P (2008) c-MYC protein is degraded in response to UV irradiation. *Cell Cycle* **7**, 63–70.

105 Niida H & Nakanishi M (2006) DNA damage checkpoints in mammals. *Mutagenesis* **21**, 3–9.

106 Berkovich E & Ginsberg D (2003) ATM is a target for positive regulation by E2F-1. *Oncogene* **22**, 161–167.

107 Lee KH, Choi EY, Kim MK, Hyun MS, Jang BI, Kim TN, Kim SW, Song SK, Kim JH & Kim JR (2006) Regulation of hepatocyte growth factor-mediated urokinase plasminogen activator secretion by MEK/ERK activation in human stomach cancer cell lines. *Exp Mol Med* **38**, 27–35.

108 Barré B & Perkins ND (2007) A cell cycle regulatory network controlling NF-κB subunit activity and function. *EMBO J* **26**, 4841–4855.

109 Eichhorn PJA, Creyghton MP & Bernards R (2009) Protein phosphatase 2A regulatory subunits and cancer. *Biochim Biophys Acta* **1795**, 1–15.

110 Lu X, Nguyen TA, Moon SH, Darlington Y, Sommer M & Donehower LA (2008) The type 2C phosphatase Wip1: an oncogenic regulator of tumor suppressor and DNA damage response pathways. *Cancer Metastasis Reviews* **27**, 123–135.

111 Mamane Y, Petroulakis E, LeBacquer O & Sonenberg N (2006) mTOR, translation initiation and cancer. *Oncogene* **25**, 6416–6422.

112 Jastrzebski K, Hannan KM, Tchoubrieva EB, Hannan RD & Pearson RB (2007) Coordinate regulation of ribosome biogenesis and function by the ribosomal protein S6 kinase, a key mediator of mTOR function. *Growth Factors* **25**, 209–226.

113 Yan Z, DeGregori J, Shohet R, Leone G, Stillman B, Nevins JR & Williams RS (1998) Cdc6 is regulated by E2F and is essential for DNA replication in mammalian cells. *Proc Natl Acad Sci U S A* **95**, 3603–3608.

114 Yan Z, Fedorov SA, Mumby MC & Williams RS (2000) PR48, a novel regulatory subunit of protein phosphatase 2A, interacts with Cdc6 and modulates DNA replication in human cells. *Mol Cell Biol* **20**, 1021–1029.

115 Inoue Y, Tomiya T, Yanase M, Arai M, Ikeda H, Tejima K, Ogata I, Kimura S, Omata M & Fujiwara K (2002) p53 may positively regulate hepatocyte proliferation in rats. *Hepatology* **36**, 336–344.

116 Donjerkovic D & Scott DW (2000) Regulation of the G_1 phase of the mammalian cell cycle. *Cell Res* **10**, 1–16.

117 Fukami-Kobayashi J & Mitsui Y (1999) Cyclin D1 inhibits cell proliferation through binding to PCNA and cdk2. *Exp Cell Res* **246**, 338–347.

118 Prives C & Gottifredi V (2008) The p21 and PCNA partnership: a new twist for an old plot. *Cell Cycle* **7**, 3840–3846.

119 Woo RA & Poon RYC (2003) Cyclin-dependent kinases and S phase control in mammalian cells. *Cell Cycle* **2**, 316–324.

120 Zhao J (2004) Coordination of DNA synthesis and histone gene expression during normal cell cycle progression and after DNA damage. *Cell Cycle* **3**, 695–697.

121 Johnson DG, Schwarz JK, Cress WD & Nevins JR (1993) Expression of transcription factor E2F1 induces quiescent cells to enter S phase. *Nature* **365**, 349–352.

122 Park KK, Rue SW, Lee IS, Kim HC, Lee IK, Ahn JD, Kim HS, Yu TS, Kwak JY, Heintz NH *et al.* (2003) Modulation of Sp1-dependent transcription by a *cis*-acting E2F element in *dhfr* promoter. *Biochem Biophys Res Commun* **306**, 239–243.

123 Mammoto A, Huang S, Moore K, Oh P & Ingber DE (2004) Role of RhoA, mDia, and ROCK in cell shape-dependent control of the Skp2-p27kip1 pathway and the G1/S transition. *J Biol Chem* **279**, 26323–26330.

124 Sirvent A, Benistant C & Roche S (2008) Cytoplasmic signalling by the c-Abl tyrosine kinase in normal and cancer cells. *Biol Cell* **100**, 617–631.

125 Clarke N, Arenzana N, Hai T, Minden A & Prywes R (1998) Epidermal growth factor induction of the c-jun promoter by a Rac pathway. *Mol Cell Biol* **18**, 1065–1073.

126 Welch HCE, Coadwell WJ, Stephens LR & Hawkins PT (2003) Phosphoinositide 3-kinase-dependent activation of Rac. *FEBS Lett* **546**, 93–97.

127 Besson A, Gurian-West M, Schmidt A, Hall A & Roberts JM (2004) p27Kip1 modulates cell migration through the regulation of RhoA activation. *Genes Dev* **18**, 862–876.

128 Assoian RK (2004) Stopping and going with $p27^{kip1}$. *Dev Cell* **6**, 458–459.

129 Derynck R & Zhang YE (2003) Smad-dependent and Smad-independent pathways in TGF-β family signalling. *Nature* **425**, 577–584.

130 Shaulian E & Karin M (2001) AP-1 in cell proliferation and survival. *Oncogene* **20**, 2390–2400.

131 Hodge C, Liao J, Stofega M, Guan K, Carter-Su C & Schwartz J (1998) Growth hormone stimulates phosphorylation and activation of elk-1 and expression of c-fos, egr-1, and junB through activation of extracellular signal-regulated kinases 1 and 2. *J Biol Chem* **273**, 31327–31336.

132 Oda K, Matsuoka Y, Funahashi A & Kitano H (2005) A comprehensive pathway map of epidermal growth factor receptor signaling. *Mol Syst Biol* **1**, 1–17.

133 Buchwalter G, Gross C & Wasylyk B (2004) Ets ternary complex transcription factors. *Gene* **324**, 1–14.

134 Frödin M & Gammeltoft S (1999) Role and regulation of 90 kDa ribosomal S6 kinase (RSK) in signal transduction. *Mol Cell Endocrinol* **151**, 65–77.

135 Buck M & Chojkier M (2003) Signal transduction in the liver: C/EBPβ modulates cell proliferation and survival. *Hepatology* **37**, 731–738.

136 Ramji DP & Foka P (2002) CCAAT/enhancer-binding proteins: structure, function and regulation. *Biochem J* **365**, 561–575.

137 Guo S, Cichy SB, He X, Yang Q, Ragland M, Ghosh AK, Johnson PF & Unterman TG (2001) Insulin suppresses transactivation by CAAT/enhancer-binding proteins beta (C/EBPβ). Signaling to p300/CREB-binding protein by protein kinase B disrupts interaction with the major activation domain of C/EBPβ. *J Biol Chem* **276**, 8516–8523.

138 Chen LI, Nishinaka T, Kwan K, Kitabayashi I, Yokoyama K, Fu YHF, Grünwald S & Chiu R (1994) The retinoblastoma gene product RB stimulates Sp1-mediated transcription by liberating Sp1 from a negative regulator. *Mol Cell Biol* **14**, 4380–4389.

139 Chang F, Steelman LS, Lee JT, Shelton JG, Navolanic PM, Blalock WL, Franklin RA & McCubrey JA (2003) Signal transduction mediated by the Ras/Raf/MEK/ERK pathway from cytokine receptors to transcription factors: potential targeting for therapeutic intervention. *Leukemia* **17**, 1263–1293.

140 Flores-Delgado G, Liu CWY, Sposto R & Berndt N (2007) A limited screen for protein interactions reveals new roles for protein phosphatase 1 in cell cycle control and apoptosis. *J Proteome Res* **6**, 1165–1175.

141 Chang F, Lee JT, Navolanic PM, Steelman LS, Shelton JG, Blalock WL, Franklin RA & McCubrey JA (2003) Involvement of PI3K/Akt pathway in cell cycle progression, apoptosis, and neoplastic transformation: a target for cancer chemotherapy. *Leukemia* **17**, 590–603.

142 Anjum R & Blenis J (2008) The RSK family of kinases: emerging roles in cellular signalling. *Nat Rev Mol Cell Biol* **9**, 747–758.

143 Zarubin T & Han J (2005) Activation and signaling of the p38 MAP kinase pathway. *Cell Res* **15**, 11–18.

144 Piechaczyk M & Farràs R (2008) Regulation and function of JunB in cell proliferation. *Biochem Soc Trans* **36**, 864–867.

145 Wu MX (2003) Roles of the stress-induced gene IEX-1 in regulation of cell death and oncogenesis. *Apoptosis* **8**, 11–18.

146 Osawa Y, Nagaki M, Banno Y, Brenner DA, Nozawa Y, Moriwaki H & Nakashima S (2003) Expression of the NF-κB target gene X-ray-inducible immediate early response factor-1 short enhances TNF-α-induced hepatocyte apoptosis by inhibiting Akt activation. *J Immunol* **170**, 4053–4060.

147 López-Rovira T, Chalaux E, Rosa JL, Bartrons R & Ventura F (2000) Interaction and functional cooperation of NF-kappa B with Smads. Transcriptional regulation of the junB promoter. *J Biol Chem* **275**, 28937–28946.

148 Lim S, Jin K & Friedman E (2002) Mirk protein kinase is activated by MKK3 and functions as a transcriptional activator of HNF1α. *J Biol Chem* **277**, 25040–25046.

149 Pei XH & Xiong Y (2005) Biochemical and cellular mechanisms of mammalian CDK inhibitors: a few unresolved issues. *Oncogene* **24**, 2787–2795.

150 Kim WY & Sharpless NE (2006) The regulation of INK4/ARF in cancer and aging. *Cell* **127**, 265–275.

151 Guney I & Sedivy JM (2006) Cellular senescence, epigenetic switches and c-Myc. *Cell Cycle* **5**, 2319–2323.

152 Heinrich PC, Behrmann I, Haan S, Hermanns HM, Mller-Newen G & Schaper F (2003) Principles of interleukin (IL)-6-type cytokine signalling and its regulation. *Biochem J* **374**, 1–20.

153 Bienvenu F, Gascan H & Coqueret O (2001) Cyclin D1 represses STAT3 activation through a Cdk4-independent mechanism. *J Biol Chem* **276**, 16840–16847.

154 Akira S (1997) IL-6-regulated transcription factors. *The International Journal of Biochemistry & Cell Biology* **29**, 1401–1418.

155 Roninson IB (2002) Oncogenic functions of tumour suppressor p21(Waf1/Cip1/Sdi1): association with cell senescence and tumour-promoting activities of stromal fibroblasts. *Cancer Lett* **179**, 1–14.

156 Boureux A, Furstoss O, Simon V & Roche S (2005) Abl tyrosine kinase regulates a Rac/JNK and a Rac/Nox pathway for DNA synthesis and Myc expression induced by growth factors. *J Cell Sci* **118**, 3717–3726.

157 Mercer SE & Friedman E (2006) Mirk/Dyrk1B: a multifunctional dual-specificity kinase involved in growth arrest, differentiation, and cell survival. *Cell Biochem Biophys* **45**, 303–315.

158 Friedman E (2007) Mirk/Dyrk1B in cancer. *J Cell Biochem* **102**, 274–279.

159 Lim S, Zou Y & Friedman E (2002) The transcriptional activator Mirk/Dyrk1B is sequestered by p38α/β MAP kinase. *J Biol Chem* **277**, 49438–49445.

160 Dhillon AS, von Kriegsheim A, Grindlay J & Kolch W (2007) Phosphatase and feedback regulation of Raf-1 signaling. *Cell Cycle* **6**, 3–7.

161 Trusolino L, Bertotti A & Comoglio PM (2010) MET signalling: principles and functions in development, organ regeneration and cancer. *Nat Rev Mol Cell Biol* **11**, 834–848.

162 Lorenz K, Lohse MJ & Quitterer U (2003) Protein kinase C switches the Raf kinase inhibitor from Raf-1 to GRK-2. *Nature* **426**, 574–579.

163 Shin SY, Rath O, Choo SM, Fee F, McFerran B, Kolch W & Cho KH (2009) Positive- and negative-feedback regulations coordinate the dynamic behavior of the Ras-Raf-MEK-ERK signal transduction pathway. *J Cell Sci* **122**, 425–435.

164 Moeller SJ, Head ED & Sheaff RJ (2003) $p27^{Kip1}$ inhibition of GRB2-SOS formation can regulate Ras activation. *Mol Cell Biol* **23**, 3735–3752.

165 Bae D & Ceryak S (2009) Raf-independent, PP2A-dependent MEK activation in response to ERK silencing. *Biochem Biophys Res Commun* **385**, 523–527.

166 Taniguchi CM, Emanuelli B & Kahn CR (2006) Critical nodes in signalling pathways: insights into insulin action. *Nat Rev Mol Cell Biol* **7**, 85–96.

167 Yeung K, Seitz T, Li S, Janosch P, McFerran B, Kaiser C, Fee F, Katsanakis KD, Rose DW, Mischak H *et al.* (1999) Suppression of Raf-1 kinase activity and MAP kinase signalling by RKIP. *Nature* **401**, 173–177.

168 Hirai T & Chida K (2003) Protein kinase Cζ (PKCζ): activation mechanisms and cellular functions. *J Biochem (Tokyo)* **133**, 1–7.

169 Brown JH, Del Re DP & Sussman MA (2006) The Rac and Rho hall of fame: a decade of hypertrophic signaling hits. *Circ Res* **98**, 730–742.

170 Masuda K, Katagiri C, Nomura M, Sato M, Kakumoto K, Akagi T, Kikuchi K, Tanuma N & Shima H (2010) MKP-7, a JNK phosphatase, blocks ERK-dependent gene activation by anchoring phosphorylated ERK in the cytoplasm. *Biochem Biophys Res Commun* **393**, 201–206.

171 Taub R (2004) Liver Regeneration: from Myth to Mechanism. *Nat Rev Mol Cell Biol* **5**, 836–847.

172 Singh A, Sharma R, Jayaraman A & Hahn J (2006) Mathematical Model Of IL-6 Signal Transduction In Hepatocytes. *Biotechnol Bioeng* **95**, 850–862.

173 Hildt E & Oess S (1999) Identification of Grb2 as a novel binding partner of tumor necrosis factor (TNF) receptor I. *J Exp Med* **189**, 1707–1714.

174 Wierstra I & Alves J (2008) The c-myc promoter: still MysterY and Challenge. *Adv Cancer Res* **99**, 113–333.

175 Ho JSL, Ma W, Mao DYL & Benchimol S (2005) p53-Dependent transcriptional repression of c-myc is required for G_1 cell cycle arrest. *Mol Cell Biol* **25**, 7423–7431.

176 Amati B (2004) Myc degradation: dancing with ubiquitin ligases. *Proc Natl Acad Sci U S A* **101**, 8843–8844.

177 Adhikary S & Eilers M (2005) Transcriptional Regulation and Transformation by Myc Proteins. *Nat Rev Mol Cell Biol* **6**, 635–645.

178 Chen CR, Kang Y, Siegel PM & Massagué J (2002) E2F4/5 and p107 as Smad cofactors linking the TGFbeta receptor to c-myc repression. *Cell* **110**, 19–32.

179 Cleveland JL & Sherr CJ (2004) Antagonism of Myc functions by Arf. *Cancer Cell* **6**, 309–311.

180 Spiegelman VS, Stavropoulos P, Latres E, Pagano M, Ronai Z, Slaga TJ & Fuchs SY (2001) Induction of β-transducin repeat-containing protein by JNK signaling and its role in the activation of NF-κB. *J Biol Chem* **276**, 27152–27158.

181 Wullaert A, Heyninck K & Beyaert R (2006) Mechanisms of crosstalk between TNF-induced NF-κB and JNK activation in hepatocytes. *Biochem Pharmacol* **72**, 1090–1101.

182 Anwar KN, Fazal F, Malik AB & Rahman A (2004) RhoA/Rho-associated kinase pathway selectively regulates thrombin-induced intercellular adhesion molecule-1 expression in endothelial cells via activation of IκB kinase β and phosphorylation of RelA/p65. *J Immunol* **173**, 6965–6972.

183 Hatano E & Brenner DA (2001) Akt protects mouse hepatocytes from TNF-α- and Fas-mediated apoptosis through NF-κB activation. *Am J Physiol Gastrointest Liver Physiol* **281**, G1357–G1368.

184 Witt J, Barisic S, Schumann E, Allgower F, Sawodny O, Sauter T & Kulms D (2009) Mechanism of PP2A-mediated IKKβ dephosphorylation: a systems biological approach. *BMC Syst Biol* **3**, 71.

185 HuangFu WC, Omori E, Akira S, Matsumoto K & Ninomiya-Tsuji J (2006) Osmotic stress activates the TAK1-JNK pathway while blocking TAK1-mediated NF-κB activation: TAO2 regulates TAK1 pathways. *J Biol Chem* **281**, 28802–28810.

186 Raman M, Earnest S, Zhang K, Zhao Y & Cobb MH (2007) TAO kinases mediate activation of p38 in response to DNA damage. *EMBO J* **26**, 2005–2014.

187 Weston CR & Davis RJ (2002) The JNK signal transduction pathway. *Current Opinion in Genetics & Development* **12**, 14–21.

188 Wu GS (2004) The functional interactions between the p53 and MAPK signaling pathways. *Cancer Biol Ther* **3**, 156–161.

189 Harris SL & Levine AJ (2005) The p53 pathway: positive and negative feedback loops. *Oncogene* **24**, 2899–2908.

190 Hattori K, Naguro I, Runchel C & Ichijo H (2009) The roles of ASK family proteins in stress responses and diseases. *Cell Communication and Signaling* **7**, 9.

191 Eichhorn PJA, Creyghton MP, Wilhelmsen K, van Dam H & Bernards R (2007) A RNA interference screen identifies the protein phosphatase 2A subunit PR55gamma as a stress-sensitive inhibitor of c-SRC. *PLoS Genet* **3**, e218.

192 Asada M, Yamada T, Ichijo H, Delia D, Miyazono K, Fukumuro K & Mizutani S (1999) Apoptosis inhibitory activity of cytoplasmic p21(Cip1/WAF1) in monocytic differentiation. *EMBO J* **18**, 1223–1234.

193 Cheung PCF, Campbell DG, Nebreda AR & Cohen P (2003) Feedback control of the protein kinase TAK1 by SAPK2a/p38α. *EMBO J* **22**, 5793–5805.

194 Inoue K, Mallakin A & Frazier DP (2007) Dmp1 and tumor suppression. *Oncogene* **26**, 4329–4335.

195 Mayo LD & Donner DB (2002) The PTEN, Mdm2, p53 tumor suppressor-oncoprotein network. *Trends Biochem Sci* **27**, 462–467.

196 Aslanian A, Iaquinta PJ, Verona R & Lees JA (2004) Repression of the Arf tumor suppressor by E2F3 is required for normal cell cycle kinetics. *Genes & Development* **18**, 1413–1422.

197 Sherr CJ (2006) Divorcing ARF and p53: an unsettled case. *Nat Rev Cancer* **6**, 663–673.

198 Lu X, Nguyen TA, Zhang X & Donehower LA (2008) The Wip1 phosphatase and Mdm2: cracking the "Wip" on p53 stability. *Cell Cycle* **7**, 164–168.

199 Kruse JP & Gu W (2009) Modes of p53 regulation. *Cell* **137**, 609–622.

200 Niu G, Wright KL, Ma Y, Wright GM, Huang M, Irby R, Briggs J, Karras J, Cress WD, Pardoll D *et al.* (2005) Role of Stat3 in regulating p53 expression and function. *Mol Cell Biol* **25**, 7432–7440.

201 Levav-Cohen Y, Goldberg Z, Zuckerman V, Grossman T, Haupt S & Haupt Y (2005) C-Abl as a modulator of p53. *Biochem Biophys Res Commun* **331**, 737–749.

202 Lu X, Ma O, Nguyen TA, Jones SN, Oren M & Donehower LA (2007) The Wip1 Phosphatase acts as a gatekeeper in the p53-Mdm2 autoregulatory loop. *Cancer Cell* **12**, 342–354.

203 Feng J, Tamaskovic R, Yang Z, Brazil DP, Merlo A, Hess D & Hemmings BA (2004) Stabilization of Mdm2 via decreased ubiquitination is mediated by protein kinase B/Akt-dependent phosphorylation. *Journal of Biochemical Chemistry* **279**, 35510–35517.

204 Zhang T & Prives C (2001) Cyclin a-CDK phosphorylation regulates MDM2 protein interactions. *J Biol Chem* **276**, 29702–29710.

205 Harrington LS, Findlay GM & Lamb RF (2005) Restraining PI3K: mTOR signalling goes back to the membrane. *Trends Biochem Sci* **30**, 35–42.

206 Inoue T & Meyer T (2008) Synthetic activation of endogenous PI3K and Rac identifies an AND-gate switch for cell polarization and migration. *PLoS One* **3**, e3068.

207 Meili R, Sasaki AT & Firtel RA (2005) Rho Rocks PTEN. *Nat Cell Biol* **7**, 334–335.

208 Chalhoub N & Baker SJ (2009) PTEN and the PI3-kinase pathway in cancer. *Annual Review of Pathology* **4**, 127–150.

209 Sutherland C, Leighton IA & Cohen P (1993) Inactivation of glycogen synthase kinase-3β by phosphorylation: new kinase connections in insulin and growth-factor signalling. *Biochem J* **296**, 15–19.

210 Scheid MP & Woodgett JR (2003) Unravelling the activation mechanisms of protein kinase B/Akt. *FEBS Lett* **546**, 108–112.

211 Ugi S, Imamura T, Maegawa H, Egawa K, Yoshizaki T, Shi K, Obata T, Ebina Y, Kashiwagi A & Olefsky JM (2004) Protein phosphatase 2A negatively regulates insulin's metabolic signaling pathway by inhibiting Akt (protein kinase B) activity in 3T3-L1 adipocytes. *Mol Cell Biol* **24**, 8778–8789.

212 Rocher G, Letourneux C, Lenormand P & Porteu F (2007) Inhibition of B56-containing protein phosphatase 2As by the early response gene IEX-1 leads to control of Akt activity. *J Biol Chem* **282**, 5468–5477.

213 Higuchi M, Onishi K, Kikuchi C & Gotoh Y (2008) Scaffolding function of PAK in the PDK1-Akt pathway. *Nat Cell Biol* **10**, 1356–1364.

214 Guo CY, Brautigan DL & Larner JM (2002) Ionizing radiation activates nuclear protein phosphatase-1 by ATM-dependent dephosphorylation. *J Biol Chem* **277**, 41756–41761.

215 Tang X, Hui ZG, Cui XL, Garg R, Kastan MB & Xu B (2008) A novel ATM-dependent pathway regulates protein phosphatase 1 in response to DNA damage. *Mol Cell Biol* **28**, 2559–2566.

216 Liu CW, Wang RH, Dohadwala M, Schönthal AH, Villa-Moruzzi E & Berndt N (1999) Inhibitory phosphorylation of PP1α catalytic subunit during the G_1/S transition. *J Biol Chem* **274**, 29470–29475.

217 Perry JA & Kornbluth S (2007) Cdc25 and Wee1: analogous opposites ? *Cell Div* **2**, 12.

218 Magenta A, Fasanaro P, Romani S, Di Stefano V, Capogrossi MC & Martelli F (2008) Protein phosphatase 2A subunit PR70 interacts with pRb and mediates its dephosphorylation. *Mol Cell Biol* **28**, 873–882.

219 Ory S, Zhou M, Conrads TP, Veenstra TD & Morrison DK (2003) Protein phosphatase 2A positively regulates Ras signaling by dephosphorylating KSR1 and Raf-1 on critical 14-3-3 binding sites. *Curr Biol* **13**, 1356–1364.

220 Broude EV, Swift ME, Vivo C, Chang BD, Davis BM, Kalurupalle S, Blagosklonny MV & Roninson IB (2007) p21(Waf1/Cip1/Sdi1) mediates retinoblastoma protein degradation. *Oncogene* **26**, 6954–6958.

221 Bracken AP, Ciro M, Cocito A & Helin K (2004) E2F target genes: unraveling the biology. *Trends Biochem Sci* **29**, 409–417.

222 Berndt N, Dohadwala M & Liu CW (1997) Constitutively active protein phosphatase 1α causes Rb-dependent G_1 arrest in human cancer cells. *Curr Biol* **7**, 375–386.

223 Rubin E, Mittnacht S, Villa-Moruzzi E & Ludlow JW (2001) Site-specific and temporally-regulated retinoblastoma protein dephosphorylation by protein phosphatase type 1. *Oncogene* **20**, 3776–3785.

224 Inoue Y, Kitagawa M & Taya Y (2007) Phosphorylation of pRB at Ser612 by Chk1/2 leads to a complex between pRB and E2F-1 after DNA damage. *EMBO J* **26**, 2083–2093.

225 Classon M & Dyson N (2001) p107 and p130: versatile proteins with interesting pockets. *Exp Cell Res* **264**, 135–147.

226 Kops GJPL, Medema RH, Glassford J, Essers MAG, Dijkers PF, Coffer PJ, Lam EWF & Burgering BMT (2002) Control of Cell Cycle Exit and Entry by Protein Kinase B-Regulated Forkhead Transcription Factors. *Mol Cell Biol* **22**, 2025–2036.

227 Giacinti C & Giordano A (2006) RB and cell cycle progression. *Oncogene* **25**, 5220–5227.

228 Garriga J, Jayaraman AL, Limón A, Jayadeva G, Sotillo E, Truongcao M, Patsialou A, Wadzinski BE & na XG (2004) A dynamic equilibrium between CDKs and PP2A modulates phosphorylation of pRB, p107 and p130. *Cell Cycle* **3**, 1320–1330.

229 Leung JY, Ehmann GL, Giangrande PH & Nevins JR (2008) A role for Myc in facilitating transcription activation by E2F1. *Oncogene* **27**, 4172–4179.

230 Delavaine L & La Thangue NB (1999) Control of E2F activity by $p21^{Waf1/Cip1}$. *Oncogene* **18**, 5381–5392.

231 García-Alvarez G, Ventura V, Ros O, Aligué R, Gil J & Tauler A (2007) Glycogen synthase kinase-3β binds to E2F1 and regulates its transcriptional activity. *Biochim Biophys Acta* **1773**, 375–382.

232 Roy S, Shor AC, Bagui TK, Seto E & Pledger WJ (2008) Histone deacetylase 5 represses the transcription of cyclin D3. *J Cell Biochem* **104**, 2143–2154.

233 Zhang HJ, Li WJ, Gu YY, Li SY, An GS, Ni JH & Jia HT (2010) p14ARF interacts with E2F factors to form p14ARF-E2F/partner-DNA complexes repressing E2F-dependent transcription. *J Cell Biochem* **109**, 693–701.

234 Rizos H, Scurr LL, Irvine M, Alling NJ & Kefford RF (2007) p14ARF regulates E2F-1 ubiquitination and degradation via a p53-dependent mechanism. *Cell Cycle* **6**, 1741–1747.

235 Schuster MB & Porse BT (2006) C/EBPα: a tumour suppressor in multiple tissues? *Biochim Biophys Acta* **1766**, 88–103.

236 Massagué J, Seoane J & Wotton D (2005) Smad transcription factors. *Genes & Development* **19**, 2783–2810.

237 Massagué J (2004) G_1 cell-cycle control and cancer. *Nature* **432**, 298–306.

238 Matsuura I, Denissova NG, Wang G, He D, Long J & Liu F (2004) Cyclin-dependent kinases regulate the antiproliferative functions of Smads. *Nature* **430**, 226–231.

239 Johnson-Pais T, Degnin C & Thayer MJ (2001) pRB induces Sp1 activity by relieving inhibition mediated by MDM2. *Proc Natl Acad Sci U S A* **98**, 2211–2216.

240 Shaul Y (2000) c-Abl: activation and nuclear targets. *Cell Death & Differentiation* **7**, 10–16.

241 Martin NG, McAndrew PC, Eve PD & Garrett MD (2008) Phosphorylation of cyclin dependent kinase 4 on tyrosine 17 is mediated by Src family kinases. *FEBS J* **275**, 3099–3109.

242 Yeatman TJ (2004) A renaissance for SRC. *Nat Rev Cancer* **4**, 470–480.

243 Knudsen ES & Wang JY (1996) Differential regulation of retinoblastoma protein function by specific Cdk phosphorylation sites. *J Biol Chem* **271**, 8313–8320.

244 Zhang L & Wang C (2006) F-box protein Skp2: a novel transcriptional target of E2F. *Oncogene* **25**, 2615–2627.

245 Rodier G, Coulombe P, Tanguay PL, Boutonnet C & Meloche S (2008) Phosphorylation of Skp2 regulated by CDK2 and Cdc14B protects it from degradation by APC^{Cdh1} in G_1 phase. *The EMBO Journal* **27**, 679–691.

246 Ang XL & Harper JW (2004) Interwoven ubiquitination oscillators and control of cell cycle transitions. *Sci STKE* **2004**, pe31.

247 Burhans WC & Heintz NH (2009) The cell cycle is a redox cycle: Linking phase-specific targets to cell fate. *Free Radic Biol Med* **47**, 1282–1293.

248 Ji P, Jiang H, Rekhtman K, Bloom J, Ichetovkin M, Pagano M & Zhu L (2004) An Rb-Skp2-p27 Pathway Mediates Acute Cell Cycle Inhibition by Rb and Is Retained in a Partial-Penetrance Rb Mutant. *Mol Cell* **16**, 47–58.

249 Ji P & Zhu L (2005) Using kinetic studies to uncover new Rb functions in inhibiting cell cycle progression. *Cell Cycle* **4**, 373–375.

250 Massagué J & Gomis RR (2006) The logic of TGF-β signaling. *FEBS Lett* **580**, 2811–2820.

251 Hodge DR, Hurt EM & Farrar WL (2005) The role of IL-6 and STAT3 in inflammation and cancer. *Eur J Cancer* **41**, 2502–2512.

252 Baud V & Karin M (2001) Signal transduction by tumor necrosis factor and its relatives. *Trends in Cell Biology* **11**, 372–377.

253 Kamura T, Hara T, Matsumoto M, Ishida N, Okumura F, Hatakeyama S, Yoshida M, Nakayama K & Nakayama KI (2004) Cytoplasmic ubiquitin ligase KPC regulates proteolysis of $p27^{Kip1}$ at G_1 phase. *Nat Cell Biol* **6**, 1229–1235.

254 Solomon SS, Majumdar G, Martinez-Hernandez A & Raghow R (2008) A critical role of Sp1 transcription factor in regulating gene expression in response to insulin and other hormones. *Life Sci* **83**, 305–312.

255 Nicolás M, Noé V & Ciudad CJ (2003) Transcriptional regulation of the human Sp1 gene promoter by the specificity protein (Sp) family members nuclear factor Y (NF-Y) and E2F. *Biochem J* **371**, 265–275.

256 Tapias A, Ciudad CJ, Roninson IB & Noé V (2008) Regulation of Sp1 by cell cycle related proteins. *Cell Cycle* **7**, 2856–2867.

257 Wierød L, Rosseland CM, Lindeman B, Oksvold MP, Grøsvik H, Skarpen E & Huitfeldt HS (2007) CDK2 regulation through PI3K and CDK4 is necessary for cell cycle progression of primary rat hepatocytes. *Cell Prolif* **40**, 475–487.

258 Paternot S, Bockstaele L, Bisteau X, Kooken H, Coulonval K & Roger PP (2010) Rb inactivation in cell cycle and cancer: the puzzle of highly regulated activating phosphorylation of CDK4 versus constitutively active CDK-activating kinase. *Cell Cycle* **9**, 689–699.

259 Arora V, Knapp DC, Smith BL, Statdfield ML, Stein DA, Reddy MT, Weller DD & Iversen PL (2000) c-Myc antisense limits rat liver regeneration and indicates role for c-Myc in regulating cytochrome P-450 3A activity. *J Pharmacol Exp Ther* **292**, 921–928.

260 Buck M, Poli V, van der Geer P, Chojkier M & Hunter T (1999) Phosphorylation of rat serine 105 or mouse threonine 217 in C/EBPβ is required for hepatocyte proliferation induced by TGFα. *Mol Cell* **4**, 1087–1092.

261 Rickheim DG, Nelsen CJ, Fassett JT, Timchenko NA, Hansen LK & Albrecht JH (2002) Differential regulation of cyclins D1 and D3 in hepatocyte proliferation. *Hepatology* **36**, 30–38.

262 Bellamy CO, Clarke AR, Wyllie AH & Harrison DJ (1997) p53 deficiency in liver reduces local control of survival and proliferation, but does not affect apoptosis after DNA damage. *FASEB J* **11**, 591–599.

263 Nelsen CJ, Hansen LK, Rickheim DG, Chen C, Stanley MW, Krek W & Albrecht JH (2001) Induction of hepatocyte proliferation and liver hyperplasia by the targeted expression of cyclin E and skp2. *Oncogene* **20**, 1825–1831.

264 Rosseland CM, Wierød L, Oksvold MP, Werner H, Ostvold AC, Thoresen GH, Paulsen RE, Huitfeldt HS & Skarpen E (2005) Cytoplasmic retention of peroxide-activated ERK provides survival in primary cultures of rat hepatocytes. *Hepatology* **42**, 200–207.

265 Scheving LA, Stevenson MC, Taylormoore JM, Traxler P & Russell WE (2002) Integral role of the EGF receptor in HGF-mediated hepatocyte proliferation. *Biochem Biophys Res Commun* **290**, 197–203.

266 Wierød L (2007) *Regulation of Cell Cycle Progression and Cellular Survival in Primary Rat Hepatocytes*. Ph.D. thesis, University of Oslo, Faculty of Medecine, Laboratory for Toxicopathology, Institute of Pathology, Rikshospitalet Radiumhospitalet Medical Centre.

267 Götschel F, Kern C, Lang S, Sparna T, Markmann C, Schwager J, McNelly S, von Weizsäcker F, Laufer S, Hecht A *et al.* (2008) Inhibition of GSK3 differentially modulates NF-κB, CREB, AP-1 and β-catenin signaling in hepatocytes, but fails to promote TNF-α-induced apoptosis. *Exp Cell Res* **314**, 1351–1366.

268 Schwabe RF & Brenner DA (2002) Role of glycogen synthase kinase-3 in TNF-α-induced NF-κB activation and apoptosis in hepatocytes. *Am J Physiol Gastrointest Liver Physiol* **283**, G204–G211.

269 Nelsen CJ, Rickheim DG, Timchenko NA, Stanley MW & Albrecht JH (2001) Transient Expression of Cyclin D1 Is Sufficient to Promote Hepatocyte Replication and Liver Growth *in Vivo*. *Cancer Res* **61**, 8564–8568.

270 Albrecht JH & Hansen LK (1999) Cyclin D1 Promotes Mitogen-independent Cell cycle Progression in Hepatocytes. *Cell Growth & Differentiation* **10**, 397–404.
